# Comparative genomics reveal shared genomic changes in syngnathid fishes and signatures of genetic convergence with placental mammals

**DOI:** 10.1093/nsr/nwaa002

**Published:** 2020-01-09

**Authors:** Yan-Hong Zhang, Vydianathan Ravi, Geng Qin, He Dai, Hui-Xian Zhang, Feng-Ming Han, Xin Wang, Yu-Hong Liu, Jian-Ping Yin, Liang-Min Huang, Byrappa Venkatesh, Qiang Lin

**Affiliations:** CAS Key Laboratory of Tropical Marine Bio-resources and Ecology, South China Sea Institute of Oceanology, Innovation Academy of South China Sea Ecology and Environmental Engineering, Chinese Academy of Sciences, Guangzhou 510301, China; Comparative and Medical Genomics Laboratory, Institute of Molecular and Cell Biology, A*STAR 138673, Singapore; CAS Key Laboratory of Tropical Marine Bio-resources and Ecology, South China Sea Institute of Oceanology, Innovation Academy of South China Sea Ecology and Environmental Engineering, Chinese Academy of Sciences, Guangzhou 510301, China; Southern Marine Science and Engineering Guangdong Laboratory (Guangzhou), Guangzhou 511458, China; Biomarker Technologies Corporation, Beijing 101300, China; CAS Key Laboratory of Tropical Marine Bio-resources and Ecology, South China Sea Institute of Oceanology, Innovation Academy of South China Sea Ecology and Environmental Engineering, Chinese Academy of Sciences, Guangzhou 510301, China; Biomarker Technologies Corporation, Beijing 101300, China; CAS Key Laboratory of Tropical Marine Bio-resources and Ecology, South China Sea Institute of Oceanology, Innovation Academy of South China Sea Ecology and Environmental Engineering, Chinese Academy of Sciences, Guangzhou 510301, China; CAS Key Laboratory of Tropical Marine Bio-resources and Ecology, South China Sea Institute of Oceanology, Innovation Academy of South China Sea Ecology and Environmental Engineering, Chinese Academy of Sciences, Guangzhou 510301, China; CAS Key Laboratory of Tropical Marine Bio-resources and Ecology, South China Sea Institute of Oceanology, Innovation Academy of South China Sea Ecology and Environmental Engineering, Chinese Academy of Sciences, Guangzhou 510301, China; Southern Marine Science and Engineering Guangdong Laboratory (Guangzhou), Guangzhou 511458, China; CAS Key Laboratory of Tropical Marine Bio-resources and Ecology, South China Sea Institute of Oceanology, Innovation Academy of South China Sea Ecology and Environmental Engineering, Chinese Academy of Sciences, Guangzhou 510301, China; University of Chinese Academy of Sciences, Beijing 100049, China; Comparative and Medical Genomics Laboratory, Institute of Molecular and Cell Biology, A*STAR 138673, Singapore; CAS Key Laboratory of Tropical Marine Bio-resources and Ecology, South China Sea Institute of Oceanology, Innovation Academy of South China Sea Ecology and Environmental Engineering, Chinese Academy of Sciences, Guangzhou 510301, China; Southern Marine Science and Engineering Guangdong Laboratory (Guangzhou), Guangzhou 511458, China; University of Chinese Academy of Sciences, Beijing 100049, China

**Keywords:** Manado pipefish, whole-genome sequencing, transposable elements, placenta-like structure, convergent evolution

## Abstract

Syngnathids (seahorses, pipefishes and seadragons) exhibit an array of morphological innovations including loss of pelvic fins, a toothless tubular mouth and male pregnancy. They comprise two subfamilies: Syngnathinae and Nerophinae. Genomes of three Syngnathinae members have been analyzed previously. In this study, we have sequenced the genome of a Nerophinae member, the Manado pipefish (*Microphis manadensis*), which has a semi-enclosed brood pouch. Comparative genomic analysis revealed that the molecular evolutionary rate of the four syngnathids is higher than that of other teleosts. The loss of all but one P/Q-rich SCPP gene in the syngnathids suggests a role for the lost genes in dentin and enameloid formation in teleosts. Genome-wide comparison identified a set of 118 genes with parallel identical amino acid substitutions in syngnathids and placental mammals. Association of some of these genes with placental and embryonic development in mammals suggests a role for them in syngnathid pregnancy.

## INTRODUCTION

**Figure 1.**
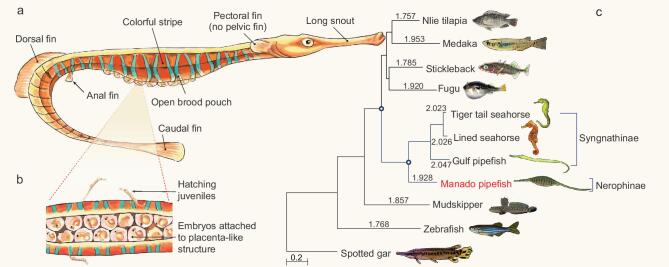
Morphological features and evolutionary rate of the Manado pipefish (*M. manadensis*). (a) Schematic diagram of a male pipefish showing the morphological features. (b) Ventral view of the open brood pouch. The Manado pipefish has a semi-enclosed brood pouch like the Gulf pipefish. (c) A neutral tree of 11 ray-finned fishes based on 4-fold degenerate (4D) sites showing the higher evolutionary rate of syngnathids. Values on the branches represent pairwise distance for each species to the outgroup (spotted gar). The scale bar denotes the number of substitutions per 4D site.

The family Syngnathidae (seahorses, pipefishes and seadragons) is a morphologically unique group of teleosts comprising 57 genera and 319 species (*Eschmeyer's Catalog of Fishes*, https://www.calacademy.org/scientists/projects/catalog-of-fishes). Their specialized morphology includes an elongated tube-shaped mouth lacking teeth, an elongated body covered with bony plates, absence of pelvic fins and scales, and a specialized brood pouch in males [1,2]. The design of the male brood pouch varies from simple ‘gluing’ of eggs to a patch of skin on the abdomen or tail as in some pipefishes (e.g. the alligator pipefish, *Syngnathoides biaculeatus*) and seadragons (e.g. the weedy seadragon, *Phyllopteryx taeniolatus*), to a partially covered brood pouch in some pipefishes (e.g. the half-banded pipefish, *Mitotichthys semistriatus*), and a highly specialized closed brood pouch in seahorses (see Supplementary Fig. S1.1). Syngnathids also exhibit wide diversity in their morphology and coloration (see Supplementary Fig. S1.1). In addition, some members like seahorses possess further derived features such as loss of caudal fin, a vertical posture and a prehensile tail. Thus, besides a set of shared specialized features, syngnathids also exhibit a gradient of additional derived morphological features. Recent phylogenetic analyses have revealed two distinct clades within the syngnathids, the tail-brooders and the trunk-brooders representing sub-families Nerophinae (trunk-brooding pipefishes) and Syngnathinae (tail-brooders comprising pipefishes, seadragons, pygmy pipehorses and seahorses), respectively [3,4] (see Supplementary Fig. S1.1). This classification scheme implies independent origins of the complex male brooding structure within the family Syngnathidae. To understand the genetic basis of the specialized morphology of syngnathids, whole-genomes of three members of the subfamily Syngnathinae, the tiger tail seahorse (*Hippocampus comes*) [5], the lined seahorse (*Hippocampus erectus*) [6] and the Gulf pipefish (*Syngnathus scovelli*) [7] have been sequenced and analyzed previously. The genome of a Nerophinae member would be valuable for understanding the genetic bases of the common specialized phenotypes of syngnathids besides serving as a useful outgroup for elucidating genetic changes associated with lineage-specific features of seahorses and other Syngnathinae members. In this study, we have sequenced the genome of a Nerophinae member, the Manado pipefish (*Microphis manadensis*), which possesses a semi-enclosed brood pouch (Fig. 1a and b), and have compared it with the genomes of the three Syngnathinae members as well as other representative teleosts.

‘Male pregnancy’ in syngnathids, whereby embryos are incubated in a brood pouch, is unique among vertebrates. Females transfer eggs directly into this specialized pouch which is derived from the abdominal epithelium of males [8]. The brood pouch is a complex organ composed of highly vascularized epithelial tissue, and is functionally equivalent to the mammalian uterus. During gestation, the brood pouch undergoes significant changes in morphology, including thickening, vascularization, development of epithelial microridges for embryo implantation, and overall restructuring forming a placenta-like tissue [1]. Thus, the male brood pouch of syngnathids and the uterus of placental mammals represent an interesting instance of convergent evolution. A detailed analysis of the male brood pouch transcriptome of a seahorse (the big-belly seahorse, *Hippocampus abdominalis*) from different stages of pregnancy has indicated that the seahorse brood pouch expresses homologs of mammalian genes that are involved in mammalian placental growth, nutrient and waste transport, gas exchange, osmoregulation and immune protection during pregnancy [9]. This finding indicates that the same repertoire of genes was coopted during the evolution of pregnancy in male seahorses and placental mammals.

Adaptive phenotypic convergence between distantly related taxa has occurred repeatedly in nature and has been attributed to convergent genetic changes driven by similar selective pressures. Some recent whole-genome comparisons of animals exhibiting convergent adaptive phenotypic evolution have uncovered genome-wide signatures of genetic convergence including identical amino acid substitutions in orthologous genes [10,11]. However, the results are often controversial [12,13]. For example, the number of species and genomes used in an analysis can influence the probability of detecting convergent residues. Another reason for the uncertainty is that molecular convergence is a noisy process. To overcome these drawbacks, researchers developed a new method which involves identification of conservative sites, followed by detection of convergence at these sites. This method drastically reduces the likelihood of potential random convergence at rapidly evolving sites as well as falsely inferred convergence caused by the mis-inference of the ancestral character [12]. Using this method, the authors found 5830 and 4580 convergent amino acid substitutions in mangrove and non-mangrove plants, respectively. In this study, we use a similar approach to identify genetic signatures of convergent evolution related to pregnancy in syngnathids and placental mammals through comparative genomics of 16 distantly related vertebrates including four syngnathids and four placental mammals. Our analysis uncovered a set of 118 genes in the two groups with convergent accumulation of identical amino acids, including some genes known to be associated with placental and embryonic development in mammals.

## RESULTS

### Genome sequencing, assembly and annotation

The Manado pipefish (*M. manadensis*) genome was sequenced using DNA from a male individual. Libraries with insert sizes ranging from 200 bp to 17 kb were sequenced on the Illumina HiSeq 2500 platform, and 102.02 Gb of sequences filtered for low quality and duplicate reads were generated. These sequences were assembled using ALLPATHS-LG to yield a 653 Mb assembly of the estimated 686 Mb genome (Supplementary Information section 2) with N50 contig and scaffold sizes of 68 kb and 2.75 Mb, respectively. Completeness of genome was assessed using CEGMA (Core Eukaryotic Genes Mapping Approach) and BUSCO (Benchmarking Universal Single Copy Orthologs). CEGMA evaluation found that 246 (99%) of 248 core eukaryotic genes were complete whereas BUSCO analysis showed that 92% of the single-copy orthologues were complete. A total of 21 003 protein-coding genes were predicted in the assembly by combining homology-based and *Abinitio* gene prediction methods, with 94% of the gene models supported by RNA-seq transcripts (see Supplementary Information section 4).

### Phylogenomics and evolutionary rate

To verify the phylogenetic position of the Manado pipefish (*M. manadensis*, a member of Nerophinae) with respect to Syngnathinae, we used the genome-scale dataset of the Manado pipefish along with datasets from other fishes (including the three Syngnathinae members) to perform phylogenomic analysis. We identified a set of 2634 high-confidence, one-to-one orthologues present in each of the 11 selected fishes (see Supplementary Information section 5) and carried out phylogenomic analyses using protein as well as coding sequences. The two datasets gave identical topologies placing the Manado pipefish, a trunk-brooder, as an outgroup to the three tail-brooders (Syngnathinae) with maximal support (see Fig. 1c and Supplementary Fig. S5.3), consistent with the previous molecular phylogeny [3,4].

Previous analysis of molecular evolutionary rate in the tiger tail seahorse (*H. comes*) had indicated that its protein and nucleotide sequences were evolving at a higher rate than other non-syngnathid teleosts [5]. We analyzed the molecular evolutionary rate of the four syngnathids (three Syngnathinae members and a Nerophinae member) and other fishes using the phylogenomic dataset. The analysis showed that protein as well as nucleotide sequences of all four syngnathid members are evolving at higher rates compared to other teleosts (see Fig. 1c and Supplementary Table S5.1, respectively), indicating that the accelerated molecular evolutionary rate is a common feature of the family Syngnathidae. Among the syngnathids, members of the subfamily Syngnathinae showed higher protein and neutral evolutionary rates than the Manado pipefish (*M. manadensis*), a member of the subfamily Nerophinae (see Fig. 1c, Supplementary Tables S5.1 and 5.2). The higher molecular evolutionary rate in Syngnathinae, which includes 263 species and diverse fishes such as seahorses, pipefishes and seadragons, may be related to their greater phenotypic diversity compared to Nerophinae, which comprises 56 species composed of only pipefishes.

### Transposable elements in syngnathids

**Figure 2.**
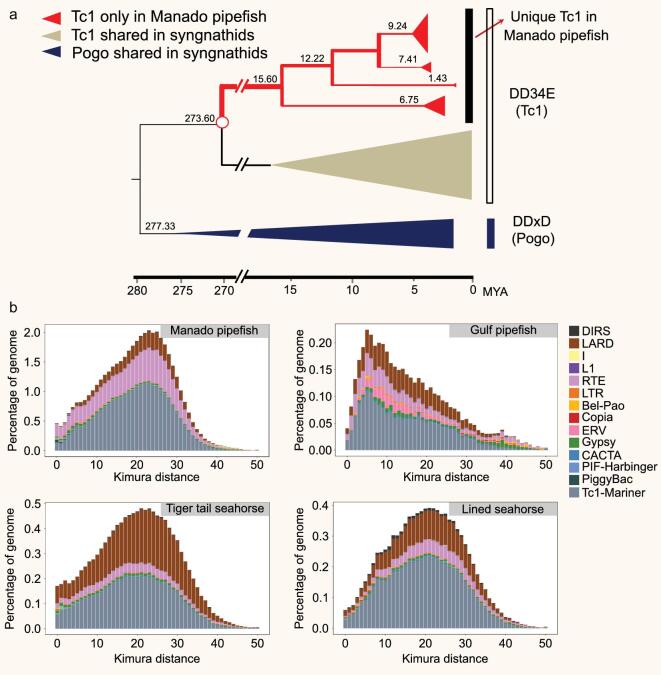
Transposable elements (TEs) in syngnathids. (a) Dating the Tc1/mariner subfamily. Each group is shown as a triangle, where the horizontal distance is proportional to the age and the vertical distance is proportional to the number of sequences in the group. (b) Kimura distance-based copy divergence analysis of TEs in syngnathids. The x-axis indicates a specific TE family at a given divergence from the consensus sequence and y-axis indicates its percentage in the genome.

Transposable elements (TEs) constitute a major fraction of eukaryotic genomes and play an important role in determining the genome size, chromosomal rearrangements and architecture, and gene regulation [14,15]. The Manado pipefish (*M. manadensis*) genome, which is the largest of the four syngnathids analyzed, contains the highest level of TEs and other interspersed repeats (53%), followed by the lined seahorse (28%), and the tiger-tail seahorse (26%). The repeat content accounts for only 12% of the Gulf pipefish (*S. scovelli*) genome, which is the smallest among the four (see Supplementary Table S4.1). Thus, differential accumulation of TEs appears to have contributed to the variation in genome sizes of the four syngnathid genomes. In particular, Tc1/mariner was found to be the most abundant DNA transposon superfamily in the Manado pipefish genome (33%; 215.98 Mb) (see Supplementary Table S4.2). Tc1 transposons were found to have undergone striking expansion in the Manado pipefish lineage around 15.60 million years ago (Mya; see Fig. 2a), after the split of the Syngnathinae and Nerophinae subfamilies approximately 50 Mya [16]. In general, TEs within the genome of the Manado pipefish have remained active over a long evolutionary period as shown by the broad distribution of divergence levels ranging from 0 to 45% with a sharp peak at about 23% (see Fig. 2b). In contrast, the activity and accumulation of TEs in the Gulf pipefish have been much lower, with a divergence peak of only 6%. Overall, the divergence analysis shows that most of the TEs in the Manado pipefish experienced one round of recent expansion, followed by a decrease in activity. In contrast, TEs in the other three syngnathid genomes experienced a low level of proliferation.

### Expansion and contraction of gene families in the Manado pipefish

Analysis of gene family evolution using a maximum likelihood framework identified significant expansion of 27 gene families and contraction of 163 families in the Manado pipefish (*M. manadensis*) lineage, whereas the common ancestor of syngnathids possessed one significantly expanded and 154 significantly contracted gene families (see Supplementary Fig. S5.3). We noted that the Manado pipefish expanded gene family set contained four gene families (GF_1013, GF_14, GF_10551 and GF_447; Supplementary Table S5.3) annotated as ‘peptidase family M50’, which are unusually expanded in the Manado pipefish compared with other teleosts. The M50 family of proteases is composed of strictly membrane-bound metallo-endopeptidases, which are involved in regulation of gene expression through processing of various transcription factors such as the mammalian S2P (sterol regulatory element-binding protein (SREBP) Site-2 protease, S2P) proteases [17]. It is not clear how the expansion of this gene family is related to the biology of the Manado pipefish. Another expanded gene family was the E2F-associated phosphoprotein (GF_2250, Supplementary Table S5.3). These genes are believed to be important for fine-tuning of E2F1 functions such as the induction of apoptosis and cell-cycle regulation [18]. Within the contracted gene family set, we found cytochrome P450 and NACHT domain proteins that were contracted in the Manado pipefish (Supplementary Table S5.4). Cytochrome P450 is a family of enzymes involved in the synthesis of molecules such as hormones, fatty acids and bile acids, and the breakdown and clearance of various compounds such as drugs and xenobiotics [19]. The NACHT domain is found in proteins involved in apoptosis and MHC transcription [20]. However, it is unclear how the contraction of these gene families is related to the unique biology of the Manado pipefish. In addition, analysis of the 154 contracted gene families in the common ancestor of syngnathids revealed that 35 of them are annotated as ‘immunoglobulin domain’, and nine of them are annotated as ‘lectin C-type domain’ (Supplementary Table S5.5). The immunoglobulin domain is found in a large number of proteins with varied functions such as antibodies and kinases, and is involved in protein-protein and protein-ligand interactions [21]. C-type lectin domain-containing proteins are a large group of extracellular proteins with diverse functions. These proteins are mainly involved in calcium-dependent carbohydrate binding, but can also bind to other proteins, lipids and inorganic molecules. They function as adhesion and signaling receptors for immune-related functions such as inflammation [22]. It is intriguing why these gene families were contracted in the common ancestor of all syngnathids.

### Loss of SCPP genes in syngnathids

**Figure 3.**
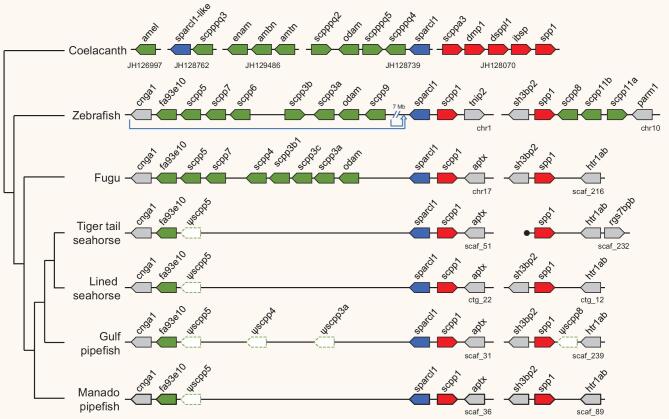
SCPP genes in four syngnathids (Manado pipefish, *M. manadensis*; Gulf pipefish, *S. scovelli*; lined seahorse, *H. erectus;* and tiger tail seahorse, *H. comes*) and other representative fishes. Gene loci for coelacanth and zebrafish were adapted from [24]. P/Q-rich SCPP genes are shown in green and acidic SCPP genes are shown in red. The related *SPARCL1* and *SPARCL1*-like genes are shown in blue. Because of space constraints, genes *gsp37* and *scpp12* in zebrafish (P/Q-rich SCPP genes) have been omitted. Black circle marks the end of scaffold.

Syngnathids as a family display a phenomenon known as edentulism wherein they completely lack teeth. Feeding in these fishes is suction-based using their tubular snout-like mouths. Genes belonging to the SCPP (secretory calcium-binding phosphoprotein) gene family have been implicated in formation of mineralized tissues such as bone, dentin, enamel and enameloid [23]. Based on their amino acid composition, SCPP genes are classified as acidic or P/Q-rich SCPP genes. Acidic SCPP genes are involved in formation of bone and dentin, whereas P/Q-rich SCPP genes are required for enamel or enameloid formation. Teleosts in general possess two acidic SCPP genes and lineage-specific sets of P/Q-rich SCPP genes ranging from eight genes in fugu (*Takifugu rubripes*) to at least 13 genes in zebrafish (*Danio rerio*) [24] (see Fig. 3). Previous studies have implicated the role of inactivating mutations in enamel- and dentin-specific genes in the loss of teeth in modern birds (Neornithes), some turtles (Western painted turtle, green sea turtle and Chinese soft-shelled turtle) and some toothless mammals (nine-banded armadillo, Hoffmann's two-toed sloth, aardvark and Chinese pangolin) [25,26].

We have previously shown that the tiger tail seahorse (*H. comes*) genome possesses two acidic SCPP genes and lacks intact P/Q-rich SCPP genes; and proposed that the loss of P/Q-rich SCPP genes might be related to the loss of teeth in this species [5]. In the present study we analyzed SCPP genes in the other three syngnathids, the Manado pipefish (*M. manadensis*), Gulf pipefish (*S. scovelli*) and the lined seahorse (*H. erectus*) genomes, and found that all contain two intact acidic SCPP genes, *scpp1* and *spp1*, like the tiger tail seahorse (see Fig. 3). In addition, a P/Q-rich SCPP gene, *fa93e10*, which was not found previously in the tiger tail seahorse, was also found in these three syngnathid fishes (see Fig. 3). We used the predicted protein sequence of the Manado pipefish *fa93e10* to search the tiger tail seahorse genome and did indeed identify the *fa93e10* ortholog in the tiger tail seahorse (see Fig. 3). Thus, members of both sub-families within Syngnathidae, Nerophinae (Manado pipefish) and Syngnathinae (tiger tail seahorse, lined seahorse and Gulf pipefish), possess two acidic SCPP genes (*scpp1* and *spp1*) and only a single P/Q-rich SCPP gene (*fa93e10*). This indicates that the remaining P/Q-rich SCPP genes were inactivated (pseudogenized) in their common ancestor prior to the speciation and diversification of syngnathids. Our present finding, that the majority of P/Q-rich SCPP genes are lost in syngnathids, provides strong support to the hypothesis that the loss of these genes explains edentulism in Syngnathidae. The only P/Q-rich SCPP gene present in the syngnathids is *fa93e10*. In zebrafish, this gene has been proposed to be a growth marker that identifies cycles of growth in fin ray segments [27]. Other studies have proposed *fa93e10* to be a divergent teleost ortholog of the *ENAM* gene based on its weak similarity to the N-terminal part of the gar enam protein [28,29]. However, as teleosts do not have enamel or ganoin on their teeth or scales, *fa93e10* is unlikely to be involved in the formation of teeth or scales in teleosts.

### Loss of *tbx4* in syngnathids

**Figure 4.**
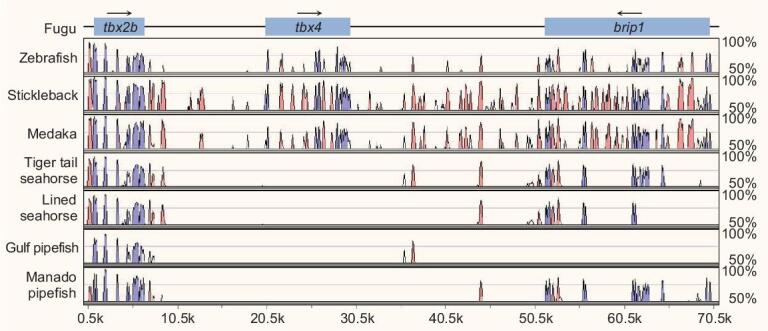
Loss of *tbx4* gene in syngnathids. VISTA plot of the MLAGAN alignment of the *tbx2b-tbx4-brip1* locus from four syngnathids (tiger tail seahorse, *H. comes*; lined seahorse, *H. erectus*; Gulf pipefish, *S. scovelli*; and Manado pipefish, *M. manadensis*) and other representative teleosts. The fugu sequence was used as the base (x-axis) and conserved sequences were predicted using a criterion of ≥70% identity across >100 bp windows. The y-axis represents percent sequence identity. Blue peaks represent conserved exons whereas pink peaks represent conserved noncoding elements. The *tbx4* gene loci in tiger tail seahorse, lined seahorse, Gulf pipefish and Manado pipefish are present on scaffold_291, scaffold_325, scaffold_493 and scaffold_159, respectively. Gulf pipefish *brip1* is located on two separate scaffolds (scaffold_584 and scaffold_610) and was therefore not included in the alignment.

All members of the family Syngnathidae lack pelvic fins [30], which are homologous to hindlimbs in terrestrial animals. We previously showed that the tiger tail seahorse (*H. comes*) genome lacks the *tbx4* gene [5], a key transcription factor involved in regulation of hindlimb formation in mammals [31]. Furthermore, CRISPR-Cas9-mediated knockout of *tbx4* in zebrafish resulted in a ‘pelvic fin-loss’ phenotype [5], suggesting that the loss of *tbx4* was related to the loss of pelvic fins in the tiger tail seahorse. *tbx4* was missing in the genome assembly of the Gulf pipefish (*S. scovelli*) [7] as well. We analyzed the *tbx2b-tbx4-brip1* locus in the Manado pipefish (*M. manadensis*) and lined seahorse (*H. erectus*) and found that the *tbx4* coding sequence was missing in both syngnathids (see Fig. 4). These findings indicate that *tbx4* was lost in the common ancestor of syngnathids prior to the divergence of Syngnathinae and Nerophinae lineages. These data provide further support to the proposal that loss of pelvic fins in members of the Syngnathidae family is related to the loss of *tbx4*.

### Expansion of nonclustered protocadherins in syngnathids

**Figure 5.**
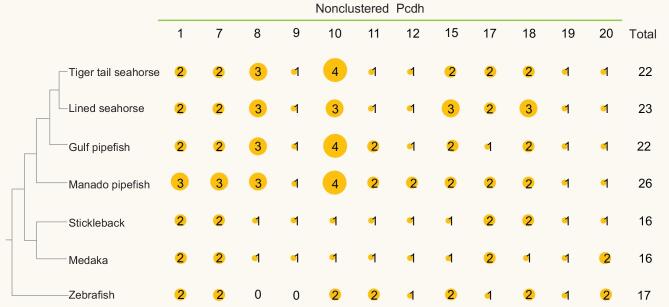
Nonclustered Pcdhs in the Manado pipefish (*M. manadensis*) and other teleosts. Distribution of nonclustered Pcdh genes in Manado pipefish and other teleosts. Sizes of the orange circles correspond to the number of nonclustered Pcdh genes present.

Protocadherins (Pcdhs) are a group of transmembrane proteins belonging to the cadherin superfamily that are classified into two subgroups – clustered Pcdhs and nonclustered Pcdhs. The clustered Pcdhs are expressed mainly in the brain and play diverse roles in neurodevelopment [32]. They also provide the molecular basis for the extraordinary diversity of the nervous system through a combinatorial expression of isoforms [33]. Nonclustered Pcdhs function as mediators of cell–cell adhesion and are involved in dynamic cellular processes such as cell sorting and cell movement during embryonic gastrulation, differentiation of the embryonic ectoderm and neural tube formation [34,35]. All the four syngnathids contain a higher number of nonclustered Pcdhs (22–26) compared with other teleosts (16–17) (see Fig. 5).

In particular, syngnathids possess three *Pcdh8* genes and three to four *Pcdh10* genes, compared with one each in other teleosts (see Fig. 5). In mammals and birds, *Pcdh8* is expressed in a spatiotemporal-specific pattern during embryogenesis, and is involved in formation and polarity of developing structures in the embryo such as the cochlea and feather buds [36,37]. Inhibition of *Pcdh8* expression by miR-429 in mouse was found to result in a significant decrease in the number of implantation sites, suggesting that both genes play a major role in embryo implantation [38]. The higher copy number of *Pcdh8* gene in syngnathids suggests that it may potentially play a role in creating implantation sites in the male brood pouch. We noted a higher expression level for *Pcdh8b1* in the pregnant brood pouch compared with the non-pregnant brood pouch (see Supplementary Fig. S6.4). In addition, several other duplicated Pcdh genes (*Pcdh1b2*, *Pcdh12a1*, *Pcdh12a2* and *Pcdh17a*) also showed higher expression in the pregnant brood pouch compared with the non-pregnant brood pouch (Supplementary Fig. S6.4), suggesting a role for these genes in the placenta. The role of another expanded gene, *Pcdh10*, and the effect of its expansion on the biology of the syngnathids is not clear.

### Genomic signatures of convergent evolution in syngnathids and placental mammals

**Figure 6.**
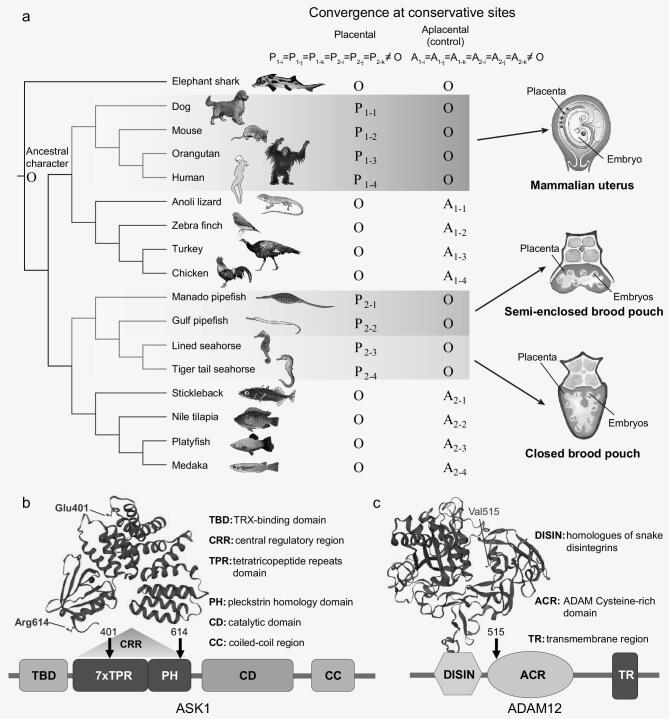
Convergent evolution of genes in syngnathids and placental mammals. (a) Left panel: the phylogeny consists of placental-aplacental pairs as ingroup species, along with an outgroup species. O indicates the character state of outgroup. P_i_ and A_i_ indicate the observed character state in placental (red) and aplacental (blue) species, respectively. Right panel: schematic diagram of the mammalian placenta and syngnathid placenta-like structure. (b) Convergent amino acid substitutions in the ASK1 protein in placental mammals and syngnathids. (c) Convergent amino acid substitutions in the ADAM12 protein in placental mammals and syngnathids.

The extent of male parental care among syngnathids ranges from simple ‘gluing’ of eggs to a patch of skin on the abdomen or tail to the highly specialized closed brood pouch. Nevertheless, a common feature of syngnathids is the presence of a placenta-like structure to which the embryos attach. Such a structure is absent in ovoviviparous teleosts such as the platyfish (*Xiphophorus maculatus*). The adherent placenta-like structure of syngnathids, which permits post-fertilization paternal provisioning, is analogous to the placenta in mammals (see Fig. 6a) and constitutes an interesting instance of convergent evolution between syngnathid teleosts and placental mammals. To explore if there were any genomic signatures associated with this convergent feature, we applied the convergence at conservative sites (CCS) method [12] to identify genes that have experienced convergence over the background level of convergence in aplacental relatives. In view of the long evolutionary distance (∼420 million years) between syngnathid teleosts and placental mammals, we selected species with the same phylogenetic topology as the ingroup control clusters (cluster 1 for placental mammals and cluster 2 for syngnathid teleosts, see Fig. 6a). We chose the oviparous elephant shark as outgroup. We identified a set of 2912 orthologous genes from 17 species including four syngnathids, four placental mammals, their respective aplacental ingroups (eight species in total) and the outgroup elephant shark (see Fig. 6a) based on syntenic pairwise alignments and reciprocal best alignments. The criteria for inferring convergence are given in Fig. 6a, where the inference is symmetric between placental species and aplacental species. At conservative sites, either the eight aplacental (ingroup) species or the syngnathid teleosts and placental mammals have the same character as the outgroup; that is A_1–1_ = A_1–2_ = A_1–3_ = A_1–4_ = A_2–1_ = A_2–2_ = A_2–3_ = A_2–4_ = O or P_1–1_ = P_1–2_ = P_1–3_ = P_1–4_ = P_2–1_ = P_2–2_ = P_2–3_ = P_2–4_ = O. If the criteria are met, we assumed that the ancestral states of both the clades are the same as the outgroup. This assumption is true in nearly all conservative sites, as we show below. With ancestors inferred as O, convergence can be inferred if at least six of the eight other species share a derived character that is different from the ancestral state, that is P_1-i_ = P_1-j_ = P_1-k_ = P_2-i_ = P_2-j_ = P_2-k_≠O or A_1-i_ = A_1-j_ = A_1-k_ = A_2-i_ = A_2-j_ = A_2-k_≠O.

As a result, we found 125 and 36 convergent amino acid substitutions in the foreground and background clusters, respectively. Most convergent genes possess a single convergent site, whereas six genes were identified with two or more convergent sites in syngnathid teleosts and placental mammals. The convergence level in syngnathid teleosts and placental mammals was found to be significantly higher than that of aplacental species (*P* < 0.05, using the χ^2^ test, Supplementary Table S8.2).

We performed GO and KEGG enrichment analysis of the convergent gene set, but identified only one GO term that was significantly enriched (Cellular Component: COPII vesicle coat, FDR < 0.05, Supplementary Table S8.4). Nevertheless, the set of convergent genes identified in placental mammals and syngnathid teleosts includes some genes that might be associated with placental and embryonic development in mammals. These include *ASK1* (apoptosis signal-regulating kinase 1), also known as *MAP3K5* (mitogen-activated protein kinase kinase 5), a member of the MAPK kinase (MAPKKK) family implicated in cytokine- and stress-induced apoptosis [39]. Its localized expression in the developing mouse embryo skin, cartilage and bone suggests a role for the gene in tissue development during embryogenesis [40]. Furthermore, it also plays a role in angiogenesis as the IL6-induced activation of VEGF, which contributes to angiogenesis in human osteosarcoma cells, is mediated through the ASK1/p38/AP-1 pathway [41]. Two convergent amino acid substitutions, D401E and K614R, were found in all four syngnathids as well as placental mammals located in the central regulatory region (CRR) of the ASK1 protein (see Fig. 6b). Biochemical assays have shown that a conserved region within the CRR is required for ASK1 activity. It is possible that *ASK1* plays a role in placental angiogenesis, which is important for placental development and morphogenesis. Another gene identified in the set was *ADAM12* (ADAM metallopeptidase domain 12), which belongs to a family of proteins implicated in various biological processes including fertilization, muscle development and neurogenesis. Tissue-specific expression analyses using all major human organs have shown that this gene has enhanced expression in the placenta compared with 26 other tissues [42]. In addition, it has been suggested that *ADAM12* is involved in regulation of endothelial cell function and that its expression is positively correlated with VEGF and MMP-9, which are pro-angiogenic factors [43]. A single convergent site (I515V) is located in the ADAM cysteine rich domain (ACR, see Fig. 6c) and might be related to placental development and angiogenesis in syngnathids and placental mammals. The human orthologues of two other convergent genes, glial cell line-derived neurotrophic factor (*GDNF*) and Vasohibin-1 (*VASH1*), show high expression levels in the placenta in addition to other tissues [42]. Both these genes have been shown to be involved in angiogenesis. Whereas *GDNF* has been shown to promote angiogenesis in human umbilical vein endothelial cells [44], *VASH1* was isolated from endothelial cells as a negative feedback regulator of angiogenesis [45]. Angiogenesis, or the formation of neovessels, is regulated by the local balance between angiogenesis stimulators and inhibitors, which is important for placental development and morphogenesis. Another gene, *NDRG1* (N-myc downstream regulated 1), which contains two convergent sites (I206V and V293I), is involved in development, embryogenesis, cell growth and differentiation besides other functions [46]. Another gene containing two convergent sites (Q350H and L351V) is the *SALL1* gene (spalt like transcription factor 1), which belongs to the SALL family, members of which function as critical regulators in development of multiple mammalian organs, including kidney, heart and the hematopoietic system. *SALL1* is required during embryonic kidney development and stem cell differentiation [47]. The convergent gene list also includes *MED1* (mediator complex subunit 1; G1284S), which has been shown to be involved in decidualization, that is transformation of endometrial cells in preparation for pregnancy [48].

## DISCUSSION

Syngnathids as a group exhibit one of the most diverse and complex array of morphological innovations. While some of the features (e.g. loss of pelvic fins, teeth and scales) are common to all members of the family, some syngnathid lineages have acquired additional derived features resulting in a gradient of innovations leading to the highly specialized morphology seen in seahorses. Syngnathids are divided into two subfamilies, the Syngnathinae and Nerophinae, which respectively comprise the tail-brooders (i.e. brood pouch located on the tail) and the trunk-brooders (i.e. brood pouch in the trunk region), with the exception of a trunk-brooder, the alligator pipefish (*Syngnathoides biaculeatus*) which clusters with tail-brooding Syngnathinae [3,4]. As varying complexities of brood pouch structures are found in both subfamilies, this classification scheme implies that the complexity of the brood pouch has evolved independently in the two syngnathid lineages. The combination of a common specialized phenotype and a gradient of lineage-specific phenotypes make syngnathids a valuable group for understanding the genetic bases of a gradient of phenotypic innovations. Previously, genomes of only Syngnathinae members were sequenced and compared with those of other teleosts. In this study, we have sequenced and analyzed the genome of a Nerophinae member, the Manado pipefish (*M. manadensis*), to serve as a reference genome for unravelling the genetic changes in the common ancestor of syngnathids as well as in the different syngnathid lineages.

The protein and nucleotide sequences of the syngnathids analyzed were found to be evolving faster than those in other teleosts, suggesting that the highly derived phenotype of this group as a whole may be related to their elevated molecular evolutionary rate. A common feature of syngnathids is loss of teeth concordant with the evolution of a tube-like mouth which is used to suck tiny planktonic food items. In mammals, acidic SCPPs are primarily involved in bone and dentin mineralization, whereas P/Q-rich SCPPs enable mineralization of enamel. However, teleosts use distinct sets of SCPP genes for mineralization of dentin and enameloid (the teleost equivalent of enamel) [49]. In the pufferfish (fugu), P/Q-rich SCPP genes *scpp2* (also known as *odam*) and *scpp4* (see Fig. 3) are exclusively expressed by the inner layer of dental epithelium and have been proposed to be critical for cap enameloid [49]. On the other hand *scpp5*, another P/Q-rich SCPP gene, is important for mineralization of both enameloid and dentin [49]. However, the roles of the multiple P/Q-rich SCPP genes present in fugu and other teleosts (see Fig. 3) in dentin and enameloid formation are not known. As syngnathids have lost all P/Q-rich SCPP genes except *fa93e10*, we can infer that the P/Q-rich SCPP genes lost have an important role in teeth formation through their function in mineralization of dentin and enameloid. Future work on dentin and enameloid formation in teleosts can focus on this set of genes.

The convergent evolution of pregnancy in placental mammals and syngnathids, with placental tissue in the former and a placenta-like structure in the latter, offered an interesting opportunity to investigate whether their genomes contain genetic signatures of convergent evolution related to pregnancy. Previous genome-wide comparisons aimed at uncovering genetic signatures of convergent phenotypic evolution in vertebrates have focused on different clades of marine mammals [10] as well as between species belonging to different families (giant panda and red panda) [11]. In contrast, the two groups analyzed in our study, the syngnathid teleosts and placental mammals, are much more distantly related having shared a common ancestor more than 400 million years ago and differ markedly in their morphological, physiological and genetic attributes. Despite this, our analysis, which uses a stringent methodology, was able to identify a set of 118 genes across the two groups possessing identical convergent substitutions. Although the direct contribution of these convergent amino acid substitutions towards the convergent phenotype, that is pregnancy, is unclear, association of some of these genes with placental and embryonic development in mammals suggests that they might be involved in pregnancy in both syngnathids and placental mammals. As such, they may represent a core set of convergent genetic changes that have evolved independently in the two lineages in response to similar selective pressure in the two groups.

## METHODS

### Genome sequencing and assembly

Genomic DNA from a single male *M. manadensis* was used to construct 14 libraries, including short-insert (200 bp, 220 bp, 500 bp) and mate-paired (3 kb, 4 kb, 8 kb, 10 kb, 15 kb, 17 kb) libraries which were sequenced on the Illumina HiSeq 2500 sequencing platform. In total, we obtained around 184.95 Gb of raw sequence data (Supplementary Table S2.1). The genome was assembled using ALLPATHS-LG with default parameters.

### RNA sequencing and analysis

In total, 21 RNA-seq libraries were constructed, including one library from combined soft tissues (brain, gills, intestine, liver and muscle) from a male *M. manadensis* (Supplementary Table S3.1); and 20 libraries from different stages of brood pouch development such as non-pregnant and pregnant stages. All libraries were prepared using the NEBNext^®^ Ultra™ RNA Library Prep Kit for Illumina^®^ (NEB, USA) according to the manufacturer's instructions and sequenced using the Illumina HiSeq X Ten platform. The RNA-seq reads were either *de novo* assembled using Trinity or mapped to the Manado pipefish (*M. manadensis*) genome using TopHat v2.0.12 [50] with default parameters, and were subsequently analyzed using in-house Perl scripts. Differential expression of genes at different stages of brood pouch development was performed using a previously developed method [51].

### Genome annotation

Annotation of the Manado pipefish (*M. manadensis*) genome was carried out using the Ensembl gene annotation pipeline, which integrated *Abinitio* gene predictions and evidence-based gene models (see Supplementary Fig. S4.2).

*Ab initio* prediction: The genome sequence of the Manado pipefish was repeat-masked and 34 585 genes from the homology gene sets were used to train the model parameters for Augustus. Subsequently, we used GENSCAN [52], Augustus [53], GlimmerHMM [54], GeneID [55] and SNAP [56] for *de novo* gene prediction using the repeat-masked genome sequence.

Homology-based gene prediction: We aligned *Xiphophorus maculatus*, *Cyprinus carpio*, *Gasterosteus aculeatus*, *H. erectus* and *H. comes* proteins to the pipefish genome using TblastN [57] with parameter E-value ≤ 1E-5, and then made use of GeMoMa [58] for precise spliced alignment and prediction of gene structures.

Transcriptome-based prediction: RNA-Seq reads were assembled *de novo* into contigs using Trinity and the resulting unigenes were aligned to the repeat-masked assembly using BLAT. Subsequently, the gene structures from the BLAT alignment results were modeled using PASA [59].

Finally, 21 003 consensus gene models were generated by integrating the *Abinitio* predictions, protein alignments and transcript data using EVidenceModeler. We annotated the predicted gene models using Nr, Nt, TrEMBL, GO, KOG and KEGG databases.

### Phylogenetic tree construction

#### Ortholog identification and extraction

To determine the phylogenetic position of the Manado pipefish (*M. manadensis*, family Syngnathidae, sub-family Nerophinae) within the other syngnathids, we performed phylogenomic analyses using orthologues from other ray-finned fish species. We used the Ensembl BioMart (www.ensembl.org/biomart; Ensembl version 89) to extract orthologues for zebrafish *Danio rerio*, fugu *Takifugu rubripes*, stickleback *Gasterosteus aculeatus*, medaka *Oryzias latipes*, tilapia *Oreochromis niloticus* and spotted gar *Lepisosteus oculatus*. This six species orthologue dataset was filtered out to retain only high-confidence one-to-one orthologues. The resultant six species, one-to-one orthologue set contained 5281 genes. Sequences for other teleost species such as the Gulf pipefish *S. scovelli* [7], the tiger tail seahorse *H. comes* [5], the lined seahorse *H. erectus* [6] and the great blue-spotted mudskipper *Boleophthalmus pectinirostris* [60] were obtained from their respective genome websites. To extrapolate the Ensembl BioMart orthologues to other fish datasets, we used zebrafish as the reference to identify orthologues in the non-Ensembl fishes. We ran InParanoid version 4.1 [61] for the five species pairs (zebrafish-Manado pipefish, zebrafish-Gulf pipefish, zebrafish-tiger tail seahorse, zebrafish-lined seahorse and zebrafish-blue-spotted mudskipper) at default settings (i.e. minimum 50% alignment span, minimum 25% alignment coverage, minimum BLASTP score of 40 bits, minimum inparalog confidence level of 0.05) to identify orthologues between zebrafish and each of the five fishes. By comparing the five InParanoid outputs with a list of 5281 zebrafish genes from the BioMart dataset, we narrowed down the list of one-to-one orthologues present in all 11 species. This 11-species one-to-one orthologue dataset comprises 2634 genes.

#### Phylogenomic analyses using the genome-scale datasets

Multiple alignments were generated at the protein level for each of the 2634 one-to-one orthologues using ClustalW version 2.0.12 [62]. Coding sequence alignments were generated based on respective protein alignments using PAL2NAL version 14 [63]. Concatenated protein and coding sequence alignments were prepared for the 11-species alignments by merging the individual protein and coding sequence alignments. Alignment gaps and ambiguous positions were removed using Gblocks version 0.91b [64]. The best-suited substitution model for both alignments was deduced using ModelGenerator version 0.85 [65]. We used the Maximum Likelihood (ML) method for phylogenetic analyses using the best-fit substitution model as predicted by ModelGenerator (protein: JTT+I+G+F; coding nucleotide: GTR+I+G). RAxML version 8.1.3 PTHREADS-SSE3 [66] was used for the ML analysis. We used RAxML’s rapid bootstrapping algorithm plus a thorough ML search (-f a option) and 1000 bootstrap replicates for node support.

### Rate of molecular evolution

#### Tajima's relative rate test

We used the trimmed concatenated protein alignment (1 162 665 positions) obtained from the core ortholog dataset for the 11 ray-finned fish species for the analysis. Spotted gar was used as outgroup for testing the relative evolutionary rates of Manado pipefish (*M. manadensis*) proteins with those of other teleosts. Tajima's relative rate test (RRT) is based on three sequences – two ingroups and one outgroup. A significantly higher/lower number of unique differences (based on *P* value) in either of the ingroup sequences would suggest that the particular ingroup is evolving at a significantly faster/slower rate respectively, compared to the other ingroup. The RRT analysis was done using MEGA version 7.0.18 [67].

#### Neutral tree based on four-fold degenerate sites

To compare the neutral nucleotide mutation rate for the different species, we generated a neutral tree based on an alignment of 4-fold degenerate (4D) sites from the 11 ray-finned fishes. We used the topology obtained from our phylogenomic analyses (see Supplementary Fig. S5.2) as an input for RAxML-based optimization of branch lengths for the 4D alignment. Codon alignments of the coding sequences based on the individual protein alignments were generated using PAL2NAL version 14 [63]. A concatenated coding sequence alignment was generated from these. An alignment of 4D sites was extracted from this coding sequence alignment using the ‘do.4d’ option (‘read.msa’ function) as implemented in the RPHAST package [68]. The extracted 4D alignment contained 262 619 positions. We used the ‘-f e’ option (optimize model+branch lengths for the input tree under GAMMA) in RAxML-8.1.3 [66] to generate a neutral tree for this alignment. A General Time Reversible nucleotide substitution model [69] with Gamma model of rate heterogeneity and a proportion of invariant sites (GTRGAMMAI) as deduced by ModelGenerator [70] was used for the analysis. Pairwise distances to the outgroup (spotted gar) were calculated from the neutral tree using the ‘cophenetic.phylo’ function as implemented in the R-package ‘ape’ [71].

#### Convergent evolution analysis

To detect convergent signatures at a genome-wide level between syngnathids and placental mammals, we employed the ‘convergence at conservative sites’ approach [12]. A total of 2912 orthologous gene sets was obtained from four syngnathids (tiger-tail seahorse, *H. comes*; lined seahorse, *H. erectus*; Gulf pipefish, *S. scovelli*; Manado pipefish, *M. manadensis*), four placental mammals (human, *Homo sapiens*; orangutan, *Pongo abelii*; mouse, *Mus musculus*; dog, *Canis familiaris*), four aplacental tetrapods (chicken, *Gallus gallus*; anole lizard, *Anolis carolinensis*; zebra finch, *Taeniopygia guttata*; turkey, *Meleagris gallopavo*), four aplacental teleosts (medaka, *O. latipes*; platyfish, *X. maculatus*; Nile tilapia, *O. niloticus*; stickleback, *G. aculeatus*) and a cartilaginous fish, the elephant shark (*Callorhinchus milii*) based on the syntenic pairwise alignments and reciprocal best alignments. Amino acid alignments were generated using MUSCLE v3.8.31 [72]. Maximum likelihood phylogenetic trees were generated using RAxML v8.2.4 [66]. To detect convergent signatures at a genome-wide level between syngnathid species and mammals, we used a previously published symmetric design method [12]. The designed phylogenetic topology consists of syngnathid species and mammals as the foreground convergent clades, each of which contains four species along with an equal number of aplacental species (controls).

The amino acid frequency, branch length and the best shape parameter for variable rates among sites (alpha) were obtained from the amino acid sequences described above using the *codeml* program from PAML [73]. The JTT+gamma model was employed as the amino acid substitution model. Using these parameters, amino acid sequence simulation was performed using the program *evolver* from PAML [73]. This initially generates a random ancestral sequence as per the given amino acid frequency, which is followed by sequence evolution according to the given tree topology and branch lengths. Finally, the simulated amino acid state at each internal node and leaf was obtained. We simulated the same number of amino acids as found in the real dataset to simplify comparisons. Based on the CCS method, the character in the outgroup was assumed to be the ancestral state. We further estimated the accuracy of the inferred ancestral character. Using the simulated amino acids at the leaves under the same criteria as the CCS method, the conservative sites of simulated sequences in the outgroup were selected. The simulated state in the outgroup was compared with the simulated state in the ancestral nodes to get the accuracy of the inference.

## Supplementary Material

nwaa002_Supplemental_File

## Data Availability

The Manado pipefish (*M. manadensis*) whole-genome sequence has been deposited in the DDBJ/EMBL/GenBank database under accession number QODF00000000. RNA-seq reads for *M. manadensis* have been deposited in the NCBI Sequence Read Archive under the accession number SRP156240.
