# Supplementary material for: Comparative genomics reveal shared genomic changes in syngnathid fishes and signatures of genetic convergence with placental mammals
Source: Natl Sci Rev. 2020 Jan 9;7(6):964–77. doi: 10.1093/nsr/nwaa002 (PMC8289055; doi:10.1093/nsr/nwaa002)
Supplement: nwaa002_Supplemental_File [file nwaa002_supplemental_file.docx]

**Supplementary Data for**

**Comparative genomics reveal** **shared genomic changes in syngnathid fishes and** **signatures of genetic convergence with placental mammals**

Yanhong Zhang^1†^, Vydianathan Ravi^2†^, Geng Qin^1,3^, He Dai^4^, Huixian Zhang^1^, Fengming Han^4^, Xin Wang^1^, Yuhong Liu^1^, Jianping Yin^1,3^, Liangmin Huang^1,5^, Byrappa Venkatesh^2*^, Qiang Lin^1,3, 5*^

^1^CAS Key Laboratory of Tropical Marine Bio-resources and Ecology, South China Sea Institute of Oceanology, Institute of South China Sea Ecology and Environmental Engineering, Chinese Academy of Sciences, Guangzhou 510301, China

^2^Comparative and Medical Genomics Laboratory, Institute of Molecular and Cell Biology, A*STAR 138673, Singapore

^3^Southern Marine Science and Engineering Guangdong Laboratory (Guangzhou), Guangzhou 511458, China

^4^Biomarker Technologies Corporation, Beijing 101300, China

^5^University of Chinese Academy of Science, Beijing 100049, China

^†^These authors contributed equally to this work.

^*^Corresponding author. Email: [linqiang@scsio.ac.cn](mailto:linqiang@scsio.ac.cn) (Q.L.); [mcbbv@imcb.a-star.edu.sg](mailto:mcbbv@imcb.a-star.edu.sg) (B.V.)

[1. Introduction 1](#_Toc26815255)

[2. Genome sequencing and assembly 2](#_Toc26815256)

[2.1. DNA sample preparation and sequencing 2](#_Toc26815257)

[2.2. Quality control of raw sequencing reads 3](#_Toc26815258)

[2.3. Estimation of the Genome Size using a K-mer spectrum 4](#_Toc26815259)

[2.4. Genome assembly 4](#_Toc26815260)

[2.5. Quality control of the *M. manadensis* genome assembly 5](#_Toc26815261)

[2.6. GC-content of the *M. manadensis* genome 6](#_Toc26815262)

[3. RNA sequencing 8](#_Toc26815263)

[3.1. Library preparation for transcriptome sequencing 8](#_Toc26815264)

[3.2. Clustering and sequencing 9](#_Toc26815265)

[3.3. Data quality control 9](#_Toc26815266)

[3.4. Mapping of reads to the reference genome 9](#_Toc26815267)

[3.5. Quantification of gene expression level 9](#_Toc26815268)

[4. Genome analyses 11](#_Toc26815269)

[4.1. Transposable element analysis 11](#_Toc26815270)

[4.2. Gene prediction and annotation 17](#_Toc26815271)

[4.3. Non-coding RNA genes 19](#_Toc26815272)

[5. Gene family evolution 20](#_Toc26815273)

[5.1. Gene family analysis 20](#_Toc26815274)

[5.2 Phylogenetic tree construction 21](#_Toc26815275)

[5.3 Rate of molecular evolution 23](#_Toc26815276)

[5.4 Expansion and contraction of gene families 26](#_Toc26815277)

[6. Expansion of the protocadherin gene family 53](#_Toc26815278)

[7. The SCPP gene family in the Manado pipefish 60](#_Toc26815279)

[8. Convergent evolution 61](#_Toc26815280)

[References 70](#_Toc26815281)

# 1. Introduction

The family Syngnathidae (seahorses, pipefishes, pipehorses and seadragons) is a morphologically unique group within teleosts, comprising 57 genera and 319 species (Eschmeyer's Catalog of Fishes, [calacademy.org/scientists/projects/catalog-of-fishes](file:///C:\Users\raviv\Documents\BV_LAB_DATA\Pipefish\NSR\calacademy.org\scientists\projects\catalog-of-fishes)). In the traditional classification scheme based on morphological features, the syngnathids (Family Syngnathidae) are divided into two subfamilies: Hippocampinae (seahorses) comprising only one genus - *Hippocampus*, and Syngnathinae (pipefishes, pipehorses and seadragons), composed of all the remaining genera [[1](#_ENREF_1)]. However, a recent study based on eight molecular (nuclear and mitochondrial) markers and 17 morphological characters revealed a distinct split between trunk- and tail-brooding lineages (i.e. differing in the placement of the brood pouch). This study proposed the classification of Syngnathidae into two sub-families: Nerophinae (trunk-brooding pipefishes) and Syngnathinae (tail-brooders comprising seadragons, pipefishes, seahorses and pygmy pipehorses) [[2](#_ENREF_2)] (Supplementary Figure 1.1). This classification scheme implies multiple independent origins of the complex male brooding structure within the Family Syngnathidae. If this classification is indeed correct, it makes the subfamily Nerophinae a valuable outgroup to study the evolution of phenotypic diversity in Syngnathinae members such as seahorses, pipefishes, seadragons and pygmy pipehorses. In this study, we have sequenced the genome of a Nerophinae member, the Manado pipefish, *Microphis manadensis*, and compared it with the genomes of the three Syngnathinae members as well as other representative teleosts.


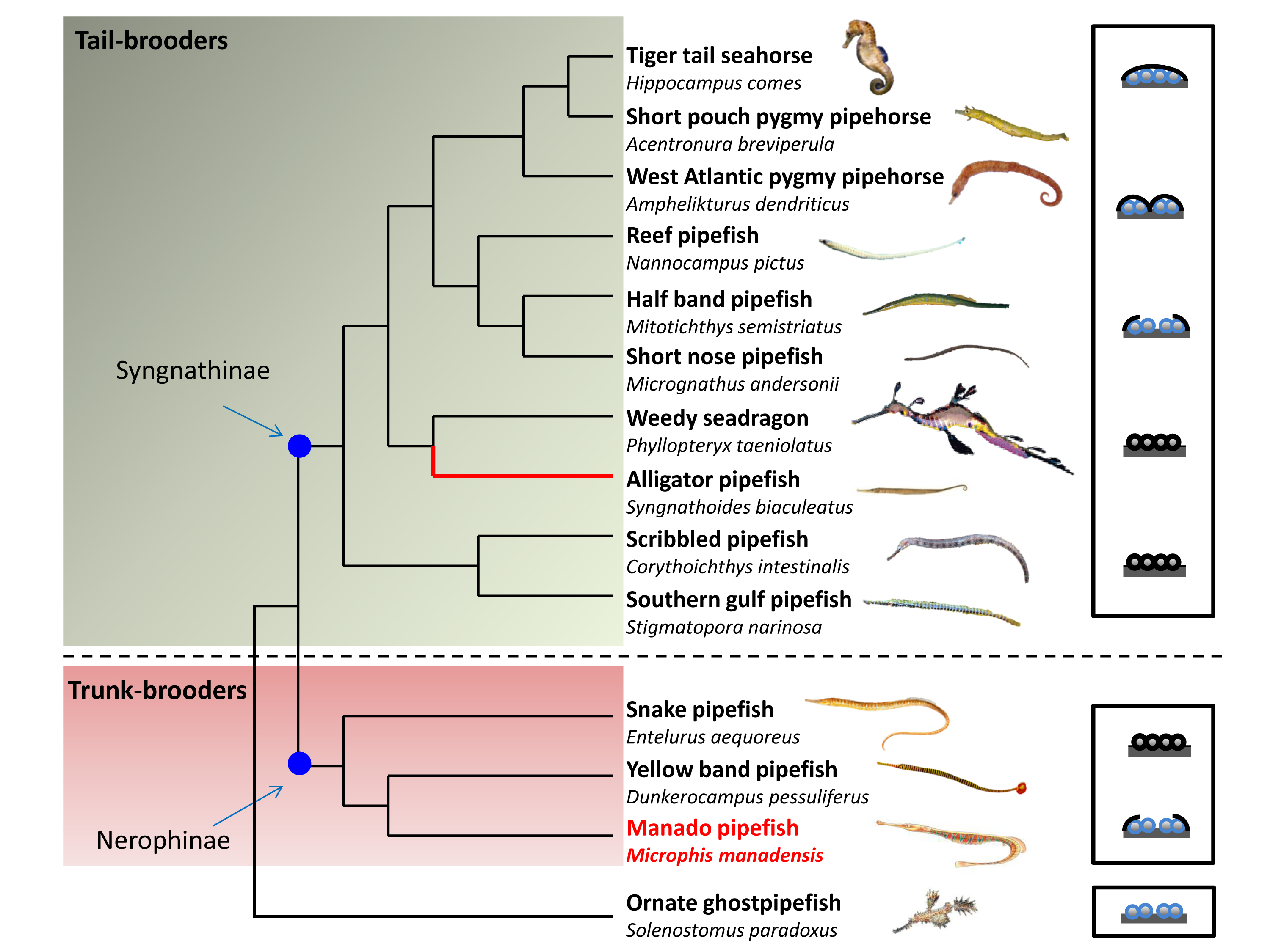


**Supplementary Figure 1.1** Phylogenetic relationship of fishes within the family Syngnathidae. A schematic of the brood pouch structure is shown on the right for each syngnathid species. Alligator pipefish (red branch) is the only species within subfamily Syngnathinae whose brood pouch is in the trunk region. Molecular phylogeny however, places it among the tail-brooders.

# 2. Genome sequencing and assembly

# 2.1. DNA sample preparation and sequencing

Genomic DNA was extracted from a single male Manado pipefish (*M. manadensis*). We constructed fourteen libraries including short-insert (200 bp, 220 bp, 500 bp) and long-insert (3 kb, 4 kb, 8 kb, 10 kb, 15 kb, 17 kb) libraries based on the standard protocol provided by Illumina (San Diego, USA), and sequenced the libraries using the Illumina HiSeq 2500 sequencing platform. In total, we generated around 184.95 Gb of raw sequencing data (Supplementary Table 2.1).

**Supplementary Table 2.1** Details of sequencing data generated using different libraries.

| **Library** | **Data (bp)** | **Depth (×)** | **Q20 (%)** | **Q30 (%)** |
| --- | --- | --- | --- | --- |
| 200 bp | 32,045,193,684 | 46.74 | 94.23 | 87.97 |
| 220 bp | 25,748,595,310 | 37.56 | 98.06 | 93.27 |
| 500 bp | 6,230,931,840 | 11.12 | 96.86 | 88.91 |
| 3 kb_1 | 22,422,650,388 | 32.71 | 92.19 | 86.35 |
| 3 kb_2 | 19,552,886,032 | 28.52 | 92.12 | 86.23 |
| 4 kb_1 | 16,183,447,706 | 23.61 | 91.87 | 85.79 |
| 4 kb_2 | 16,900,959,473 | 24.65 | 91.92 | 85.89 |
| 8 kb | 10,640,535,877 | 15.52 | 93.93 | 85.04 |
| 10 kb_1 | 2,978,376,512 | 4.34 | 95.88 | 87.56 |
| 10 kb_2 | 3,515,952,271 | 5.13 | 91.87 | 85.11 |
| 15 kb_1 | 6,492,952,634 | 9.47 | 91.89 | 85.18 |
| 15 kb_2 | 8,252,679,334 | 12.04 | 97.37 | 91.27 |
| 17 kb_1 | 4,837,048,457 | 7.06 | 96.69 | 89.96 |
| 17 kb_2 | 9,143,267,012 | 13.34 | 97.50 | 91.68 |
| Total | 184,945,476,530 | 271.81 | --- | --- |

# 2.2. Quality control of raw sequencing reads

In order to prepare high-quality data for *de novo* genome assembly, the raw sequencing data were filtered using the following strategy:

1. Reads from short-insert libraries were trimmed of four low-quality bases at both ends, while reads from long-insert libraries were trimmed of three low-quality bases;
2. For long-insert libraries, duplicated reads were filtered out;
3. Reads with 10 or more Ns were discarded;
4. Reads with more than 10 bp aligning to adapter sequences were filtered out.

Finally, 102.02 Gb of clean reads were obtained for the Manado pipefish genome assembly and size estimation.

# 2.3. Estimation of the Genome Size using a K-mer spectrum

The genome size of the Manado pipefish was estimated based on a K-mer spectrum [[3](#_ENREF_3)]. Given that the K-mer frequency obeys a Poisson distribution, when the coverage is sufficient, the genome size can be estimated by:

Genome Size = K*_num_*/K*_depth_*

Where K*_num_* is the number of K-mers, and K*_depth_* is the expected depth of K-mers. In this study, the K*_num_* is 20,567,700,000 based on 17-mers and K*_depth_* is 30 (Supplementary Figure 2.1). Therefore, the estimated genome size of the Manado pipefish is around 685.59 Mb.


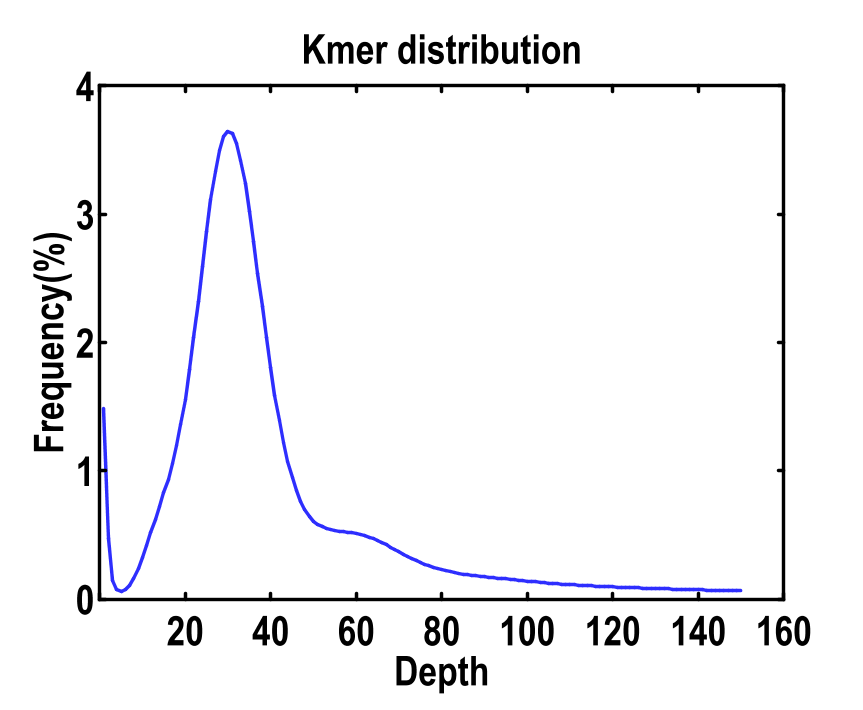


**Supplementary Figure 2.1** Genome size and sequence depth estimation based on a K-mer spectrum. The K-mer spectrum was constructed based on a 17-mer. The figure shows the K-mer spectrum of raw reads. The x-axis represents the K-mer depth whereas the y-axis denotes the proportion of K-mers. The peak of the distribution is 30.

# 2.4. Genome assembly

The Manado pipefish genome was assembled using ALLPATHS-LG [[4](#_ENREF_4)] with default parameters. Specifically, we first constructed contigs using filtered paired-end reads (200 bp, 220 bp, 500 bp). The mate-pair reads (3 kb, 4 kb, 8 kb, 10 kb, 15 kb, 17 kb) were used to bridge the contigs with SSPACE [[5](#_ENREF_5)]. Finally, assembly gaps were filled using reads from the paired-end libraries using GapCloser1.10 [[6](#_ENREF_6)]. The resulting pipefish assembly spans approximately 653.44 Mb (Supplementary Table 2.2).

**Supplementary Table 2.2** Summary of the Manado pipefish genome assembly.

|  | **Scaffold** | | **Contig** | |
| --- | --- | --- | --- | --- |
|  | **Size (bp)** | **Number** | **Size (bp)** | **Number** |
| N50 | 2,754,015 | --- | 67,986 | --- |
| N90 | 763,877 | --- | 17,564 | --- |
| Longest | 9,411,435 | --- | 453,056 | --- |
| Total Size (≥1 kb) | 653,443,195 | --- | 638,131,170* | --- |
| Total Number (≥1 kb) | --- | 2,059 | --- | 19,712* |
| *Contigs within scaffolds ≥1 kb | | | | |

# 2.5. Quality control of the *M. manadensis* genome assembly

The quality of the assembled pipefish genome was assessed using CEGMA (Core Eukaryotic Gene Mapping Approach), BUSCO (Benchmarking Universal Single Copy Orthologs) and the assembled transcriptome data. CEGMA evaluates the completeness of a genome assembly using a set of genes that are widely conserved in eukaryotic genomes. In total, 454 core eukaryotic genes (CEGs), which are highly conserved, were mapped against the pipefish genome assembly. The analysis revealed that the pipefish assembly covered more than 99.19% (246/248) of the complete CEGs and more than 99.13% (454/458) with partial coverage. Furthermore, BUSCO analysis showed that 4,227 single-copy orthologues were complete in the genome assembly (Supplementary Table 2.3).

**Supplementary Table 2.3** BUSCO assessment results

| Complete BUSCOs (C) | 4,376 (95.46%) |
| --- | --- |
| Complete and single-copy BUSCOs (S) | 4,227 (92.21%) |
| Complete and duplicated BUSCOs (D) | 149 (3.25%) |
| Fragmented BUSCOs (F) | 70 (1.53%) |
| Missing BUSCOs (M) | 138 (3.01%) |
| Total Lineage BUSCOs | 4,584 |

The RNA-seq data (details given in Supplementary Table 2.4) generated in this study were assembled and the assembled fragments were mapped to the genome assembly using BLAT with default parameters and an identity cutoff of 90%. This revealed that more than 90% of the transcribed regions (>200 bp) were covered in the pipefish assembly.

**Supplementary Table 2.4** Assessment of the completeness of coding regions using transcriptome data in the Manado pipefish genome.

| Dataset | Number | Total Length (bp) | Base covered by Assembly | Sequence covered by Assembly | With >90% sequence  in one Scaffold | | With >50% Sequence  in one Scaffold | |
| --- | --- | --- | --- | --- | --- | --- | --- | --- |
|  |  |  |  |  | **Number** | **Percent** | **Number** | **Percent** |
| All | 97,241 | 66,453,477 | 90.95% | 90.22% | 88,983 | 82.56% | 96,671 | 89.69% |
| >200 bp | 97,241 | 66,453,477 | 90.95% | 90.22% | 96,671 | 89.69% | 96,671 | 89.69% |
| >500 bp | 29,760 | 47,736,160 | 92.91% | 94.88% | 26,854 | 85.61% | 29,615 | 94.42% |
| >1000 bp | 16,429 | 38,844,554 | 93.86% | 97.37% | 14,741 | 87.36% | 16,374 | 97.04% |

# 2.6. GC-content of the *M. manadensis* genome

The GC content of the Manado pipefish genome was estimated using a sliding window approach. Briefly, a 500 bp (250 bp stepwise) sliding window was employed to scan along the genome and calculate the GC content. We found that the average GC content of the Manado pipefish genome is about 43.34%, which is similar to the repeat content in other teleost genomes (Supplementary Figure 2.2).


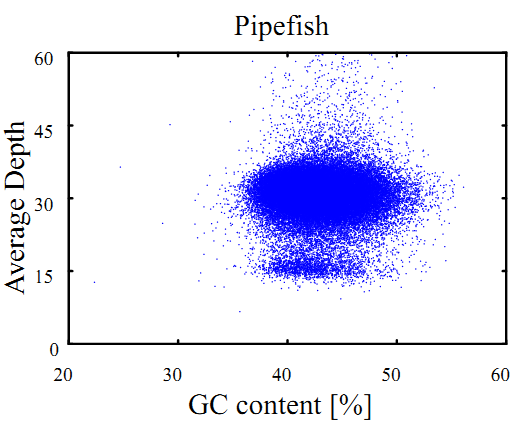


Supplementary Figure 2.2 GC content in teleost genomes. The x-axis represents GC content and the y-axis denotes the average depth.

# 3. RNA sequencing

Male Manado pipefishes of known reproductive stages (including pregnant and non-pregnant stage) were maintained under standard laboratory conditions. Animals were euthanized by decapitation according to approved protocols. The tissues were stored at -80°C until RNA extraction. In total, 21 RNA-seq libraries were constructed in this study, including one library of combined soft-tissues (brain, gills, intestine, liver, and muscle) from a male Manado pipefish and 20 libraries of different development stages (Supplementary Table 3.1).

# 3.1. Library preparation for transcriptome sequencing

A total of 3 µg RNA per sample was used as input material for library preparation. Sequencing libraries were generated using NEBNext® Ultra™ RNA Library Prep Kit for Illumina® (NEB, USA) following manufacturer’s recommendations, and index codes were added to associate sequences to each sample. Briefly, mRNA was purified from total RNA using poly-T oligo-attached magnetic beads. Fragmentation was carried out using divalent cations under elevated temperature in NEBNext First Strand Synthesis Reaction Buffer (5X). First strand cDNA was synthesized using random hexamer primer and M-MuLV Reverse Transcriptase (RNase H). Second strand cDNA synthesis was subsequently performed using DNA Polymerase I and RNase H. Remaining overhangs were converted into blunt ends via exonuclease/polymerase activities. After adenylation of 3’ ends of DNA fragments, NEBNext Adaptors with hairpin loop structure were ligated to prepare for hybridization. In order to select cDNA fragments of preferentially 150-200 bp in length, the library fragments were purified using the AMPure XP system (Beckman Coulter, Beverly, USA). Subsequently 3 µl USER^TM^ Enzyme (NEB, USA) was used with size-selected, adaptor-ligated cDNA at 37°C for 15 min followed by 5 min at 95 °C before PCR. The PCR was performed using Phusion High-Fidelity DNA polymerase, Universal PCR primers and Index (X) Primers. Finally PCR products were purified (AMPure XP system) and library quality was assessed on the Agilent Bioanalyzer 2100 system.

# 3.2. Clustering and sequencing

Clustering of the index-coded samples was performed on a cBot Cluster Generation System using the TruSeq PE Cluster Kit v3-cBot-HS (Illumina) according to the manufacturer’s instructions. After cluster generation, the library preparations were sequenced on an Illumina HiSeq X Ten platform and 125 bp/150 bp paired-end reads were generated.

# 3.3. Data quality control

Raw data (raw reads) in fastq format were firstly processed using in-house perl scripts. In this step, clean data (clean reads) were obtained by removing reads containing adapters, poly-Ns and low quality reads. Q20, Q30 and GC content was calculated for the clean data (Supplementary Table 3.1). All downstream analyses were based on the clean high quality data.

# 3.4. Mapping of reads to the reference genome

Reference genome and gene model annotation files were downloaded from the genome website directly. An index of the reference genome was built using Bowtie v2.2.3 and clean paired-end reads were aligned to the reference genome using TopHat v2.0.12 [[7](#_ENREF_7)]. We selected TopHat as the mapping tool as it can generate a database of splice junctions based on the gene model annotation file and can therefore produce a better mapping result than other non-splice mapping tools.

# 3.5. Quantification of gene expression level

HTSeq v0.6.1 was used to count the number of reads mapped to each gene. The FPKM (expected number of Fragments Per Kilobase of transcript sequence per Million base pairs sequenced) for each gene was calculated based on the length of the gene and the number of reads mapped to it. FPKM considers the effect of sequencing depth and gene length for the read count at the same time, and is currently the most commonly used method for estimating gene expression levels [[8](#_ENREF_8)].

**Supplementary Table 3.1** Transcriptome sequencing data statistics

| Sample name | Raw reads | Clean reads | Clean bases (Gb) | Error rate (%) | Q20 (%) | Q30 (%) | GC content (%) |
| --- | --- | --- | --- | --- | --- | --- | --- |
| npm_br_1 | 63,291,540 | 57,879,280 | 8.68 | 0.02 | 96.71 | 91.94 | 51.21 |
| npm_br_2 | 54,313,276 | 50,720,578 | 7.61 | 0.02 | 96.45 | 91.48 | 51.65 |
| npm_br_3 | 65,698,682 | 60,765,780 | 9.11 | 0.02 | 96.57 | 91.64 | 50.92 |
| npm_br_4 | 61,604,014 | 56,442,598 | 8.47 | 0.02 | 96.55 | 91.56 | 51.79 |
| npm_te_1* | 54,670,368 | 51,528,890 | 7.73 | 0.02 | 96.59 | 91.67 | 50.58 |
| npm_te_2* | 59,835,380 | 54,448,810 | 8.17 | 0.02 | 96.91 | 92.34 | 51.04 |
| npm_bp_1 | 45,118,378 | 44,235,936 | 6.64 | 0.02 | 97.03 | 92.53 | 49.37 |
| npm_bp_2 | 60,062,980 | 58,793,972 | 8.82 | 0.02 | 97.09 | 92.66 | 48.96 |
| npm_bp_3 | 49,311,328 | 48,229,090 | 7.23 | 0.02 | 97.14 | 92.76 | 51.91 |
| npm_bp_4 | 47,793,322 | 46,774,934 | 7.02 | 0.02 | 97.14 | 92.75 | 49.09 |
| pm_br_1 | 60,378,662 | 56,501,920 | 8.48 | 0.02 | 96.41 | 91.36 | 52.17 |
| pm_br_2 | 60,170,022 | 56,011,594 | 8.40 | 0.02 | 96.62 | 91.87 | 51.58 |
| pm_br_3 | 63,520,980 | 59,401,456 | 8.91 | 0.02 | 96.58 | 91.69 | 50.95 |
| pm_br_4 | 60,408,638 | 56,596,432 | 8.49 | 0.02 | 96.42 | 91.35 | 49.66 |
| pm_te_1* | 55,439,634 | 51,801,820 | 7.77 | 0.02 | 96.67 | 91.83 | 51.83 |
| pm_te_2* | 60,759,488 | 56,277,742 | 8.44 | 0.02 | 97.07 | 92.82 | 52.36 |
| pm_bp_1 | 60,122,870 | 57,819,694 | 8.67 | 0.02 | 95.55 | 89.40 | 49.29 |
| pm_bp_2 | 49,449,644 | 47,610,504 | 7.14 | 0.02 | 95.71 | 89.66 | 48.83 |
| pm_bp_3 | 55,630,382 | 52,798,968 | 7.92 | 0.02 | 96.40 | 91.11 | 48.33 |
| pm_bp_4 | 56,020,544 | 53,822,498 | 8.07 | 0.02 | 95.77 | 89.89 | 48.69 |

*The male pipefish gonad transcriptome includes only two biological replicates as compared to other samples which have four biological replicates.

# 4. Genome analyses

# 4.1. Transposable element analysis

We constructed a transposable element (TE) library of the Manado pipefish genome using a combination of homology-based and *de novo* approaches.

1. We used two software packages, PILER-DF [[9](#_ENREF_9)] and RepeatScout [[10](#_ENREF_10)], to construct a *de novo* TE library for the Manado pipefish genome. We ran both the tools at default parameters, filtered out short (<100 bp) sequences and those containing gaps (Ns > 5%), then combined the results to obtain a consensus library.
2. LTR_Finder software [[11](#_ENREF_11)] was used to identify LTRs, which are specific to LTR retrotransposons as well as *Dictyostelium* intermediate repeat sequence (DIRS) elements. einverted software (EMBOSS), which is specific for DNA transposons, was used to detect terminal inverted repeats (TIRs).
3. RepeatMasker (version 3.3.0, http://www.repeatmasker.org/) and RepeatProteinMask were employed to identify TEs based on homology searches against the Repbase library (release 16.03) [[12](#_ENREF_12)] using the parameters “-nolow -no_is -norna -parallel 1” and “-noLowSimple –pvalue 1e-4”.
4. An ab initio TE library was constructed with RepeatModeler version 1.08 (http://www.repeatmasker.org/RepeatModeler.html) using the default parameters. RepeatModeler identifies repeat elements by integrating two repeat finding programs RECON [[13](#_ENREF_13)] and RepeatScout. Using the repeat library constructed by RepeatModeler, we estimated the repeat content of the Manado pipefish genome using RepeatMasker version 4.0.5 with the sensitive mode (-s) option. The TE expansion history was constructed by first recalculating the divergence of the identified TE copies in the genome with the corresponding consensus sequence in the TE library using Kimura distance [[14](#_ENREF_14)] and then estimating the percentage of TEs in the genome at difference divergence levels.

Both retrotransposons and DNA transposons were identified in a wide variety of families (Supplementary Table 4.2). Only a few vertebrate TE families, such as the Copia retrotransposons are absent from this genome. The LINE retrotransposons are mostly represented by RTE (20.23%). DNA transposons are mainly represented by TIR superfamilies which amount to more than 1,125,057 copies. Among LTR retrotransposons, Gypsy families are the most predominant. Furthermore, all of the LTR retrotransposons are more in Nerophinae than in Syngnathinae. Using Kimura distances, we estimated the relative age of the different TE families in the Manado pipefish genome (Fig. 2b).

**Tc1/Mariner evolutionary analysis**

Using known Tc1/Mariner transposase proteins, we blast searched the four syngnathid species using an *E*-value <1e-10 to identify transposase domains. Only protein sequences longer than 300 aa were selected for further analysis. The identified transposase sequences were aligned using MUSCLE and Tc1/Mariner phylogeny was generated using RAxML. The common characteristics of the Tc1/Mariner superfamily include terminal inverted repeats (TIRs), a DNA-binding domain (includes a single HTH motif) and a catalytic domain (DDE-aspartate/aspartate/glutamate or DDD-aspartate/aspartate/aspartate) [[15-17](#_ENREF_15)]. The clustering of the Tc1/Mariner sequences from the four syngnathids was defined based on the presence of DDE or DDD motifs within the catalytic domain. Phylogenetic clustering of Tc1/Mariner sequences were dated by calculating the average nucleotide divergence of individual copies to their ancestral consensus sequence (Ks) and estimating the substitutions per synonymous site per year (λ) following the formula: T=Ks/λ, where T represents the divergence time. The λ value was estimated based on the substitution rate of 2,673 orthologous gene pairs between Manado pipefish and gulf pipefish plus their divergence time (~75 MYA). The consensus sequences for a specific group were generated using the online Consensus Maker tool (https://www.hiv.lanl.gov/content/sequence/CONSENSUS/AdvCon.html). The Ks values were computed using KaKs_Calculator2.0 toolkit [[18](#_ENREF_18)].

**Supplementary Table 4.1** Comparison of syngnathid genome assemblies and their respective transposable element (TE) content.

|  | Manado pipefish |  | Gulf pipefish |  | Lined seahorse |  | Tiger tail seahorse |
| --- | --- | --- | --- | --- | --- | --- | --- |
| Estimated genome size (Mb) | 686 |  | 351 |  | 489 |  | 695 |
| Assembled genome size (Mb) | 653 |  | 307 |  | 458 |  | 502 |
| Scaffold N50 (Mb) | 2.75 |  | 0.64 |  | 1.97 |  | 1.87 |
| Contig N50 (kb) | 67.99 |  | 32.20 |  | 14.57 |  | 34.65 |
| TE total length (Mb) | 348.73 |  | 37.96 |  | 129.2 |  | 125.72 |
| TE content (%) | 53.37 |  | 12.36 |  | 28.23 |  | 26.12 |

**Supplementary Table 4.2** Different categories of transposable elements in *M. manadensis* and other syngnathid genomes.

|  |  | Manado pipefish | | |  | Gulf pipefish | | |  | Tiger tail seahorse | | |  | Lined seahorse | | |
| --- | --- | --- | --- | --- | --- | --- | --- | --- | --- | --- | --- | --- | --- | --- | --- | --- |
| Type |  | Number | Length (bp) | Rate (%) |  | Number | Length (bp) | Rate (%) |  | Number | Length (bp) | Rate (%) |  | Number | Length (bp) | Rate (%) |
| ClassI/DIRS/DIRS |  | 15,420 | 3,336,830 | 0.51 |  | 594 | 50,602 | 0.02 |  | 13,842 | 2,271,163 | 0.47 |  | 6,097 | 1,250,320 | 0.27 |
| ClassI/DIRS/Ngaro |  | 23 | 1,447 | 0.00 |  | 11 | 786 | 0 |  | 31 | 1,957 | 0 |  | 27 | 2,059 | 0 |
| ClassI/PLE/LARD |  | 342,267 | 60,965,202 | 9.33 |  | 37,988 | 6,670,054 | 2.17 |  | 149,753 | 22,869,677 | 4.75 |  | 303,415 | 48,025,926 | 10.49 |
| ClassI/PLE/Penelope |  | 30,151 | 5,492,291 | 0.84 |  | 730 | 115,864 | 0.04 |  | 3,961 | 667,724 | 0.14 |  | 1,473 | 238,441 | 0.05 |
| ClassI/LINE/LINE |  | 55 | 10,801 | 0.00 |  | 372 | 156,541 | 0.05 |  | 4,317 | 591,979 | 0.12 |  | 3,863 | 640,606 | 0.14 |
| ClassI/LINE/I |  | 2,858 | 804,113 | 0.12 |  | 667 | 54,540 | 0.02 |  | 1,355 | 156,935 | 0.03 |  | 776 | 105,538 | 0.02 |
| ClassI/LINE/Jockey |  | 159,535 | 33,792,703 | 5.17 |  | 26,234 | 5,616,076 | 1.83 |  | 86,534 | 15,645,069 | 3.25 |  | 85,830 | 15,344,384 | 3.35 |
| ClassI/LINE/L1 |  | 10,186 | 2,324,589 | 0.36 |  | 1,615 | 142,788 | 0.05 |  | 2,706 | 415,490 | 0.09 |  | 3,103 | 362,740 | 0.08 |
| ClassI/LINE/R2 |  | 7,673 | 1,391,784 | 0.21 |  | 3,540 | 729,747 | 0.24 |  | 14,828 | 2,988,328 | 0.62 |  | 32,602 | 5,295,720 | 1.16 |
| ClassI/LINE/RTE |  | 627,337 | 132,164,757 | 20.23 |  | 13,153 | 3,844,600 | 1.25 |  | 55,294 | 9,154,911 | 1.9 |  | 52,352 | 9,338,043 | 2.04 |
| ClassI/LTR/LTR |  | 1,016 | 535,372 | 0.08 |  | 1,131 | 120,599 | 0.04 |  | 574 | 62,645 | 0.01 |  | 604 | 77,947 | 0.02 |
| ClassI/LTR/Bel-Pao |  | 3,492 | 1,402,762 | 0.21 |  | 1,120 | 334,837 | 0.11 |  | 2,646 | 631,177 | 0.13 |  | 1,408 | 326,418 | 0.07 |
| ClassI/LTR/Copia |  | 3,694 | 728,049 | 0.11 |  | 413 | 122,226 | 0.04 |  | 1,481 | 396,981 | 0.08 |  | 1,550 | 292,723 | 0.06 |
| ClassI/LTR/ERV |  | 11,061 | 4,245,229 | 0.65 |  | 7,069 | 1,570,929 | 0.51 |  | 3,468 | 473,347 | 0.1 |  | 3,957 | 533,021 | 0.12 |
| ClassI/LTR/Gypsy |  | 39,653 | 13,046,554 | 2.00 |  | 6,236 | 1,427,778 | 0.46 |  | 11,158 | 2,383,067 | 0.5 |  | 16,711 | 3,288,568 | 0.72 |
| ClassI/LTR/Retrovirus |  | 90 | 6,067 | 0.00 |  | 106 | 7,212 | 0 |  | 191 | 12,665 | 0 |  | 159 | 9,582 | 0 |
| ClassI/SINE/5S |  | 60 | 5,627 | 0.00 |  | 36 | 2,886 | 0 |  | 24 | 2,148 | 0 |  | 20 | 1,520 | 0 |
| ClassI/SINE/7SL |  | 9 | 2,145 | 0.00 |  | 3 | 694 | 0 |  | 6 | 1,824 | 0 |  | 6 | 1,610 | 0 |
| ClassI/SINE |  | 1,579 | 267,456 | 0.04 |  | 772 | 134,141 | 0.04 |  | 883 | 124,363 | 0.03 |  | 1,200 | 186,915 | 0.04 |
| ClassI/SINE/Alu |  | 2 | 88 | 0.00 |  | 2 | 174 | 0 |  | 5 | 419 | 0 |  | 3 | 303 | 0 |
| ClassI/TRIM |  | 1,660 | 699,278 | 0.11 |  | 315 | 83,204 | 0.03 |  | 210 | 66,488 | 0.01 |  | 227 | 67,065 | 0.01 |
| ClassI/Sola |  | 421 | 41,870 | 0.01 |  | 632 | 47,321 | 0.02 |  | 509 | 52,253 | 0.01 |  | 651 | 96534 | 0.02 |
|  |  |  |  |  |  |  |  |  |  |  |  |  |  |  |  |  |
| ClassII/Academ |  | 187 | 13,283 | 0.00 |  | 187 | 12,185 | 0 |  | 222 | 14,791 | 0 |  | 196 | 12,857 | 0 |
| ClassII/Chapaev |  | 847 | 124,458 | 0.02 |  | 353 | 27,526 | 0.01 |  | 988 | 100,845 | 0.02 |  | 876 | 86,597 | 0.02 |
| ClassII/Ginger1 |  | 1,003 | 84,738 | 0.01 |  | 1,524 | 130,593 | 0.04 |  | 1,143 | 101,617 | 0.02 |  | 1,141 | 103,344 | 0.02 |
| ClassII/Ginger2 |  | 27 | 7,578 | 0.00 |  | 101 | 9,894 | 0 |  | 84 | 8,406 | 0 |  | 42 | 3,507 | 0 |
| ClassII/ISL2EU |  | 398 | 28,944 | 0.00 |  | 592 | 48,659 | 0.02 |  | 536 | 44,555 | 0.01 |  | 583 | 46,512 | 0.01 |
| ClassII/Kolobok |  | 800 | 64,796 | 0.01 |  | 928 | 72,382 | 0.02 |  | 1,008 | 81,159 | 0.02 |  | 1,008 | 85,569 | 0.02 |
| ClassII/Mirage |  | 0 | 0 | 0.00 |  | 0 | 0 | 0 |  | 0 | 0 | 0 |  | 2 | 81 | 0 |
| ClassII/MuDR |  | 395 | 61,916 | 0.01 |  | 153 | 12,423 | 0 |  | 234 | 17,266 | 0 |  | 446 | 63,146 | 0.01 |
| ClassII/Sola |  | 1,195 | 97,329 | 0.01 |  | 1,497 | 134,803 | 0.04 |  | 1,143 | 134,237 | 0.03 |  | 2,787 | 361,488 | 0.08 |
| ClassII/Zator |  | 10 | 514 | 0.00 |  | 9 | 493 | 0 |  | 15 | 771 | 0 |  | 20 | 3,564 | 0 |
| ClassII/Crypton |  | 290 | 25,857 | 0.00 |  | 236 | 18,558 | 0.01 |  | 510 | 48,217 | 0.01 |  | 2,060 | 257,215 | 0.06 |
| ClassII/Helitron |  | 0 | 0 | 0.00 |  | 0 | 0 | 0 |  | 1,402 | 238,519 | 0.05 |  | 876 | 119,211 | 0.03 |
| ClassII/Helitron | | 2,294 | 237,115 | 0.04 |  | 2,035 | 146,905 | 0.05 |  | 1,945 | 151,210 | 0.03 |  | 1,748 | 129,536 | 0.03 |
| ClassII/MITE |  | 9,744 | 1,656,292 | 0.25 |  | 952 | 125,032 | 0.04 |  | 3,430 | 635,714 | 0.13 |  | 3,067 | 498,860 | 0.11 |
| ClassII/Maverick |  | 3,411 | 731,207 | 0.11 |  | 604 | 96,442 | 0.03 |  | 3,930 | 443,022 | 0.09 |  | 2,144 | 290,520 | 0.06 |
| ClassII/Maverick |  | 1,089 | 81,290 | 0.01 |  | 2,148 | 303,411 | 0.1 |  | 1,667 | 121,465 | 0.03 |  | 1,451 | 103,919 | 0.02 |
| ClassII/TIR/TIR |  | 150 | 78,354 | 0.01 |  | 965 | 189,406 | 0.06 |  | 4,963 | 1,552,003 | 0.32 |  | 2,353 | 482,688 | 0.11 |
| ClassII/TIR/CACTA |  | 6,744 | 1,013,199 | 0.16 |  | 4,035 | 315,395 | 0.1 |  | 4,092 | 346,489 | 0.07 |  | 4,006 | 323,055 | 0.07 |
| ClassII/TIR/Merlin |  | 16 | 2,463 | 0.00 |  | 6 | 448 | 0 |  | 3 | 158 | 0 |  | 3 | 111 | 0 |
| ClassII/TIR/P |  | 992 | 111,041 | 0.02 |  | 266 | 19,644 | 0.01 |  | 635 | 47,263 | 0.01 |  | 686 | 57,196 | 0.01 |
| ClassII/TIR/PIF-Harbinger |  | 4,290 | 910,062 | 0.14 |  | 958 | 86,141 | 0.03 |  | 3,170 | 435,446 | 0.09 |  | 2,321 | 362,729 | 0.08 |
| ClassII/TIR/PiggyBac |  | 5,264 | 2,145,684 | 0.33 |  | 198 | 28,232 | 0.01 |  | 1,507 | 271,401 | 0.06 |  | 814 | 125,633 | 0.03 |
| ClassII/TIR/Tc1-Mariner |  | 1,048,348 | 215,978,478 | 33.05 |  | 56,194 | 10,598,316 | 3.45 |  | 284,244 | 47,949,721 | 9.96 |  | 257,396 | 44,097,438 | 9.63 |
| ClassII/TIR/Transib |  | 480 | 47,740 | 0.01 |  | 121 | 9,908 | 0 |  | 431 | 35,723 | 0.01 |  | 397 | 33,159 | 0.01 |
| ClassII/TIR/hAT |  | 58,773 | 11,115,607 | 1.70 |  | 22,527 | 4,146,361 | 1.35 |  | 108,412 | 18,761,009 | 3.9 |  | 96,295 | 16,847,225 | 3.68 |
| ClassII/TIR/Maverick |  | 0 | 0 | 0.00 |  | 0 | 0 | 0 |  | 0 | 0 | 0 |  | 1,719 | 287,176 | 0.06 |
|  |  |  |  |  |  |  |  |  |  |  |  |  |  |  |  |  |
| PotentialHostGene |  | 3,333 | 557,988 | 0.09 |  | 4,484 | 542,711 | 0.18 |  | 27,349 | 4,047,728 | 0.84 |  | 15,994 | 2,610,994 | 0.57 |
| SSR |  | 1,945 | 338,900 | 0.05 |  | 1,784 | 282,465 | 0.09 |  | 1,202 | 198,403 | 0.04 |  | 92 | 16,857 | 0 |
| Unknown |  | 60,007 | 10,522,597 | 1.61 |  | 36,936 | 6,703,161 | 2.18 |  | 205,830 | 29,179,512 | 6.06 |  | 113,079 | 16,555,547 | 3.62 |
| Total with overlap^1^: |  | 2,492,832 | 512,089,287 | 78.37 |  | 250,614 | 46,050,436 | 15 |  | 1,026,201 | 165,378,596 | 34.36 |  | 1,041,835 | 171,066,698 | 37.37 |
| Total without overlap^2^: |  | 2,492,832 | 348,735,124 | 53.37 |  | 250,614 | 37,968,725 | 12.36 |  | 1,026,201 | 125,729,413 | 26.12 |  | 1,041,835 | 129,205,739 | 28.23 |

^1^Total with overlap: total length of all repeats

^2^Total without overlap: total length of genome where repeats are located.

**
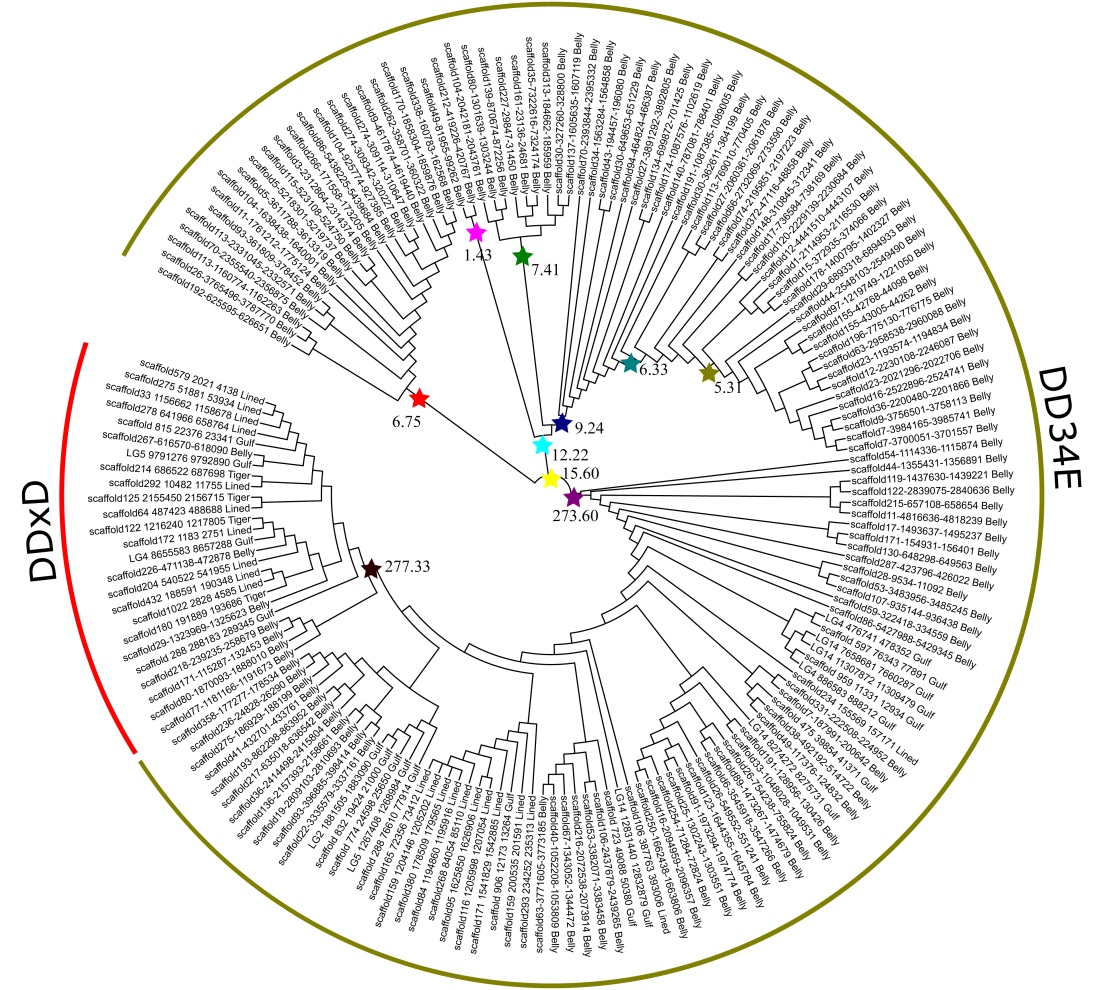
**

**Supplementary Figure 4.1** Phylogenetic relationship of Tc1/mariner transposons from syngnathid genomes. Abbreviation lists of taxon names: *Microphis manadensis*, Belly; *Syngnathus scovelli*, Gulf; *Hippocampus comes*, Tiger and *Hippocampus erectus*, Lined.

# 4.2. Gene prediction and annotation

**Supplementary Table 4.3** Summary of predicted gene models in the Manado pipefish genome.

| Method | Software | Species | Gene number |
| --- | --- | --- | --- |
| Ab initio | Genscan |  | 21,118 |
|  | Augustus |  | 22,367 |
|  | GlimmerHMM |  | 71,463 |
|  | GeneID |  | 23,298 |
|  | SNAP |  | 27,369 |
| Homology-based | GeMoMa | *Xiphophorus maculatus* | 14,227 |
|  |  | *Cyprinus carpio* | 36,480 |
|  |  | *Gasterosteus aculeatus* | 8,678 |
|  |  | *Hippocampus erectus* | 18,761 |
|  |  | *Hippocampus comes* | 12,982 |
| EST/Unigene | PASA |  | 15,244 |
| Integration | EVM |  | 21,003 |

**Supplementary Table 4.4** Details of gene structure for the Manado pipefish genes predicted using different methods as well as genes previously predicted in other teleost fishes.

| **Software** | **Gene**  **Num** | **Gene**  **Len (bp)** | **AveGen**  **Len (bp)** | **Exon**  **Len (bp)** | **AveExon**  **Len (bp)** | **Intron**  **Len (bp)** | **AveIntron**  **Len (bp)** |
| --- | --- | --- | --- | --- | --- | --- | --- |
| Genscan | 21,118 | 464,414,384 | 21,991.40 | 37,435,754 | 181.32 | 426,938,023 | 2,067.87 |
| Augustus | 22,367 | 284,288,218 | 12,710.16 | 34,127,886 | 160.64 | 250,160,332 | 1,177.53 |
| Glimmer | 71,463 | 449,989,860 | 6,296.82 | 42,019,297 | 156.95 | 407,970,557 | 1,523.81 |
| GeneID | 23,298 | 363,951,434 | 15,621.57 | 28,015,581 | 156.01 | 335,935,853 | 1,870.75 |
| SNAP | 27,369 | 237,017,737 | 8,660.08 | 22,501,466 | 166.49 | 213,887,726 | 1,582.54 |
| PASA | 15,244 | 98,257,726 | 6,445.67 | 18,039,736 | 168.86 | 79,832,639 | 747.27 |
| *Xiphophorus maculatus* | 14,227 | 145,753,027 | 10,244.82 | 22,490,407 | 155.72 | 115,157,046 | 797.35 |
| *Gasterosteus aculeatus* | 8,678 | 86,253,773 | 9,939.36 | 12,555,197 | 142.32 | 72,307,939 | 819.65 |
| *Cyprinus carpio* | 36,480 | 263,790,412 | 7,231.10 | 45,378,128 | 164.30 | 149,607,010 | 541.67 |
| *Hippocampus comes* | 12,982 | 108,374,951 | 8,348.09 | 17,279,000 | 176.13 | 85,686,502 | 873.43 |
| *Hippocampus erectus* | 18,761 | 236,040,442 | 12,581.44 | 33,383,306 | 171.46 | 191,604,609 | 984.09 |
| EVM | 21,003 | 273,450,474 | 13,019.59 | 35,916,481 | 166.03 | 224,758,072 | 1,038.99 |

**Supplementary Table 4.5** Number of gene models that could be annotated using different databases.

| **Annotation database** | **Annotated number** | **Percentage (%)** |
| --- | --- | --- |
| GO | 11,000 | 52.37 |
| KEGG | 12,656 | 60.26 |
| KOG | 14,911 | 70.99 |
| TrEMBL | 20,398 | 97.12 |
| Nr | 20,466 | 97.44 |
| Nt | 20,520 | 97.70 |
| All Annotated | 20,671 | 98.42 |


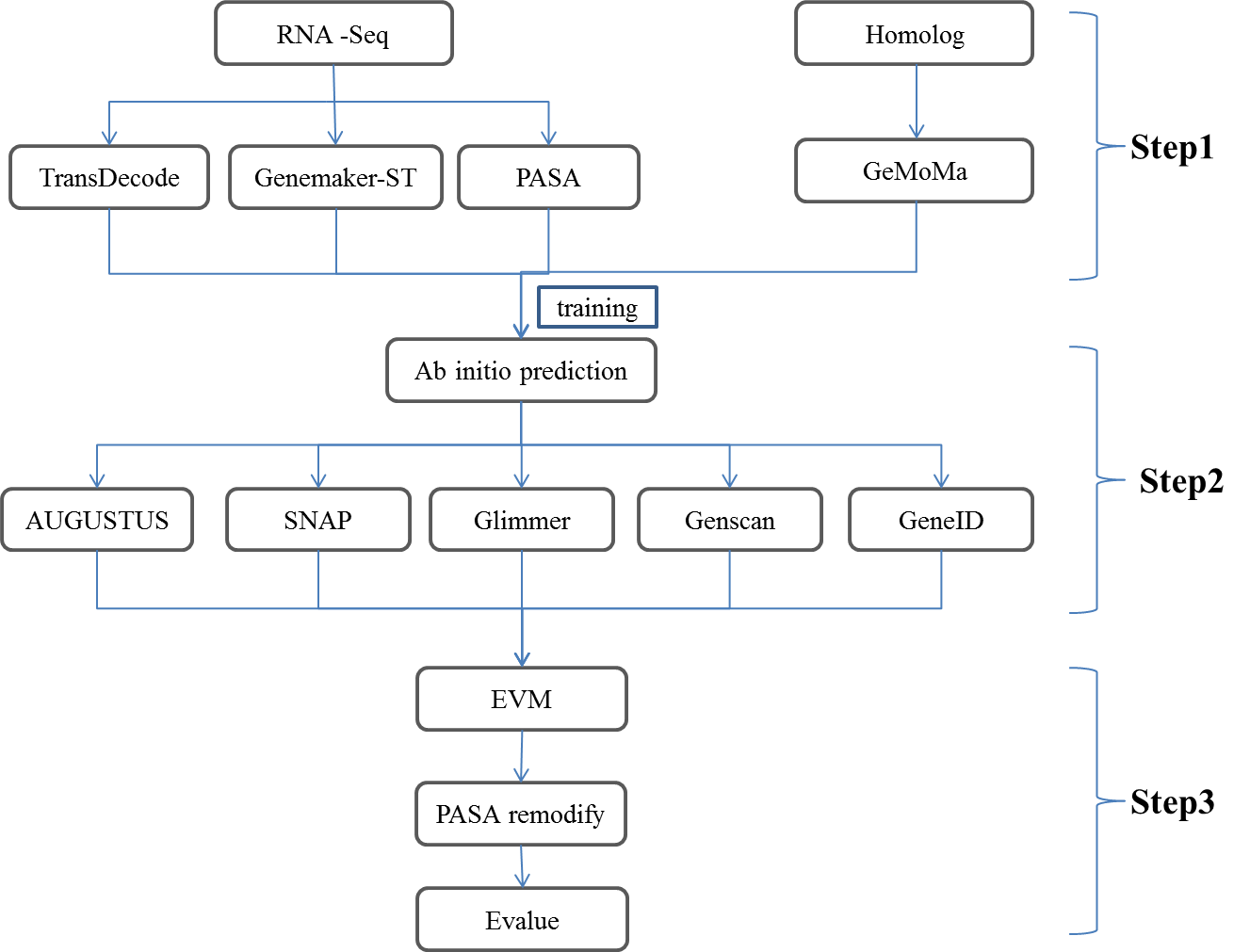


**Supplementary Figure 4.2** Flow diagram depicting the genome annotation pipeline used for the Manado pipefish genome.

# 4.3. Non-coding RNA genes

rRNA fragments were identified by aligning rRNA template sequences (Pfam database v31) using BLAST (E-value 1e-10 and identity cutoff ≥95%). The tRNAScan-SE algorithm at default parameters was used to predict tRNA genes. miRNA genes were predicted using INFERNAL v1.1 software against the Rfam database with a cutoff score of ≥30. Details of the non-coding RNAs predicted are given in Supplementary Table 4.6.

**Supplementary Table 4.6** Non-coding RNA genes in the Manado pipefish genome

| RNA classification | Number | Family |
| --- | --- | --- |
| miRNA | 219 | 78 |
| rRNA | 513 | 4 |
| tRNA | 986 | 25 |

# 5. Gene family evolution

# 5.1. Gene family analysis

Gene family analysis was conducted using Treefam as follows:

1. Protein sequences of six ray-finned fishes (zebrafish *Danio rerio*, stickleback *Gasterosteus aculeatus*, Nile tilapia *Oreochromis niloticus*, medaka *Oryzias latipes*, fugu *Takifugu rubripes*, and spotted gar *Lepisosteus oculatus*) were downloaded from the Ensembl database (Release 89) [[19](#_ENREF_19)]. Sequences for other teleost species such as the gulf pipefish *Syngnathus scovelli* [[20](#_ENREF_20)], the tiger tail seahorse *Hippocampus comes* [[21](#_ENREF_21)], the lined seahorse *Hippocampus erectus* [[22](#_ENREF_22)] and the great blue-spotted mudskipper *Boleophthalmus* *pectinirostris* [[23](#_ENREF_23)] were obtained from their respective genome websites.. BLASTP [[24](#_ENREF_24)] was employed to identify potential homologues using an E-value < 1e-10.
2. The raw Blast results were refined using solar (an in-house software, version 0.9.6) which enables the high-scoring segment pairs (HSPs) to be conjoined.
3. Similarity between protein sequences was evaluated using bit-score, and protein sequences were clustered into gene families using hcluster_sg, a hierarchical clustering algorithm in the Treefam pipeline (version 0.50) with the parameters: “-w 5 -s 0.33 -m 100,000”.

The gene family clustering result of the eleven species is shown in Supplementary Figure 5.1.


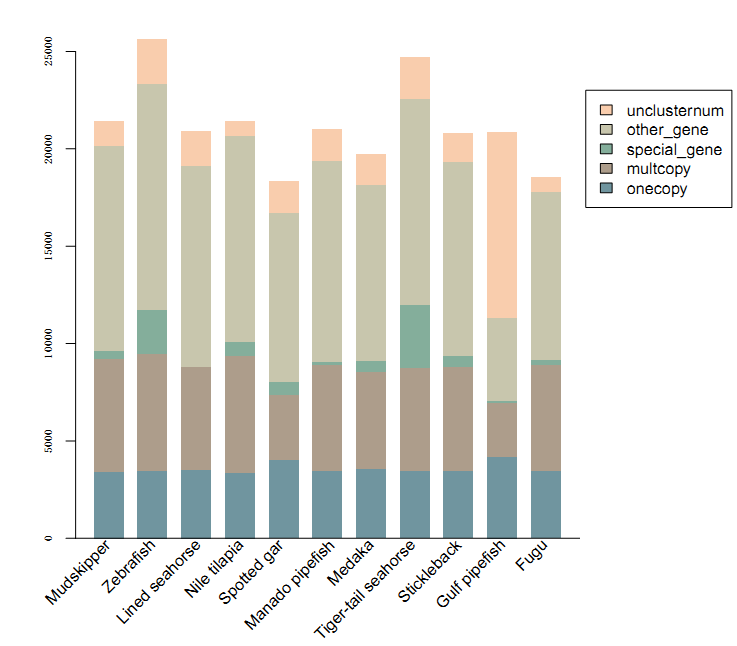


**Supplementary Figure 5.1** Gene family evolution in teleost fishes.

The x-axis represents the different teleost species analyzed whereas the y-axis denotes the counts for each gene family category.

Onecopy orthologs: species contain one copy each of the orthologous gene.

Multicopy orthologs: species contain multiple copies of the orthologous genes.

Special_gene: lineage-specific genes.

Other_gene: orthologous genes that could only be found in some species but not in all eleven species.

# 5.2 Phylogenetic tree construction

In order to verify the phylogenetic position of the Nerophinae group with respect to Syngnathinae, we used the genome-scale dataset of the Manado pipefish (a member of Nerophinae) along with datasets from other fishes (including Syngnathinae members) to perform phylogenomic analysis. Using a combination of Ensembl BioMart and InParanoid-based approaches, we were able to identify a set of 2,634 high-confidence, one-to-one orthologues present in each of the 11 selected fishes. Phylogenomic analyses using protein as well as coding nucleotide sequences gave identical topologies placing the Manado pipefish as an outgroup to the remaining syngnathids with maximal support (ML bootstrap 100%, Supplementary Figure 5.2). The tiger tail seahorse, lined seahorse and the Gulf pipefish, belonging to the sub-family Syngnathinae [[2](#_ENREF_2)], formed a monophyletic clade with the Gulf pipefish appearing sister to the two seahorses (ML bootstrap 100%). The Manado pipefish, belonging to the sub-family Nerophinae [[2](#_ENREF_2)], appeared as sister to the Syngnathinae clade (Supplementary Figure 5.2) confirming the results of Hamilton et al. (2017). Thus, the Manado pipefish can serve as a useful outgroup to understand the origin and evolution of the Syngnathinae members.


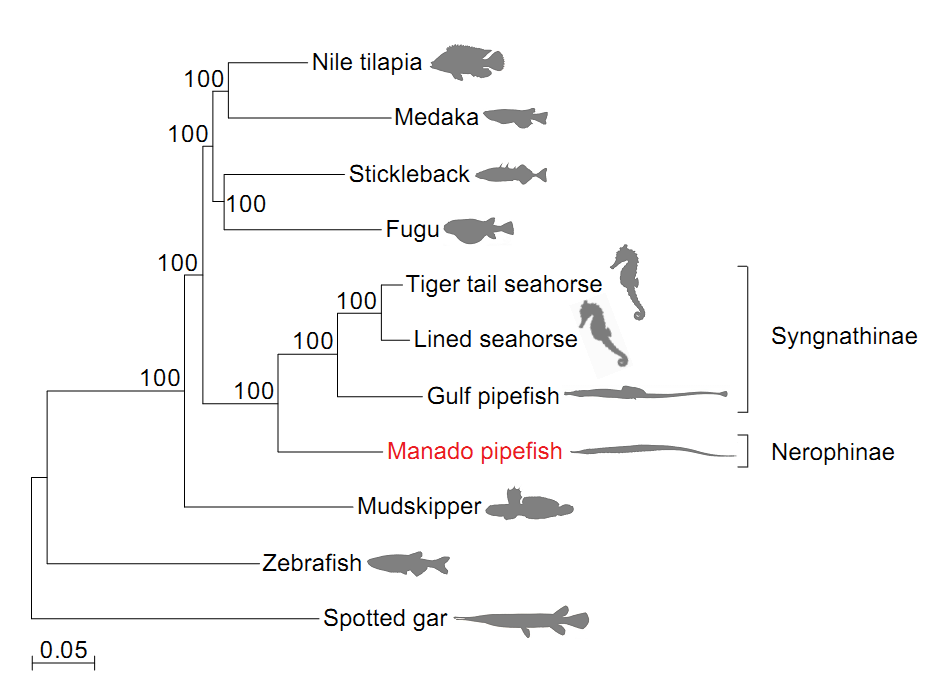


**Supplementary Figure 5.2** Phylogenomic position of Manado pipefish based on 2,634 one-to-one orthologues (1,162,665 amino acid positions) from 11 ray-finned fishes. An identical topology with similar bootstrap values was obtained using a coding nucleotide dataset (3,651,156 nucleotide positions) from the same 11 species (figure not shown). Values shown at the nodes are Maximum Likelihood bootstrap percentages. The scale bar represents 0.05 substitutions per site.

# 5.3 Rate of molecular evolution

Based on a genome-wide set of 4,122 orthologues, a previous study had shown that the genome of the tiger tail seahorse was evolving faster than other teleosts at the level of proteins as well as in terms of the neutral nucleotide mutation rate [[21](#_ENREF_21)]. In the current study, we have expanded the dataset to include additional syngnathids which include the lined seahorse and Gulf pipefish (Syngnathinae), and the Manado pipefish (Nerophinae). We tested the relative molecular evolutionary rates of proteins from the 11-fish dataset using the trimmed concatenated amino acid alignment. Spotted gar was used as the outgroup for comparing the molecular evolutionary rates of Manado pipefish proteins versus those of other teleosts. Tajima’s Relative Rate tests indicated that the Manado pipefish proteins were evolving significantly faster than all non-syngnathid teleosts (p-value < 0.01 for all comparisons; see Supplementary Table 5.1). Within syngnathids, the Manado pipefish proteins were the slowest-evolving whereas the Gulf pipefish possessed the fastest-evolving proteins (p-value < 0.01 in all cases; Supplementary Table 5.1). The two seahorses occupied an intermediate position within the syngnathids. Thus, the protein sequences of the Gulf pipefish seem to be evolving at a significantly faster rate than other syngnathids as well as compared to all other teleosts. As such, proteins from members of the Order Syngnathidae showed a higher evolutionary rate as compared to other teleosts, whereas members of the subfamily Syngnathinae showed a higher protein evolutionary rate than the Manado pipefish, a member of the subfamily Nerophinae.

To determine whether the higher evolutionary rate of protein sequences in Manado pipefish and other syngnathids is a reflection of their neutral nucleotide mutation rate, we generated a tree based on 4D sites. Based on the branch lengths of the neutral tree (Figure 1c), the Manado pipefish appears to be faster-evolving than all non-syngnathid teleosts (as was the case with the proteins). Within the syngnathids, the Gulf pipefish was the fastest-evolving, closely followed by the two seahorses and the Manado pipefish in terms of the branch length. To confirm this, we examined the actual distances to the outgroup (Supplementary Table 5.2) and found that the Gulf pipefish indeed possessed the greatest pairwise distance to the spotted gar (2.047 substitutions per 4D site) followed by the lined seahorse (2.025 substitutions per 4D site), the tiger tail seahorse (2.023 substitutions per 4D site) and the Manado pipefish (1.928 substitutions per 4D site). Thus, the Manado pipefish and the other syngnathids show a higher neutral evolutionary rate as well compared to other teleosts, and the Syngnathinae members show a higher neutral evolutionary rate as compared to Nerophinae. This may be related to the greater phenotypic diversity among members of the subfamily Syngnathinae which includes diverse fishes such as seadragons, pipefishes, seahorses and pygmy pipehorses, compared to Nerophinae which includes only pipefishes.

**Supplementary Table 5.1** Relative rate tests of the Manado pipefish versus the other teleosts using the 11-fish trimmed protein dataset and spotted gar as the outgroup. The ‘Fast-evolving’ column shows the ‘significantly’ faster-evolving ingroup species based on P-value. The ‘identical’ and ‘divergent’ columns refer to sites where the amino acid residue is the same or different in all three sequences, respectively. ‘Ingroup1-specific’column refers to sites where ingroup 2 and outgroup share the same amino acid but not ingroup 1. The same applies for ‘ingroup2-specific’ and ‘outgroup-specific’.

| **Ingroup1** | **Ingroup2** | **Outgroup** | **Genes** | **Identical** | **Divergent** | **Ingroup1**  **specific** | **Ingroup2**  **specific** | **Outgroup**  **specific** | **Fast-evolving** | **CHI^2**  **test statistic** | **P-value** |
| --- | --- | --- | --- | --- | --- | --- | --- | --- | --- | --- | --- |
| Zebrafish | Manado pipefish | Gar | 2,634 | 827,985 | 78,352 | 67,903 | 93,906 | 94,386 | Manado pipefish | 4,178.73 | 0 |
| Fugu | Manado pipefish | Gar | 2,634 | 841,272 | 63,509 | 54,627 | 57,326 | 145,815 | Manado pipefish | 65.07 | 7.23E-16 |
| Medaka | Manado pipefish | Gar | 2,634 | 839,454 | 64,306 | 56,403 | 58,746 | 143,595 | Manado pipefish | 47.67 | 5.03E-12 |
| Stickleback | Manado pipefish | Gar | 2,634 | 848,259 | 57,751 | 47,647 | 56,684 | 152,219 | Manado pipefish | 782.77 | 3.00E-172 |
| Nile tilapia | Manado pipefish | Gar | 2,634 | 859,782 | 53,891 | 36,119 | 58,690 | 154,072 | Manado pipefish | 5,373.43 | 0 |
| Gulf pipefish | Manado pipefish | Gar | 2,634 | 844,279 | 51,304 | 46,307 | 38,061 | 175,176 | Gulf pipefish | 805.95 | 2.74E-177 |
| Tiger tail seahorse | Manado pipefish | Gar | 2,634 | 855,038 | 48,693 | 40,842 | 37,917 | 180,033 | Tiger tail seahorse | 108.63 | 1.95E-25 |
| Lined seahorse | Manado pipefish | Gar | 2,634 | 852,259 | 50,015 | 43,649 | 38,178 | 178,463 | Lined seahorse | 365.79 | 1.54E-81 |
| Mudskipper | Manado pipefish | Gar | 2,634 | 838,909 | 66,194 | 55,757 | 59,002 | 141,109 | Manado pipefish | 91.76 | 9.80E-22 |

**Supplementary Table 5.2** Pairwise distance to the outgroup (spotted gar) for the 11-fish dataset. Pairwise distances were calculated from the 11-fish neutral tree using the R-package ‘ape’ (see Methods).

| **Species** | **Pairwise distance to spotted gar**  **(substitutions per 4D site)** |
| --- | --- |
| Nile tilapia | 1.75759 |
| Medaka | 1.952644 |
| Stickleback | 1.784764 |
| Fugu | 1.919865 |
| Tiger tail seahorse | 2.022724 |
| Lined seahorse | 2.025886 |
| Gulf pipefish | 2.047085 |
| Manado pipefish | 1.927811 |
| Blue-spotted mudskipper | 1.857307 |
| Zebrafish | 1.768424 |

# 5.4 Expansion and contraction of gene families

We analysed the expansion and contraction of gene families based on the birth-and-death model using CAFE (version2.1). Gene family results from the TreeFam pipeline and the estimated divergence time between species were used as inputs. We used the parameters "-p 0.01, -r 10000, -s" to search the birth and death parameter (λ) of genes, calculated the probability of each gene family with observed sizes using 10,000 Monte Carlo random samplings, and reported birth and death parameters in gene families with probability less than 0.01. For the gene family expansion and contraction analysis in Manado pipefish, we first filtered out the gene families without homology in the SWISS-PROT database [[25](#_ENREF_25)] to reduce the potential false positive expansions or contractions caused by gene prediction. In addition, families containing sequences that have multiple functional annotations were also removed. Finally, the Manado pipefish was found to possess 27 significantly expanded and 163 significantly contracted gene families compared to the common ancestor of syngnathids (see Supplementary Figure 5.3, Tables 5.3 and 5.4). The common ancestor of syngnathids was found to possess one significantly expanded and 154 significantly contracted gene families (Supplementary Figure 5.3 and Table 5.5).


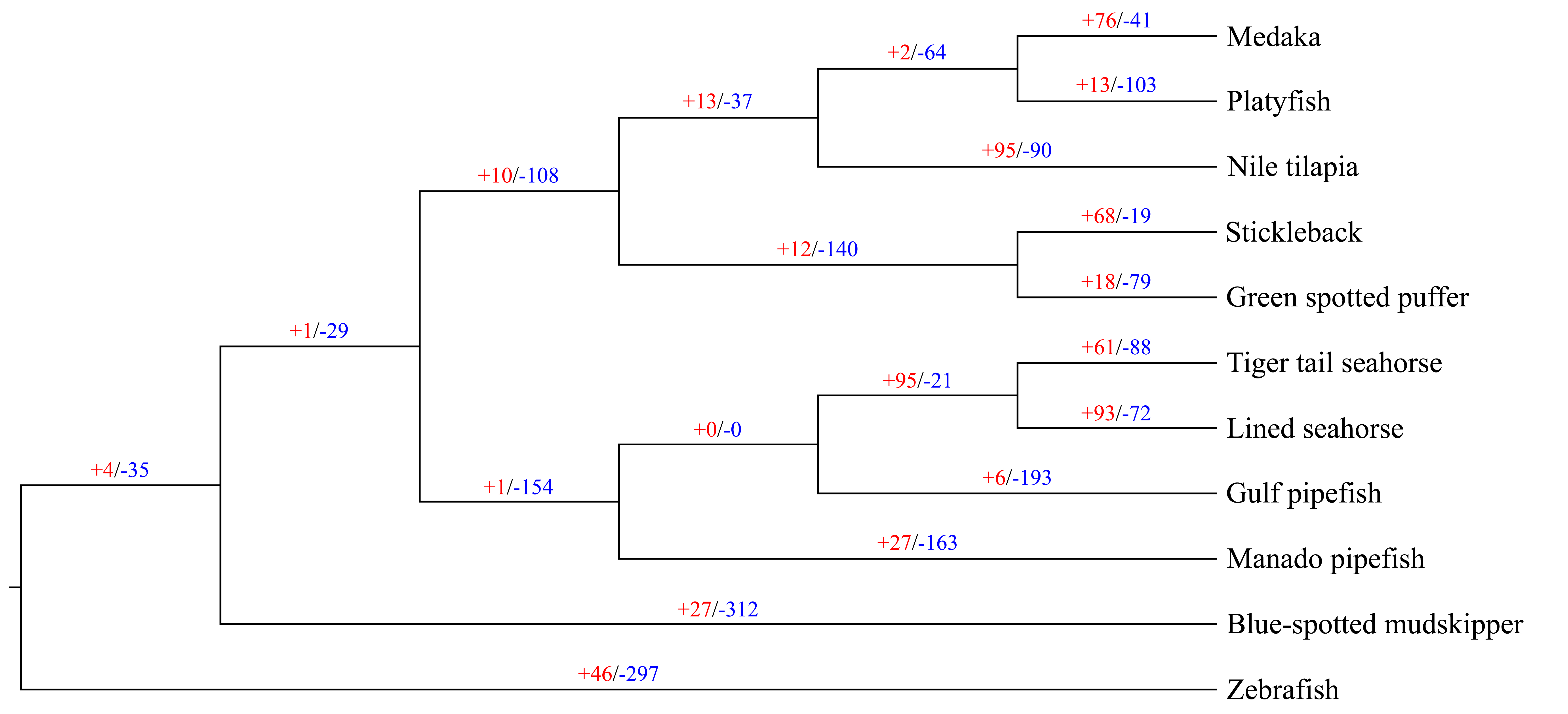


**Supplementary Figure 5.3** Expansion and contraction of gene families in the Manado pipefish genome. Expanded and contracted gene families are shown in red and blue, respectively.

**Supplementary Table 5.3** Expanded gene families in the Manado pipefish genome.

| Family_ID | Medaka | Platyfish | Nile tilapia | Stickleback | Green spotted puffer | Tiger tail seahorse | Lined seahorse | Gulf pipefish | Manado pipefish | Mudskipper | Zebrafish | Description |
| --- | --- | --- | --- | --- | --- | --- | --- | --- | --- | --- | --- | --- |
| GF_343 | 1 | 2 | 3 | 5 | 9 | 3 | 3 | 2 | 6 | 9 | 0 | Sodium:solute symporter family |
| GF_11 | 15 | 17 | 22 | 13 | 20 | 17 | 16 | 1 | 20 | 20 | 17 | Sodium:neurotransmitter symporter family |
| GF_1013 | 0 | 0 | 0 | 0 | 0 | 1 | 1 | 0 | 22 | 0 | 0 | Peptidase family M50 |
| GF_168 | 4 | 3 | 12 | 3 | 3 | 3 | 4 | 3 | 6 | 4 | 13 | A-macroglobulin complement component |
| GF_6938 | 2 | 0 | 0 | 1 | 0 | 4 | 0 | 0 | 2 | 1 | 0 | Pentapeptide repeats (9 copies) |
| GF_142 | 4 | 6 | 7 | 8 | 4 | 8 | 3 | 4 | 7 | 5 | 6 | C-terminal tandem repeated domain in type 4 procollagen |
| GF_848 | 7 | 3 | 6 | 3 | 0 | 1 | 2 | 1 | 5 | 1 | 0 | Zona pellucida-like domain |
| GF_6 | 26 | 18 | 24 | 22 | 23 | 21 | 22 | 2 | 22 | 22 | 26 | Myosin head (motor domain) |
| GF_2250 | 1 | 0 | 0 | 0 | 0 | 1 | 0 | 0 | 14 | 0 | 0 | E2F-associated phosphoprotein |
| GF_14 | 21 | 1 | 53 | 12 | 2 | 1 | 0 | 0 | 23 | 14 | 38 | Peptidase family M50 |
| GF_165 | 4 | 7 | 7 | 7 | 3 | 6 | 6 | 0 | 6 | 7 | 6 | G-protein alpha subunit |
| GF_2753 | 0 | 1 | 0 | 9 | 0 | 0 | 0 | 0 | 2 | 1 | 0 | Flavodoxin-like fold |
| GF_543 | 0 | 1 | 0 | 4 | 0 | 8 | 11 | 0 | 11 | 0 | 0 | Reverse transcriptase |
| GF_7135 | 0 | 0 | 0 | 0 | 0 | 0 | 0 | 0 | 10 | 0 | 0 | Activin types I and II receptor domain |
| GF_2254 | 3 | 1 | 1 | 0 | 0 | 6 | 1 | 0 | 2 | 0 | 2 | DDE superfamily endonuclease |
| GF_10551 | 0 | 0 | 0 | 0 | 0 | 0 | 0 | 0 | 9 | 0 | 0 | Peptidase family M50 |
| GF_5 | 18 | 22 | 24 | 23 | 18 | 22 | 27 | 22 | 58 | 66 | 74 | Cadherin domain |
| GF_3249 | 0 | 0 | 1 | 0 | 0 | 1 | 1 | 0 | 9 | 0 | 0 | Class I Histocompatibility antigen, domains alpha 1 and 2 |
| GF_1981 | 0 | 0 | 5 | 0 | 0 | 3 | 8 | 0 | 2 | 0 | 0 | DDE superfamily endonuclease |
| GF_7128 | 0 | 0 | 0 | 0 | 0 | 1 | 0 | 0 | 9 | 0 | 0 | Chromo (CHRromatin Organisation MOdifier) domain |
| GF_273 | 3 | 1 | 1 | 1 | 0 | 37 | 4 | 0 | 2 | 0 | 0 | Reverse transcriptase (RNA-dependent DNA polymerase) |
| GF_3247 | 0 | 0 | 0 | 0 | 0 | 2 | 1 | 0 | 9 | 0 | 0 | Lumazine binding domain |
| GF_10548 | 0 | 0 | 0 | 0 | 0 | 1 | 0 | 0 | 8 | 0 | 0 | DDE superfamily endonuclease |
| GF_116 | 7 | 7 | 7 | 7 | 7 | 7 | 2 | 2 | 7 | 7 | 7 | Fibrillar collagen C-terminal domain |
| GF_155 | 4 | 3 | 8 | 6 | 5 | 5 | 4 | 0 | 7 | 6 | 12 | Cytochrome P450 |
| GF_274 | 1 | 1 | 11 | 19 | 3 | 0 | 1 | 0 | 8 | 5 | 0 | Core histone H2A/H2B/H3/H4 |
| GF_447 | 0 | 0 | 0 | 0 | 0 | 1 | 0 | 0 | 39 | 0 | 0 | Peptidase family M50 |

**Supplementary Table 5.4** Contracted gene families in the Manado pipefish genome

| Family_ID | Medaka | Platyfish | Nile tilapia | Stickleback | Green spotted puffer | Tiger tail seahorse | Lined seahorse | Gulf pipefish | Manado Pipefish | Mudskipper | Zebrafish | Description |
| --- | --- | --- | --- | --- | --- | --- | --- | --- | --- | --- | --- | --- |
| GF_1247 | 0 | 0 | 0 | 0 | 0 | 0 | 22 | 0 | 0 | 0 | 0 | Retrotransposon gag protein |
| GF_10582 | 0 | 0 | 0 | 0 | 0 | 0 | 9 | 0 | 0 | 0 | 0 | Retrotransposon gag protein |
| GF_14931 | 2 | 0 | 0 | 0 | 0 | 3 | 0 | 0 | 0 | 0 | 0 | Reverse transcriptase (RNA-dependent DNA polymerase) |
| GF_12430 | 0 | 0 | 0 | 0 | 0 | 0 | 8 | 0 | 0 | 0 | 0 | Protein of unknown function (DUF2992) |
| GF_10570 | 0 | 0 | 0 | 1 | 0 | 2 | 6 | 0 | 0 | 0 | 0 | Reverse transcriptase (RNA-dependent DNA polymerase) |
| GF_15104 | 0 | 0 | 0 | 0 | 0 | 0 | 5 | 0 | 0 | 0 | 0 | Peptidase M16 inactive domain |
| GF_14387 | 0 | 0 | 0 | 0 | 0 | 0 | 6 | 0 | 0 | 0 | 0 | Transposase |
| GF_361 | 0 | 0 | 1 | 0 | 0 | 1 | 41 | 0 | 0 | 0 | 0 | Bacterial extracellular solute-binding proteins, family 5 Middle |
| GF_30 | 0 | 0 | 0 | 0 | 0 | 1 | 115 | 0 | 0 | 0 | 0 | Trypsin |
| GF_10571 | 0 | 0 | 0 | 0 | 0 | 0 | 9 | 0 | 0 | 0 | 0 | Adenylate kinase |
| GF_258 | 0 | 0 | 0 | 0 | 0 | 24 | 22 | 0 | 0 | 0 | 4 | Reverse transcriptase (RNA-dependent DNA polymerase) |
| GF_1902 | 0 | 0 | 0 | 0 | 0 | 0 | 19 | 0 | 0 | 0 | 0 | Multi-copper polyphenol oxidoreductase laccase |
| GF_47 | 0 | 0 | 0 | 0 | 0 | 1 | 95 | 0 | 0 | 0 | 0 | Reverse transcriptase (RNA-dependent DNA polymerase) |
| GF_14262 | 0 | 0 | 0 | 0 | 0 | 6 | 0 | 0 | 0 | 0 | 0 | Endonuclease-reverse transcriptase |
| GF_12392 | 0 | 0 | 0 | 0 | 0 | 0 | 8 | 0 | 0 | 0 | 0 | Ankyrin repeats (3 copies) |
| GF_12402 | 0 | 0 | 0 | 0 | 0 | 1 | 7 | 0 | 0 | 0 | 0 | Retroviral aspartyl protease |
| GF_3311 | 0 | 0 | 0 | 0 | 0 | 0 | 12 | 0 | 0 | 0 | 0 | Uncharacterised protein, DegV family COG1307 |
| GF_420 | 0 | 0 | 0 | 0 | 0 | 0 | 41 | 0 | 0 | 0 | 0 | MAPEG family |
| GF_14313 | 1 | 0 | 0 | 4 | 0 | 1 | 0 | 0 | 0 | 0 | 0 | HECT-domain (ubiquitin-transferase) |
| GF_12282 | 0 | 0 | 0 | 0 | 0 | 8 | 0 | 0 | 0 | 0 | 0 | Reverse transcriptase (RNA-dependent DNA polymerase) |
| GF_1903 | 0 | 0 | 0 | 0 | 0 | 0 | 19 | 0 | 0 | 0 | 0 | Protein kinase domain |
| GF_12272 | 1 | 0 | 0 | 0 | 0 | 1 | 4 | 2 | 0 | 0 | 0 | Immunoglobulin V-set domain |
| GF_15095 | 0 | 0 | 0 | 0 | 0 | 0 | 5 | 0 | 0 | 0 | 0 | Reverse transcriptase (RNA-dependent DNA polymerase) |
| GF_750 | 0 | 0 | 0 | 0 | 0 | 30 | 0 | 0 | 0 | 0 | 0 | Phosphoribosyl synthetase-associated domain |
| GF_532 | 0 | 0 | 0 | 0 | 0 | 0 | 36 | 0 | 0 | 0 | 0 | Sel1 repeat |
| GF_2375 | 1 | 0 | 0 | 0 | 0 | 14 | 0 | 0 | 0 | 0 | 0 | 50S ribosome-binding GTPase |
| GF_978 | 0 | 0 | 0 | 0 | 0 | 0 | 25 | 0 | 0 | 0 | 0 | Reverse transcriptase (RNA-dependent DNA polymerase) |
| GF_4081 | 0 | 1 | 1 | 0 | 0 | 2 | 6 | 1 | 0 | 0 | 0 | Reverse transcriptase (RNA-dependent DNA polymerase) |
| GF_2510 | 13 | 0 | 0 | 0 | 0 | 1 | 0 | 0 | 0 | 0 | 0 | L1 transposable element |
| GF_166 | 4 | 5 | 10 | 8 | 3 | 6 | 4 | 8 | 4 | 4 | 2 | GDSL/SGNH-like Acyl-Esterase family found in Pmr5 and Cas1p |
| GF_2603 | 1 | 12 | 0 | 0 | 0 | 0 | 1 | 0 | 0 | 0 | 0 | Peptidase family M50 |
| GF_12273 | 0 | 0 | 0 | 0 | 0 | 0 | 6 | 2 | 0 | 0 | 0 | Retroviral aspartyl protease |
| GF_6952 | 2 | 0 | 6 | 1 | 0 | 1 | 0 | 0 | 0 | 0 | 0 | hAT family C-terminal dimerisation region |
| GF_7172 | 0 | 0 | 0 | 0 | 0 | 0 | 10 | 0 | 0 | 0 | 0 | Reverse transcriptase (RNA-dependent DNA polymerase) |
| GF_1014 | 0 | 0 | 0 | 0 | 0 | 2 | 22 | 0 | 0 | 0 | 0 | Reverse transcriptase (RNA-dependent DNA polymerase) |
| GF_1953 | 0 | 0 | 0 | 0 | 0 | 18 | 0 | 0 | 0 | 0 | 0 | RhoGEF domain |
| GF_376 | 0 | 0 | 2 | 0 | 0 | 16 | 22 | 2 | 0 | 0 | 0 | Reverse transcriptase (RNA-dependent DNA polymerase) |
| GF_10425 | 0 | 0 | 0 | 0 | 0 | 8 | 1 | 0 | 0 | 0 | 0 | Leucine rich repeat |
| GF_31 | 27 | 2 | 40 | 1 | 2 | 0 | 0 | 0 | 1 | 39 | 1 | SPRY domain |
| GF_13400 | 0 | 0 | 0 | 0 | 0 | 7 | 0 | 0 | 0 | 0 | 0 | hAT family C-terminal dimerisation region |
| GF_2248 | 1 | 0 | 0 | 0 | 0 | 14 | 1 | 0 | 0 | 0 | 0 | Reverse transcriptase (RNA-dependent DNA polymerase) |
| GF_10578 | 0 | 0 | 0 | 0 | 0 | 0 | 9 | 0 | 0 | 0 | 0 | Endonuclease/Exonuclease/phosphatase family |
| GF_14253 | 0 | 0 | 0 | 0 | 0 | 6 | 0 | 0 | 0 | 0 | 0 | WD domain, G-beta repeat |
| GF_13530 | 0 | 0 | 0 | 0 | 0 | 0 | 7 | 0 | 0 | 0 | 0 | Endonuclease/Exonuclease/phosphatase family |
| GF_10 | 19 | 9 | 31 | 13 | 11 | 6 | 5 | 2 | 5 | 22 | 56 | Receptor family ligand binding region |
| GF_14932 | 0 | 0 | 0 | 0 | 0 | 5 | 0 | 0 | 0 | 0 | 0 | Protein of unknown function (DUF2499) |
| GF_10424 | 0 | 0 | 0 | 0 | 0 | 9 | 0 | 0 | 0 | 0 | 0 | Zinc-finger associated domain (zf-AD) |
| GF_13415 | 6 | 0 | 0 | 0 | 0 | 1 | 0 | 0 | 0 | 0 | 0 | E1-E2 ATPase |
| GF_15093 | 0 | 0 | 0 | 0 | 0 | 0 | 5 | 0 | 0 | 0 | 0 | Tripartite tricarboxylate transporter family receptor |
| GF_15102 | 0 | 0 | 0 | 0 | 0 | 0 | 5 | 0 | 0 | 0 | 0 | Nucleoside transporter |
| GF_3302 | 0 | 0 | 0 | 0 | 0 | 0 | 12 | 0 | 0 | 0 | 0 | Reverse transcriptase (RNA-dependent DNA polymerase) |
| GF_7061 | 0 | 0 | 1 | 6 | 0 | 0 | 1 | 1 | 0 | 1 | 0 | Immunoglobulin V-set domain |
| GF_14382 | 0 | 0 | 0 | 0 | 0 | 0 | 6 | 0 | 0 | 0 | 0 | Reverse transcriptase (RNA-dependent DNA polymerase) |
| GF_14252 | 0 | 0 | 0 | 0 | 0 | 6 | 0 | 0 | 0 | 0 | 0 | Adaptin N terminal region |
| GF_2265 | 15 | 0 | 0 | 0 | 0 | 1 | 0 | 0 | 0 | 0 | 0 | DDE superfamily endonuclease |
| GF_13401 | 0 | 0 | 0 | 0 | 0 | 7 | 0 | 0 | 0 | 0 | 0 | Protein of unknown function (DUF2499) |
| GF_805 | 0 | 0 | 0 | 0 | 0 | 29 | 0 | 0 | 0 | 0 | 0 | AAA ATPase domain |
| GF_37 | 1 | 2 | 21 | 34 | 1 | 1 | 1 | 0 | 2 | 26 | 17 | SPRY domain |
| GF_10434 | 0 | 0 | 0 | 0 | 0 | 9 | 0 | 0 | 0 | 0 | 0 | Diaphanous FH3 Domain |
| GF_3190 | 0 | 0 | 10 | 0 | 0 | 1 | 0 | 0 | 0 | 1 | 0 | Lectin C-type domain |
| GF_1072 | 22 | 0 | 0 | 0 | 0 | 1 | 0 | 0 | 0 | 0 | 0 | DDE superfamily endonuclease |
| GF_303 | 5 | 6 | 9 | 4 | 7 | 1 | 1 | 0 | 1 | 4 | 8 | Cytochrome P450 |
| GF_2596 | 0 | 0 | 0 | 0 | 0 | 0 | 14 | 0 | 0 | 0 | 0 | ATP synthase alpha/beta family, beta-barrel domain |
| GF_14379 | 0 | 0 | 0 | 0 | 0 | 0 | 6 | 0 | 0 | 0 | 0 | Histidine kinase |
| GF_14251 | 1 | 0 | 0 | 0 | 0 | 5 | 0 | 0 | 0 | 0 | 0 | Leucine rich repeat |
| GF_14934 | 0 | 0 | 0 | 0 | 0 | 5 | 0 | 0 | 0 | 0 | 0 | DDE superfamily endonuclease |
| GF_2 | 0 | 0 | 0 | 0 | 0 | 1250 | 0 | 0 | 0 | 0 | 0 | Pilin accessory protein (PilO) |
| GF_13 | 0 | 0 | 0 | 0 | 0 | 165 | 0 | 0 | 0 | 0 | 0 | Pilin accessory protein (PilO) |
| GF_1 | 0 | 0 | 0 | 0 | 0 | 1257 | 0 | 0 | 0 | 0 | 0 | Nitrite/Sulfite reductase ferredoxin-like half domain |
| GF_304 | 1 | 2 | 35 | 7 | 0 | 1 | 0 | 0 | 0 | 0 | 0 | SPRY domain |
| GF_14261 | 2 | 0 | 0 | 0 | 0 | 4 | 0 | 0 | 0 | 0 | 0 | 50S ribosome-binding GTPase |
| GF_46 | 11 | 0 | 1 | 21 | 7 | 4 | 7 | 0 | 2 | 11 | 32 | Core histone H2A/H2B/H3/H4 |
| GF_10583 | 0 | 0 | 0 | 0 | 0 | 1 | 8 | 0 | 0 | 0 | 0 | META domain |
| GF_276 | 0 | 0 | 0 | 0 | 0 | 1 | 0 | 0 | 0 | 0 | 48 | Immunoglobulin V-set domain |
| GF_1808 | 2 | 2 | 10 | 0 | 0 | 1 | 1 | 0 | 0 | 2 | 1 | Class I Histocompatibility antigen, domains alpha 1 and 2 |
| GF_14933 | 0 | 0 | 0 | 0 | 0 | 5 | 0 | 0 | 0 | 0 | 0 | Uncharacterized conserved protein (DUF2075) |
| GF_746 | 1 | 3 | 3 | 0 | 1 | 4 | 4 | 0 | 0 | 0 | 14 | Reverse transcriptase (RNA-dependent DNA polymerase) |
| GF_1947 | 1 | 0 | 0 | 0 | 0 | 10 | 7 | 0 | 0 | 0 | 0 | Reverse transcriptase (RNA-dependent DNA polymerase) |
| GF_949 | 3 | 1 | 4 | 5 | 1 | 2 | 1 | 0 | 1 | 0 | 7 | Immunoglobulin C1-set domain |
| GF_486 | 2 | 2 | 5 | 3 | 11 | 2 | 1 | 2 | 2 | 5 | 3 | MatE |
| GF_1518 | 0 | 0 | 0 | 0 | 0 | 20 | 0 | 0 | 0 | 0 | 0 | 4Fe-4S dicluster domain |
| GF_15108 | 0 | 0 | 0 | 0 | 0 | 0 | 5 | 0 | 0 | 0 | 0 | Glycosyl hydrolases family 39 |
| GF_1468 | 0 | 0 | 0 | 0 | 0 | 0 | 21 | 0 | 0 | 0 | 0 | Reverse transcriptase (RNA-dependent DNA polymerase) |
| GF_12404 | 0 | 0 | 0 | 0 | 0 | 1 | 7 | 0 | 0 | 0 | 0 | Reverse transcriptase (RNA-dependent DNA polymerase) |
| GF_411 | 1 | 3 | 15 | 2 | 1 | 3 | 3 | 0 | 2 | 7 | 4 | Root hair defective 3 GTP-binding protein (RHD3) |
| GF_15091 | 0 | 0 | 0 | 0 | 0 | 0 | 5 | 0 | 0 | 0 | 0 | Vta1 like |
| GF_14249 | 1 | 0 | 0 | 0 | 0 | 4 | 1 | 0 | 0 | 0 | 0 | Flagellar biosynthesis protein, FliO |
| GF_895 | 0 | 0 | 0 | 0 | 0 | 1 | 27 | 0 | 0 | 0 | 0 | Leucine rich repeats (6 copies) |
| GF_6916 | 0 | 0 | 1 | 0 | 0 | 8 | 0 | 1 | 0 | 0 | 0 | DDE superfamily endonuclease |
| GF_3 | 12 | 16 | 163 | 2 | 12 | 1 | 1 | 1 | 1 | 82 | 337 | NACHT domain |
| GF_14399 | 0 | 0 | 0 | 0 | 0 | 0 | 6 | 0 | 0 | 0 | 0 | Branched-chain amino acid transport system / permease component |
| GF_2267 | 13 | 1 | 1 | 0 | 0 | 0 | 0 | 1 | 0 | 0 | 0 | Immunoglobulin V-set domain |
| GF_618 | 0 | 0 | 0 | 0 | 0 | 0 | 33 | 0 | 0 | 0 | 0 | Endonuclease/Exonuclease/phosphatase family |
| GF_188 | 0 | 0 | 1 | 0 | 0 | 2 | 52 | 0 | 0 | 0 | 0 | Reverse transcriptase (RNA-dependent DNA polymerase) |
| GF_13536 | 0 | 0 | 0 | 0 | 0 | 1 | 6 | 0 | 0 | 0 | 0 | Integrase core domain |
| GF_13528 | 0 | 0 | 0 | 0 | 0 | 0 | 7 | 0 | 0 | 0 | 0 | Acetyltransferase (GNAT) domain |
| GF_39 | 8 | 8 | 16 | 11 | 6 | 10 | 8 | 4 | 8 | 14 | 11 | Hsp70 protein |
| GF_14951 | 4 | 0 | 0 | 0 | 0 | 1 | 0 | 0 | 0 | 0 | 0 | hAT family C-terminal dimerisation region |
| GF_7054 | 1 | 0 | 0 | 6 | 0 | 1 | 2 | 0 | 0 | 0 | 0 | Immunoglobulin V-set domain |
| GF_4068 | 0 | 0 | 0 | 0 | 0 | 0 | 11 | 0 | 0 | 0 | 0 | Endonuclease/Exonuclease/phosphatase family |
| GF_12398 | 0 | 0 | 0 | 0 | 0 | 0 | 7 | 0 | 0 | 0 | 1 | Retrotransposon gag protein |
| GF_792 | 6 | 3 | 2 | 4 | 1 | 4 | 1 | 1 | 2 | 3 | 3 | Protein kinase domain |
| GF_2331 | 0 | 0 | 0 | 0 | 0 | 0 | 16 | 0 | 0 | 0 | 0 | Reverse transcriptase (RNA-dependent DNA polymerase) |
| GF_2243 | 2 | 0 | 3 | 8 | 0 | 1 | 0 | 2 | 0 | 0 | 0 | Domain of unknown function (DUF4371) |
| GF_13534 | 0 | 0 | 0 | 0 | 0 | 0 | 7 | 0 | 0 | 0 | 0 | NADH dehydrogenase subunit 2 C-terminus |
| GF_10419 | 1 | 0 | 0 | 0 | 1 | 4 | 1 | 2 | 0 | 0 | 0 | SPRY domain |
| GF_419 | 0 | 0 | 0 | 0 | 0 | 0 | 41 | 0 | 0 | 0 | 0 | Reverse transcriptase (RNA-dependent DNA polymerase) |
| GF_10439 | 1 | 0 | 2 | 1 | 0 | 4 | 1 | 0 | 0 | 0 | 0 | Domain of unknown function (DUF4371) |
| GF_594 | 1 | 0 | 0 | 0 | 0 | 32 | 0 | 0 | 0 | 0 | 0 | PAP2 superfamily |
| GF_10452 | 5 | 0 | 1 | 0 | 0 | 2 | 1 | 0 | 0 | 0 | 0 | Endonuclease/Exonuclease/phosphatase family |
| GF_266 | 0 | 0 | 0 | 0 | 0 | 3 | 1 | 0 | 0 | 46 | 0 | Peptidase family M50 |
| GF_10533 | 0 | 0 | 2 | 0 | 0 | 1 | 6 | 0 | 0 | 0 | 0 | Integrase core domain |
| GF_10572 | 0 | 0 | 0 | 0 | 0 | 1 | 7 | 1 | 0 | 0 | 0 | Domain of unknown function (DUF3523) |
| GF_927 | 0 | 0 | 0 | 0 | 0 | 26 | 0 | 0 | 0 | 0 | 0 | Endonuclease/Exonuclease/phosphatase family |
| GF_533 | 0 | 0 | 0 | 0 | 0 | 1 | 35 | 0 | 0 | 0 | 0 | Hsp20/alpha crystallin family |
| GF_12391 | 0 | 0 | 0 | 0 | 0 | 0 | 8 | 0 | 0 | 0 | 0 | Reverse transcriptase (RNA-dependent DNA polymerase) |
| GF_6948 | 2 | 0 | 0 | 3 | 0 | 1 | 4 | 0 | 0 | 0 | 0 | Sel1 repeat |
| GF_3876 | 0 | 0 | 3 | 7 | 0 | 1 | 0 | 0 | 0 | 0 | 0 | Immunoglobulin V-set domain |
| GF_514 | 2 | 1 | 11 | 2 | 2 | 1 | 1 | 1 | 1 | 3 | 12 | Scavenger receptor cysteine-rich domain |
| GF_13539 | 0 | 0 | 0 | 0 | 0 | 0 | 7 | 0 | 0 | 0 | 0 | Endonuclease/Exonuclease/phosphatase family |
| GF_362 | 1 | 0 | 0 | 0 | 0 | 0 | 42 | 0 | 0 | 0 | 0 | Reverse transcriptase (RNA-dependent DNA polymerase) |
| GF_14392 | 0 | 0 | 0 | 0 | 0 | 0 | 6 | 0 | 0 | 0 | 0 | ROK family |
| GF_992 | 0 | 0 | 0 | 0 | 0 | 24 | 0 | 0 | 0 | 0 | 0 | Diaphanous FH3 Domain |
| GF_1368 | 0 | 1 | 8 | 3 | 0 | 2 | 4 | 0 | 0 | 0 | 3 | DDE superfamily endonuclease |
| GF_15092 | 0 | 0 | 0 | 0 | 0 | 0 | 5 | 0 | 0 | 0 | 0 | Peptidase M16 inactive domain |
| GF_2208 | 0 | 0 | 0 | 0 | 0 | 0 | 17 | 0 | 0 | 0 | 0 | Protein of unknown function N-terminal domain (DUF2450) |
| GF_3301 | 0 | 0 | 0 | 0 | 0 | 0 | 12 | 0 | 0 | 0 | 0 | Endonuclease/Exonuclease/phosphatase family |
| GF_794 | 0 | 0 | 0 | 0 | 0 | 0 | 30 | 0 | 0 | 0 | 0 | Reverse transcriptase (RNA-dependent DNA polymerase) |
| GF_12390 | 0 | 0 | 0 | 0 | 0 | 0 | 8 | 0 | 0 | 0 | 0 | Endonuclease/Exonuclease/phosphatase family |
| GF_6949 | 9 | 0 | 0 | 0 | 0 | 1 | 0 | 0 | 0 | 0 | 0 | Endonuclease/Exonuclease/phosphatase family |
| GF_2937 | 0 | 0 | 0 | 0 | 0 | 12 | 0 | 0 | 0 | 0 | 0 | 50S ribosome-binding GTPase |
| GF_14385 | 0 | 0 | 0 | 0 | 0 | 0 | 6 | 0 | 0 | 0 | 0 | DDE superfamily endonuclease |
| GF_991 | 1 | 0 | 2 | 0 | 0 | 21 | 0 | 0 | 0 | 0 | 0 | Reverse transcriptase (RNA-dependent DNA polymerase) |
| GF_2936 | 2 | 0 | 0 | 0 | 0 | 7 | 3 | 0 | 0 | 0 | 0 | Domain of unknown function (DUF4371) |
| GF_2422 | 0 | 0 | 0 | 14 | 0 | 1 | 0 | 0 | 0 | 0 | 0 | DDE superfamily endonuclease |
| GF_15820 | 1 | 0 | 0 | 0 | 0 | 0 | 3 | 0 | 0 | 0 | 0 | Retrotransposon gag protein |
| GF_2057 | 0 | 0 | 0 | 0 | 0 | 0 | 18 | 0 | 0 | 0 | 0 | Saccharopine dehydrogenase |
| GF_3317 | 0 | 0 | 0 | 0 | 0 | 1 | 11 | 0 | 0 | 0 | 0 | CBS domain |
| GF_13408 | 0 | 0 | 0 | 0 | 0 | 7 | 0 | 0 | 0 | 0 | 0 | Radical SAM superfamily |
| GF_12399 | 0 | 0 | 0 | 0 | 0 | 0 | 8 | 0 | 0 | 0 | 0 | Reverse transcriptase (RNA-dependent DNA polymerase) |
| GF_12401 | 0 | 0 | 0 | 0 | 0 | 0 | 8 | 0 | 0 | 0 | 0 | Endonuclease/Exonuclease/phosphatase family |
| GF_174 | 4 | 1 | 0 | 10 | 5 | 2 | 3 | 0 | 1 | 6 | 25 | Core histone H2A/H2B/H3/H4 |
| GF_1007 | 0 | 0 | 1 | 0 | 0 | 1 | 0 | 1 | 0 | 0 | 21 | Reverse transcriptase (RNA-dependent DNA polymerase) |
| GF_1248 | 1 | 0 | 0 | 0 | 0 | 0 | 21 | 0 | 0 | 0 | 0 | Reverse transcriptase (RNA-dependent DNA polymerase) |
| GF_14384 | 1 | 0 | 0 | 0 | 0 | 0 | 5 | 0 | 0 | 0 | 0 | Endonuclease/Exonuclease/phosphatase family |
| GF_6930 | 0 | 0 | 1 | 1 | 1 | 6 | 1 | 0 | 0 | 0 | 0 | Reverse transcriptase (RNA-dependent DNA polymerase) |
| GF_2056 | 0 | 0 | 0 | 0 | 0 | 0 | 18 | 0 | 0 | 0 | 0 | Endonuclease/Exonuclease/phosphatase family |
| GF_1469 | 0 | 0 | 0 | 0 | 0 | 0 | 21 | 0 | 0 | 0 | 0 | Saccharopine dehydrogenase |
| GF_10576 | 0 | 0 | 0 | 0 | 0 | 0 | 9 | 0 | 0 | 0 | 0 | tRNA synthetases class I (I, L, M and V) |
| GF_13538 | 0 | 0 | 0 | 0 | 0 | 0 | 7 | 0 | 0 | 0 | 0 | Jumping translocation breakpoint protein (JTB) |
| GF_15125 | 0 | 0 | 0 | 0 | 0 | 5 | 0 | 0 | 0 | 0 | 0 | Spt5 C-terminal nonapeptide repeat binding Spt4 |
| GF_2815 | 0 | 1 | 0 | 0 | 0 | 0 | 12 | 0 | 0 | 0 | 0 | DDE superfamily endonuclease |
| GF_14391 | 0 | 0 | 0 | 0 | 0 | 0 | 6 | 0 | 0 | 0 | 0 | Thrombospondin type 3 repeat |
| GF_2500 | 0 | 0 | 0 | 0 | 0 | 14 | 0 | 0 | 0 | 0 | 0 | Nitrite/Sulfite reductase ferredoxin-like half domain |
| GF_13535 | 0 | 0 | 0 | 0 | 0 | 1 | 6 | 0 | 0 | 0 | 0 | Phosphoribosyl transferase domain |
| GF_2271 | 15 | 0 | 0 | 0 | 0 | 1 | 0 | 0 | 0 | 0 | 0 | Endonuclease/Exonuclease/phosphatase family |
| GF_12400 | 0 | 0 | 0 | 0 | 0 | 0 | 8 | 0 | 0 | 0 | 0 | Thrombospondin type 1 domain |
| GF_1951 | 0 | 0 | 2 | 1 | 1 | 8 | 6 | 0 | 0 | 0 | 0 | Reverse transcriptase (RNA-dependent DNA polymerase) |
| GF_731 | 6 | 3 | 2 | 8 | 3 | 1 | 1 | 1 | 0 | 4 | 2 | Receptor family ligand binding region |
| GF_13533 | 0 | 0 | 0 | 0 | 0 | 0 | 7 | 0 | 0 | 0 | 0 | Reverse transcriptase (RNA-dependent DNA polymerase) |
| GF_13526 | 0 | 0 | 0 | 0 | 0 | 1 | 6 | 0 | 0 | 0 | 0 | Immunoglobulin domain |
| GF_2115 | 0 | 0 | 5 | 1 | 0 | 11 | 0 | 0 | 0 | 0 | 0 | Domain of unknown function (DUF4371) |
| GF_10575 | 0 | 0 | 0 | 0 | 0 | 0 | 9 | 0 | 0 | 0 | 0 | zinc-binding in reverse transcriptase |

**Supplementary Table 5.5** Expanded and contracted gene families in the common ancestor of syngnathids.

| Family_ID | Expansion (+)/ Contraction (-) | Annotation | Description |
| --- | --- | --- | --- |
| GF_543 | + | PF00078.22 | Reverse transcriptase (RNA-dependent DNA polymerase) |
| GF_14952 | - | PF00001.16 | 7 transmembrane receptor (rhodopsin family) |
| GF_10507 | - | PF04130.8 | Spc97 / Spc98 family |
| GF_7055 | - | PF00001.16 | 7 transmembrane receptor (rhodopsin family) |
| GF_2751 | - | PF00622.23 | SPRY domain |
| GF_10531 | - | PF01223.18 | DNA/RNA non-specific endonuclease |
| GF_10616 | - | PF03938.9 | Outer membrane protein (OmpH-like) |
| GF_14950 | - | PF00078.22 | Reverse transcriptase (RNA-dependent DNA polymerase) |
| GF_6918 | - | PF07686.12 | Immunoglobulin V-set domain |
| GF_1243 | - | PF00059.16 | Lectin C-type domain |
| GF_303 | - | PF00067.17 | Cytochrome P450 |
| GF_2425 | - | PF00129.13 | Class I Histocompatibility antigen, domains alpha 1 and 2 |
| GF_15128 | - | PF07690.11 | Major Facilitator Superfamily |
| GF_12341 | - | PF00001.16 | 7 transmembrane receptor (rhodopsin family) |
| GF_3169 | - | PF04937.10 | Protein of unknown function (DUF 659) |
| GF_3133 | - | PF00078.22 | Reverse transcriptase (RNA-dependent DNA polymerase) |
| GF_476 | - | PF13472.1 | GDSL-like Lipase/Acylhydrolase family |
| GF_664 | - | PF00990.16 | GGDEF domain |
| GF_3366 | - | PF02580.11 | D-Tyr-tRNA(Tyr) deacylase |
| GF_31 | - | PF00622.23 | SPRY domain |
| GF_10 | - | PF01094.23 | Receptor family ligand binding region |
| GF_13422 | - | PF06367.11 | Diaphanous FH3 Domain |
| GF_14408 | - | PF00105.13 | Zinc finger, C4 type (two domains) |
| GF_14954 |  | PF00059.16 | Lectin C-type domain |
| GF_12342 | - | PF00735.13 | Septin |
| GF_10427 | - | PF12799.2 | Leucine Rich repeats (2 copies) |
| GF_1824 | - | PF02163.17 | Peptidase family M50 |
| GF_13418 |  | PF00098.18 | Zinc knuckle |
| GF_13501 | - | PF07686.12 | Immunoglobulin V-set domain |
| GF_2426 | - | PF02758.11 | PAAD/DAPIN/Pyrin domain |
| GF_14274 | - | PF07686.12 | Immunoglobulin V-set domain |
| GF_2569 | - | PF07686.12 | Immunoglobulin V-set domain |
| GF_1884 | - | PF07686.12 | Immunoglobulin V-set domain |
| GF_13556 | - | PF14580.1 | Leucine-rich repeat |
| GF_891 |  | PF00622.23 | SPRY domain |
| GF_3139 | - | PF04548.11 | AIG1 family |
| GF_2980 | - | PF00059.16 | Lectin C-type domain |
| GF_288 | - | PF02163.17 | Peptidase family M50 |
| GF_1696 | - | PF13895.1 | Immunoglobulin domain |
| GF_2775 | - | PF04548.11 | AIG1 family |
| GF_6943 | - | PF07686.12 | Immunoglobulin V-set domain |
| GF_1890 | - | PF00059.16 | Lectin C-type domain |
| GF_13473 | - | PF02023.12 | SCAN domain |
| GF_13416 | - | PF00078.22 | Reverse transcriptase (RNA-dependent DNA polymerase) |
| GF_14 | - | PF02163.17 | Peptidase family M50 |
| GF_1198 | - | PF13472.1 | GDSL-like Lipase/Acylhydrolase family |
| GF_15126 | - | PF11951.3 | Fungal specific transcription factor domain |
| GF_12426 | - | PF06687.7 | SUR7/PalI family |
| GF_3400 | - | PF00070.22 | Pyridine nucleotide-disulphide oxidoreductase |
| GF_1011 | - | PF13895.1 | Immunoglobulin domain |
| GF_2773 | - | PF00059.16 | Lectin C-type domain |
| GF_14310 | - | PF00643.19 | B-box zinc finger |
| GF_731 | - | PF01094.23 | Receptor family ligand binding region |
| GF_15119 | - | PF00059.16 | Lectin C-type domain |
| GF_1090 | - | PF07654.10 | Immunoglobulin C1-set domain |
| GF_2776 | - | PF00059.16 | Lectin C-type domain |
| GF_10528 | - | PF00129.13 | Class I Histocompatibility antigen, domains alpha 1 and 2 |
| GF_2193 | - | PF00125.19 | Core histone H2A/H2B/H3/H4 |
| GF_14406 | - | PF00443.24 | Ubiquitin carboxyl-terminal hydrolase |
| GF_4182 | - | PF00520.26 | Ion transport protein |
| GF_37 | - | PF00622.23 | SPRY domain |
| GF_13499 | - | PF07686.12 | Immunoglobulin V-set domain |
| GF_3 | - | PF05729.7 | NACHT domain |
| GF_553 | - | PF03372.18 | Endonuclease/Exonuclease/phosphatase family |
| GF_14959 | - | PF00632.20 | HECT-domain (ubiquitin-transferase) |
| GF_156 | - | PF05729.7 | NACHT domain |
| GF_6997 | - | PF00074.15 | Pancreatic ribonuclease |
| GF_14946 | - | PF07686.12 | Immunoglobulin V-set domain |
| GF_793 | - | PF05879.7 | Root hair defective 3 GTP-binding protein (RHD3) |
| GF_2971 | - | PF08284.6 | Retroviral aspartyl protease |
| GF_17 | - | PF14484.1 | Fish-specific NACHT associated domain |
| GF_1705 | - | PF00622.23 | SPRY domain |
| GF_2450 | - | PF07686.12 | Immunoglobulin V-set domain |
| GF_3886 | - | PF00001.16 | 7 transmembrane receptor (rhodopsin family) |
| GF_2566 | - | PF00004.24 | ATPase family associated with various cellular activities (AAA) |
| GF_10428 | - | PF00447.12 | HSF-type DNA-binding |
| GF_2504 | - | PF07686.12 | Immunoglobulin V-set domain |
| GF_3191 | - | PF00059.16 | Lectin C-type domain |
| GF_14272 | - | PF07654.10 | Immunoglobulin C1-set domain |
| GF_73 | - | PF13895.1 | Immunoglobulin domain |
| GF_12414 | - | PF00059.16 | Lectin C-type domain |
| GF_1699 | - | PF07686.12 | Immunoglobulin V-set domain |
| GF_2212 | - | PF07117.6 | Protein of unknown function (DUF1373) |
| GF_14958 | - | PF00001.16 | 7 transmembrane receptor (rhodopsin family) |
| GF_14953 | - | PF02994.9 | L1 transposable element |
| GF_14409 | - | PF00271.26 | Helicase conserved C-terminal domain |
| GF_1458 | - | PF00089.21 | Trypsin |
| GF_7052 | - | PF00125.19 | Core histone H2A/H2B/H3/H4 |
| GF_14266 | - | PF00069.20 | Protein kinase domain |
| GF_4181 | - | PF04434.12 | SWIM zinc finger |
| GF_7065 | - | PF00001.16 | 7 transmembrane receptor (rhodopsin family) |
| GF_893 | - | PF04548.11 | AIG1 family |
| GF_2580 | - | PF00386.16 | C1q domain |
| GF_13485 | - | PF00001.16 | 7 transmembrane receptor (rhodopsin family) |
| GF_14255 | - | PF00069.20 | Protein kinase domain |
| GF_13402 | - | PF05049.8 | Interferon-inducible GTPase (IIGP) |
| GF_12425 | - | PF00226.26 | DnaJ domain |
| GF_46 | - | PF00125.19 | Core histone H2A/H2B/H3/H4 |
| GF_2046 | - | PF00001.16 | 7 transmembrane receptor (rhodopsin family) |
| GF_2579 | - | PF00089.21 | Trypsin |
| GF_3130 | - | PF13359.1 | DDE superfamily endonuclease |
| GF_856 | - | PF07686.12 | Immunoglobulin V-set domain |
| GF_12336 | - | PF00078.22 | Reverse transcriptase (RNA-dependent DNA polymerase) |
| GF_6944 | - | PF15140.1 | Domain of unknown function (DUF4573) |
| GF_14966 | - | PF00593.19 | TonB dependent receptor |
| GF_10615 | - | PF13927.1 | Immunoglobulin domain |
| GF_200 | - | PF13895.1 | Immunoglobulin domain |
| GF_2333 | - | PF02163.17 | Peptidase family M50 |
| GF_7077 | - | PF04937.10 | Protein of unknown function (DUF 659) |
| GF_3883 | - | PF00538.14 | linker histone H1 and H5 family |
| GF_3721 | - | PF07686.12 | Immunoglobulin V-set domain |
| GF_3140 | - | PF07686.12 | Immunoglobulin V-set domain |
| GF_12337 | - | PF14484.1 | Fish-specific NACHT associated domain |
| GF_3864 | - | PF07686.12 | Immunoglobulin V-set domain |
| GF_3665 | - | PF07686.12 | Immunoglobulin V-set domain |
| GF_89 | - | PF00622.23 | SPRY domain |
| GF_3166 | - | PF13895.1 | Immunoglobulin domain |
| GF_3157 | - | PF00001.16 | 7 transmembrane receptor (rhodopsin family) |
| GF_13495 | - | PF07686.12 | Immunoglobulin V-set domain |
| GF_13417 | - | PF07686.12 | Immunoglobulin V-set domain |
| GF_2392 | - | PF07686.12 | Immunoglobulin V-set domain |
| GF_7085 | - | PF04548.11 | AIG1 family |
| GF_2690 | - | PF14484.1 | Fish-specific NACHT associated domain |
| GF_1071 | - | PF13426.1 | PAS domain |
| GF_7045 | - | PF07686.12 | Immunoglobulin V-set domain |
| GF_14947 | - | PF02752.17 | Arrestin (or S-antigen), C-terminal domain |
| GF_14270 | - | PF13358.1 | DDE superfamily endonuclease |
| GF_14321 | - | PF05729.7 | NACHT domain |
| GF_3195 | - | PF07686.12 | Immunoglobulin V-set domain |
| GF_12289 | - | PF13246.1 | Putative hydrolase of sodium-potassium ATPase alpha subunit |
| GF_2409 | - | PF01823.14 | MAC/Perforin domain |
| GF_2042 | - | PF07686.12 | Immunoglobulin V-set domain |
| GF_1083 | - | PF04103.10 | CD20-like family |
| GF_15127 | - | PF03104.14 | DNA polymerase family B, exonuclease domain |
| GF_13428 | - | PF00089.21 | Trypsin |
| GF_314 | - | PF03962.10 | Mnd1 family |
| GF_10525 | - | PF07654.10 | Immunoglobulin C1-set domain |
| GF_3185 | - | PF00129.13 | Class I Histocompatibility antigen, domains alpha 1 and 2 |
| GF_14407 | - | PF01182.15 | Glucosamine-6-phosphate isomerases/6-phosphogluconolactonase |
| GF_15124 | - | PF03382.9 | Mycoplasma protein of unknown function, DUF285 |
| GF_2774 | - | PF07686.12 | Immunoglobulin V-set domain |
| GF_3172 | - | PF00089.21 | Trypsin |
| GF_14955 | - | PF05699.9 | hAT family C-terminal dimerisation region |
| GF_13404 | - | PF00069.20 | Protein kinase domain |
| GF_2976 | - | PF00868.15 | Transglutaminase family |
| GF_14314 | - | PF07686.12 | Immunoglobulin V-set domain |
| GF_14322 | - | PF05699.9 | hAT family C-terminal dimerisation region |
| GF_2431 | - | PF00001.16 | 7 transmembrane receptor (rhodopsin family) |
| GF_10426 | - | PF00001.16 | 7 transmembrane receptor (rhodopsin family) |
| GF_14268 | - | PF00078.22 | Reverse transcriptase (RNA-dependent DNA polymerase) |
| GF_15120 | - | PF02163.17 | Peptidase family M50 |
| GF_12288 | - | PF07686.12 | Immunoglobulin V-set domain |
| GF_3898 | - | PF07686.12 | Immunoglobulin V-set domain |
| GF_2045 | - | PF04548.11 | AIG1 family |
| GF_3923 | - | PF04548.11 | AIG1 family |

# 6. Expansion of the protocadherin gene family

**Annotation methods**

We searched the Manado pipefish genome and transcriptome assemblies for protocadherin (Pcdh) and diaphanous genes by BLASTP and TBLASTN searches using sequences from zebrafish (*D. rerio*), human (*Homo sapiens*) and mouse (*Mus musculus*) as queries. To look specifically for clustered Pcdhs, we used constant region sequences of zebrafish α and γ Pcdh clusters as queries. Candidate genes were verified using BLAST and Pfam searches. Genes identified in the Manado pipefish genome were confirmed and extended using the transcriptomes, and multiple gene models that matched the same transcript were combined. The identified sequences from Manado pipefish were aligned using either MUSCLE [[26](#_ENREF_26)] or CLUSTALO [[27](#_ENREF_27)]. Phylogenetic trees were constructed with FastTree [[28](#_ENREF_28)] using full-length sequences, and members from each family were counted. The non-clustered Pcdh sequences were subjected to the phylogenetic analysis using the Maximum Likelihood (ML) method.

**Results**

Protocadherins are a group of transmembrane proteins belonging to the cadherin superfamily that are subgrouped into ‘clustered’ and ‘nonclustered’ protocadherins. Clustered protocadherins are involved in regulating neuronal survival. Combinatorial expression of clustered protocadherin isoforms generates tremendous diversity of adhesive specificity for cells. Non-clustered protocadherins promote cell motility rather than the stabilization of cell adhesion, unlike the classic cadherins, and mediate dynamic cellular processes, such as growth cone migration.

In human, the clustered protocadherins, organized into α-, β- and γ-subclusters, contain 15 to 22 large ‘‘variable’’ exons that are arranged in tandem [[29](#_ENREF_29)]. Each variable exon is transcribed from an independent promoter and encodes an extracellular domain comprising six calcium-binding ectodomain repeats (EC1-EC6), a transmembrane domain and a short segment of the intracellular domain. In addition to the variable exons, ends of the α- and γ-subclusters contain three ‘‘constant’’ exons each, which are spliced to individual variable exons in their respective subclusters. These constant exons encode a major part of the intracellular domain. The β-subcluster genes lack the constant exons, and contain only the diverse extracellular domain [[29](#_ENREF_29)]. Non-clustered Pcdhs are further classiﬁed into δ-Pcdhs and other Pcdhs, such as atypical Pcdhs like the protocadherin Fat. Although most Pcdhs have divergent cytoplasmic domain sequences, the δ-Pcdhs are characterized by the presence of unique common sequences, common motif 1 (CM1)-CM4. CM2 in particular, is highly conserved among the various δ-Pcdhs from different species [[30](#_ENREF_30)].

The protocadherin cluster represents one of the most evolutionarily dynamic gene loci in vertebrate genomes. Comparative analysis of its subcluster organization and paralog arrangement has provided useful information regarding the dynamic nature of vertebrate genomes [[31](#_ENREF_31), [32](#_ENREF_32)]. Teleost fishes such as zebrafish and fugu contain two unlinked protocadherin clusters, Pcdh1 and Pcdh2, due to a fish-specific genome duplication event [[33](#_ENREF_33), [34](#_ENREF_34)]. Both clusters lack the β-subcluster. The zebrafish Pcdh1 cluster possesses the δ-, α- and γ-subclusters whereas the Pcdh2 cluster has lost the δ-subcluster, thus retaining only the α- and γ-subclusters [[33](#_ENREF_33), [34](#_ENREF_34)]. In contrast, the fugu Pcdh1 cluster contains only the δ- and α-subclusters, and lacks the γ-subcluster [[34](#_ENREF_34)]. The duplicate protocadherin clusters in zebrafish and fugu contain at least 107 and 77 genes, respectively.

In order to identify the protocadherin cluster in the Manado pipefish, we performed TBLASTN searches against its genome using amino acid sequences of the zebrafish protocadherin constant exons as queries. This led to the identification of two scaffolds (Scaffold_57 and Scaffold_23) containing the entire clustered protocadherins and several nonclustered protocadherins. Annotation of this gene cluster using GENSCAN and homology comparisons identified 54 clustered protocadherins and 26 nonclustered protocadherins (Supplementary Figures S6.1 and S6.2).

In addition to the Pcdh genes, we also identified diaphanous (*Diaph*) homologues. In the human and elephant shark genome, the clustered *Pcdh* genes are closely linked to *Diaph1*; whereas *Diaph2* and *Diaph3* are linked to *Pcdh19* and *Pcdh17*, respectively. The Manado pipefish genome also contains three Diaph genes (Supplementary Figure 6.3).

A heat-map depicting the expression profile of non-clustered Pcdh genes in the brain, testis and brood pouch of the Manado pipefish at two developmental stages is shown in Supplementary Figure 6.4. Interestingly, the expression of *Pcdh8b1* and several other Pcdh genes (*Pcdh1b2*, *Pcdh12a1*, *Pcdh12a2* and *Pcdh17a*) is higher in the pregnant brood pouch than the non-pregnant brood pouch.


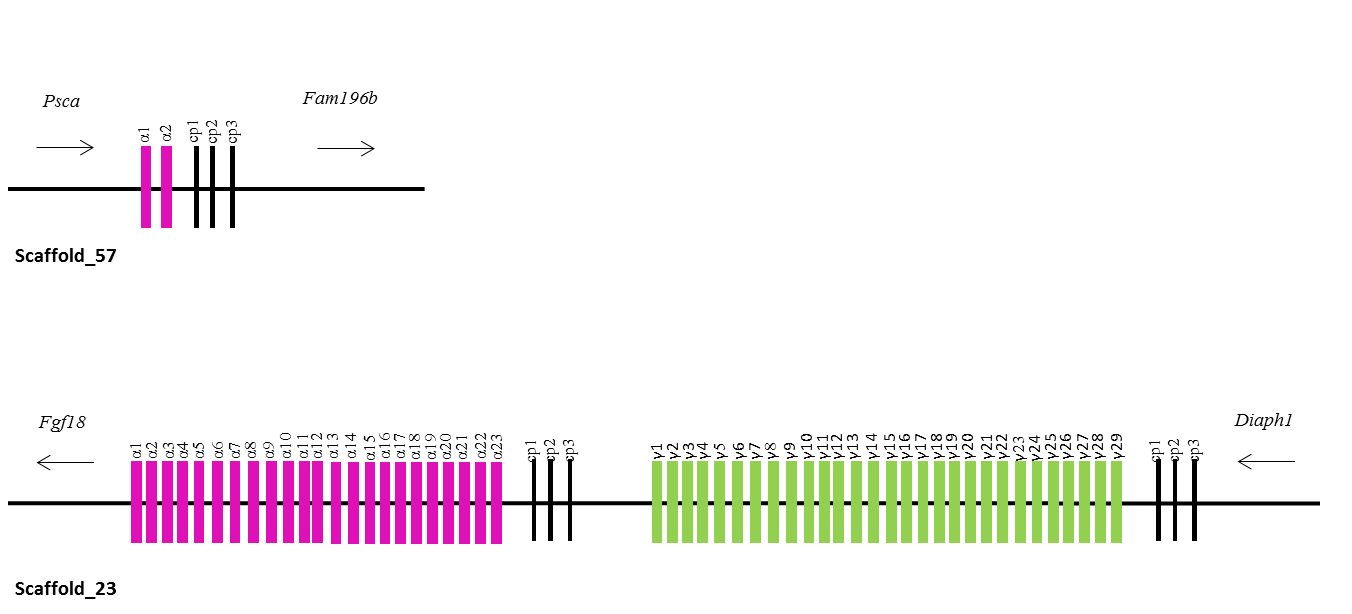


**Supplementary Figure 6.1** Genomic organization of the two Manado pipefish clustered Pcdh loci. Colored boxes represent variable exons whereas black bars at the end of each cluster represent the constant exons.


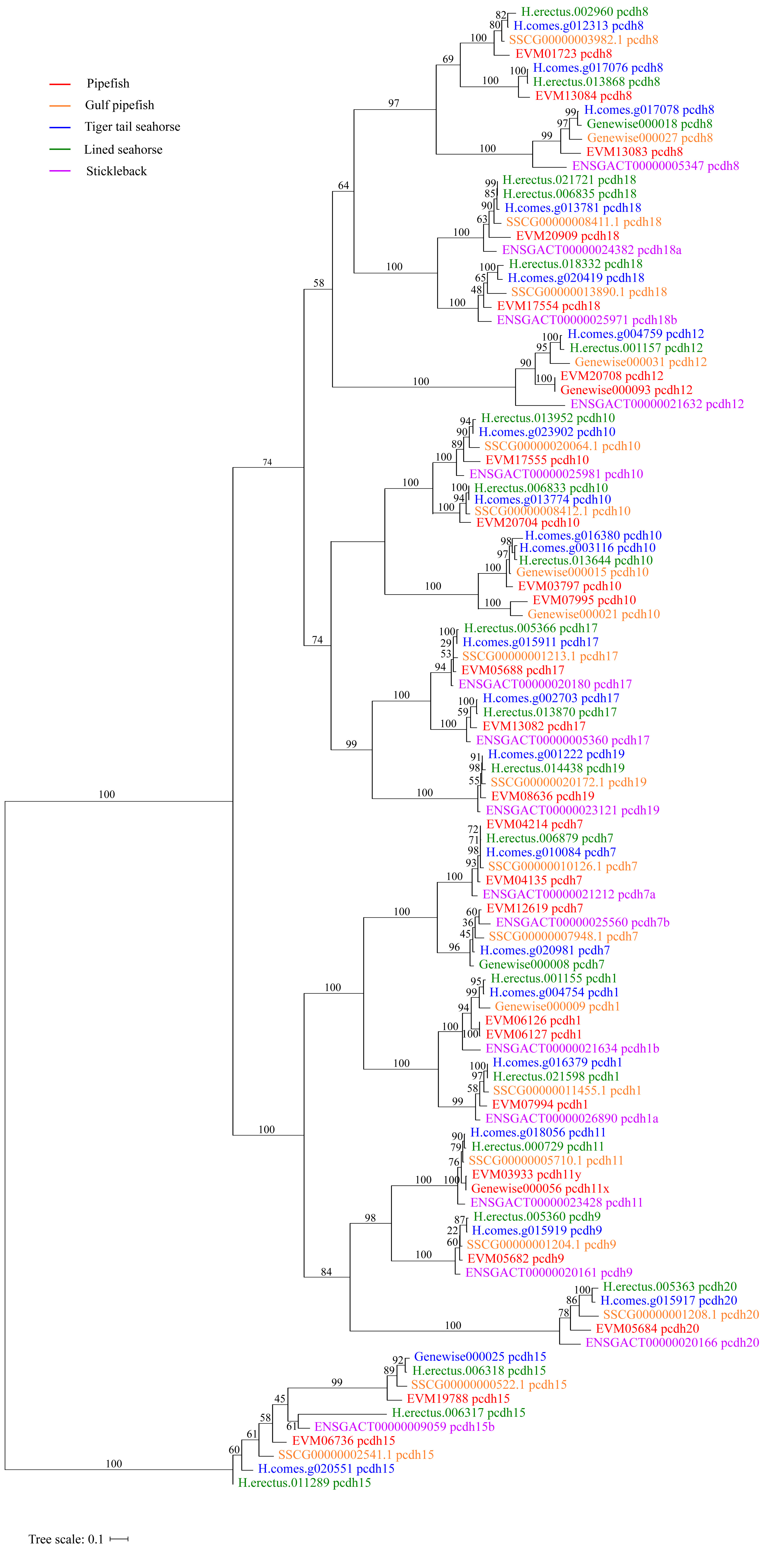


**Supplementary Figure 6.2** Phylogenetic relationship of non-clustered protocadherins. The phylogenetic tree was generated using full-length sequences from Manado pipefish, tiger tail seahorse, lined seahorse, gulf pipefish and stickleback.


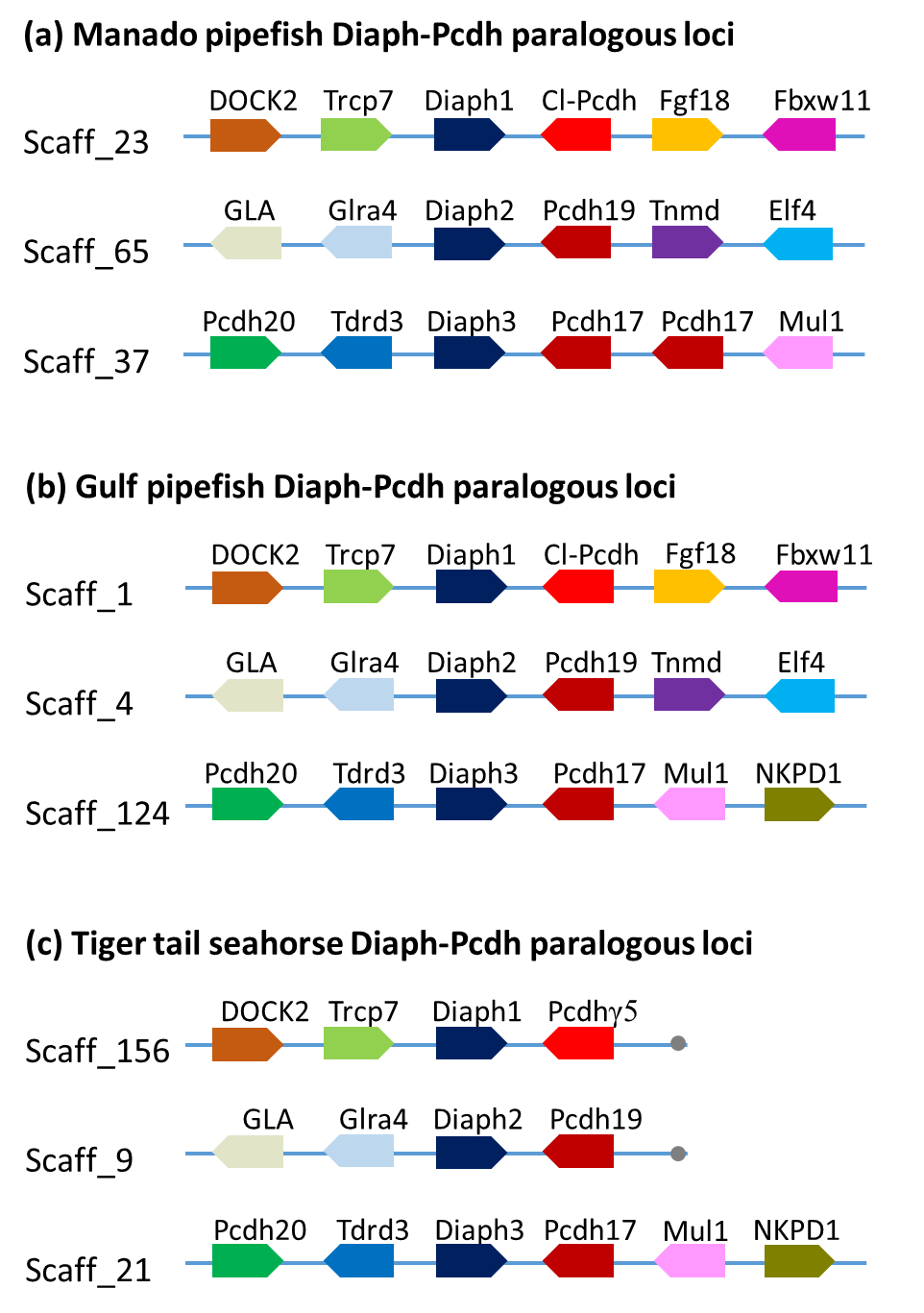


**Supplementary Figure 6.3** Diaph-Pcdh paralogous loci in Manado pipefish and other syngnathids. Genes are shown as block arrows with the direction of the arrows denoting the transcriptional orientation. Gray circles represent the ends of scaffolds. Cl-Pcdh denotes the clustered protocadherin locus.


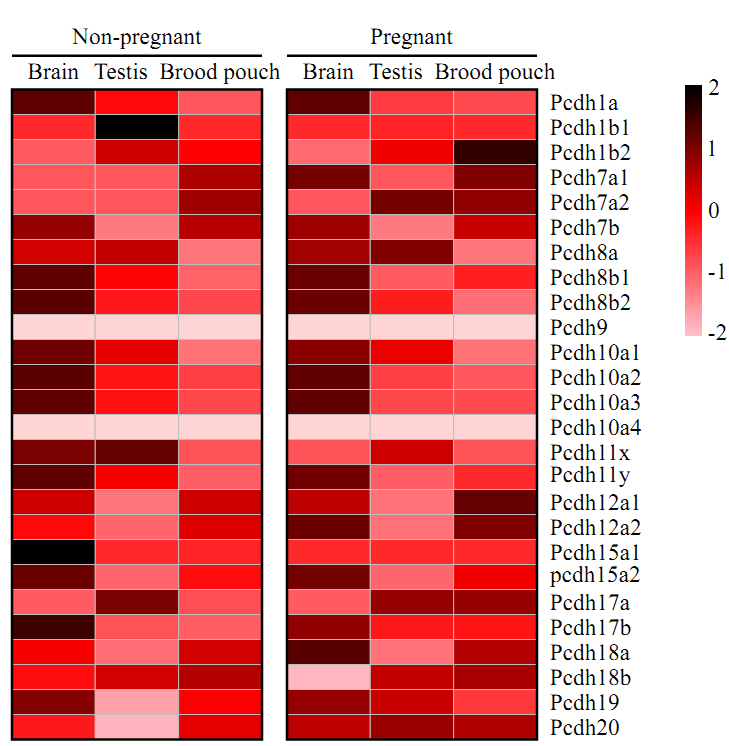


**Supplementary Figure 6.4** Expression profile of nonclustered Pcdh genes in the brain, testis and brood pouch at non-pregnant and pregnant stages of the Manado pipefish.

# 7. The SCPP gene family in the Manado pipefish

**Methods**

SCPP and related genes were identified in the genomes and/or transcriptomes of Manado pipefish, lined seahorse and gulf pipefish by TBLASTN using SCPP protein sequences from representative species (human, zebrafish, coelacanth and spotted gar). Identified regions of homology were used for BLASTX searches to confirm the identity of the genomic region. Predictions were also made using *ab* *initio* methods such as FGENESH [[35](#_ENREF_35)]. All predictions were checked and refined manually.

# 8. Convergent evolution

A total of 2,912 orthologous groups were obtained from 17 jawed vertebrate species (human: hg38, orangutan: ponAbe2, mouse: mm5, dog: canFam3, chicken: galGal5, anole lizard: anoCar2, turkey: melGal5, zebra finch: taeGut2, tiger-tail seahorse: H_comes_QL1_v1, lined seahorse, gulf pipefish, Manado pipefish, Nile tilapia: oreNil2, platyfish: Xipmac4.4.2, stickleback: gasAcu1, medaka: oryLat2, elephant shark: calMil1) based on syntenic pairwise alignments and reciprocal best hits. Amino acid alignments were generated for these orthologous groups using MUSCLE v3.8.31 [[36](#_ENREF_36)]. RAxML v8.2.4 [[37](#_ENREF_37)] was used to reconstruct maximum likelihood phylogenetic trees. In order to detect convergent signatures between syngnathids and mammals at a genome-wide level, we used the previously published symmetric design method [[38](#_ENREF_38)]. The designed phylogenetic topology consists of syngnathid species and placental mammals as foreground convergent clades, each of which comprises four species. An equal number of aplacental species were selected which formed sister clades to the placental mammal and syngnathid clades. The character state in the outgroup elephant shark was used to define the ancestral state. A derived character that is different from the ancestral state but shared by at least three syngnathids and three mammals is considered as a convergent site. We used the ‘convergence at conservative sites’ (CCS) approach to find the convergent sites [[38](#_ENREF_38)]. We used Gblocks to identify conservative loci, and then selected conservative sites within each of these loci. The criteria for inferring convergence are given in Figure 6a. Conservative sites are defined as sites where either the eight aplacental species or the syngnathid teleosts and placental mammals have the same character as the outgroup, i.e., A_1-1_=A_1-2_=A_1-3_=A_1-4_=A_2-1_=A_2-2_=A_2-3_=A_2-4_=O or P_1-1_=P_1-2_=P_1-3_=P_1-4_=P_2-1_=P_2-2_=P_2-3_=P_2-4_=O. If these criteria were met, we assumed that the ancestral state of the two clades were the same as the outgroup. This assumption is true in nearly all conservative sites, as we show below. With ancestors inferred as O, convergence was inferred if at least six of the eight other species share a derived character that was different from the ancestral state, i.e., P_1-i_=P_1-j_=P_1-k_=P_2-i_=P_2-j_=P_2-k_≠O or A_1-i_=A_1-j_=A_1-k_=A_2-i_=A_2-j_=A_2-k_≠O.

The ancestral character is very important as the number of convergent sites in the experimental and control groups depend on the ancestral character. Thus, we further estimated the accuracy of the inferred ancestral character in the placental mammal and syngnathid clades. For this, we performed amino acid simulation using the evolver program (PAML package) [36] under the JTT+gamma amino acid substitution model based on amino acid frequency, tree topology and branch length obtained using the codeml program [36]. In simulation of sequences using *evolver*, both present and ancestral states are known. We firstly identified conservative sites of simulated sequences in the outgroup using the same criteria as the CCS method; next, we compared the simulated state in the outgroup to the simulated state in the ancestral nodes. More than 99% of the ancestral characters could be correctly inferred at the conservative sites, suggesting that the 2,912 orthologs from 17 species were suitable for CCS analysis (Supplementary Figure 8.1).


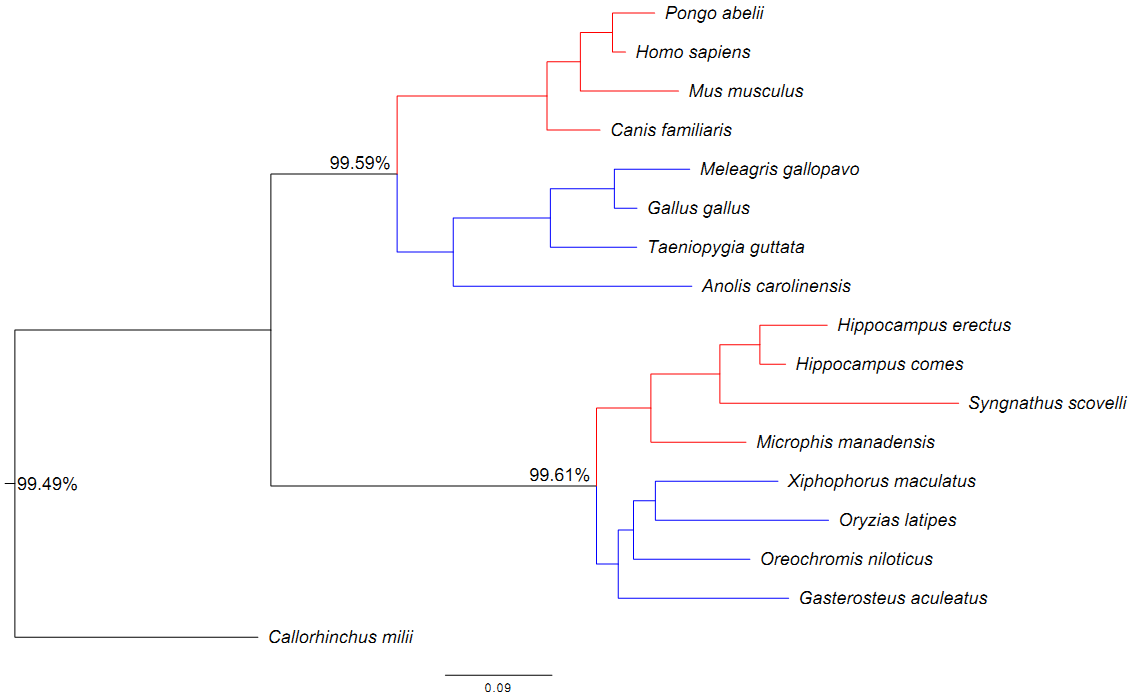


**Supplementary Figure 8.1** Accuracy of the ancestral character. Values estimated using *evolver* are shown at the ancestral nodes.

**Supplementary Table 8.1** Number of genes possessing convergent sites in placental and aplacental species.

| No. of convergent sites per gene | Placental species | Aplacental species | Placental/aplacental ratio |
| --- | --- | --- | --- |
| No. of genes with convergent sites in both foreground and background* | | | |
|  | 7 | 7 | 1 |
| No. of genes with convergent sites in foreground or background only | | | |
| ≥1(=1) | 111(105) | 29(29) | 3.83(3.62) |
| ≥2(=2) | 6(5) | 0(0) | undefined |
| ≥3 | 1 | 0 | undefined |
| Total number of sites | 118 | 29 | 4.07 |

*The same gene possesses different convergent sites in the foreground and the background

**Supplementary Table 8.2** The convergence/divergence test between the paired placental and the paired aplacental species.

| Placental Pairs versus Aplacental Pairs | Convergent Sites (C) | Divergent Sites (D) | C/D ratio | P-value |
| --- | --- | --- | --- | --- |
| Mammals and syngnathids | 125 | 292,495 | 0.0004274 | 0.00000002705 |
| Aplacental species | 36 | 249,575 | 0.0001442 |  |

**Supplementary Table 8.3** Convergent sites in the placental species.

| Gene | Gene ID | Position | Variation | Sites | Annotation |
| --- | --- | --- | --- | --- | --- |
| GPR26 | EVM10288 | 40 | V->A | VAAAAVVVVAAAAVVVV | PREDICTED: Stegastes partitus G protein-coupled receptor 26 |
|  | EVM10288 | 140 | V->A | VATAAVVVVAAAAVVVV |  |
|  | EVM10288 | 316 | S->R | SRRRRSSSSRRPRSSSS |  |
| SALL1 | EVM08301 | 350 | Q->H | QHHHHQQQQHNHHQQQQ | PREDICTED: Stegastes partitus spalt-like transcription factor 1 |
|  | EVM08301 | 351 | L->V | LAVVVLLLLVVVVLLLL |  |
| ASK1 | EVM12066 | 401 | D->E | DEEEEDDDDEEEEDDDD | PREDICTED: Stegastes partitus mitogen-activated protein kinase kinase kinase 5 |
|  | EVM12066 | 614 | K->R | KRRRRKKKKRRRRKKKK |  |
| NDRG1 | EVM11300 | 206 | I->V | IVVVVIIIIVVVVIIII | PREDICTED: Stegastes partitus protein NDRG1-like |
|  | EVM11300 | 293 | V->I | VIIIIVVVVIIIIVVVV |  |
| TTC21B | EVM14187 | 405 | E->D | EDDEDEEEEDDDDEEEE | PREDICTED: Larimichthys crocea tetratricopeptide repeat domain 21B |
|  | EVM14187 | 633 | K->R | KRRLRKKKKRKRRKKKK |  |
| AASS | EVM12818 | 704 | F->Y | FYYYYFFFFYYYYFFFF | PREDICTED: Stegastes partitus aminoadipate-semialdehyde synthase |
|  | EVM12818 | 823 | A->S | ASSSSAAAASSSSAAAA |  |
| ADAM12 | EVM10272 | 515 | I->V | IVVVVIIIIVVVVIIII | PREDICTED: Stegastes partitus ADAM metallopeptidase domain 12 |
| VASH1 | EVM04441 | 153 | N->S | NSSSSNNNNSSSSNNNN | PREDICTED: Larimichthys crocea vasohibin 1 |
| GDNF | EVM03680 | 209 | T->S | TSSSSTTTTSSSSTTTT | PREDICTED: Larimichthys crocea glial cell derived neurotrophic factor |
| MED1 | EVM16181 | 1284 | G->S | GSSSSGGGGSSNSGGGG | PREDICTED: Larimichthys crocea mediator complex subunit 1 |
| TMEM63C | EVM04339 | 87 | V->I | VIVIIVVVVIIIIVVVV | PREDICTED: Larimichthys crocea transmembrane protein 63C |
| CPXM2 | EVM10287 | 474 | H->Y | HYYYYHHHHYYYYHHHH | PREDICTED: Stegastes partitus carboxypeptidase X (M14 family), member 2 |
| MGAM | EVM07271 | 271 | Y->F | YFFFYYYYYFFFYYYYY | PREDICTED: Larimichthys crocea maltase-glucoamylase, intestinal-like |
| FAT4 | EVM06115 | 568 | K->R | KRRRRKKKKRRRRKKKK | PREDICTED: Stegastes partitus FAT atypical cadherin 4 |
| Col12A1 | EVM04429 | 1964 | L->V | LVVVVLLLLVLVVLLLL | PREDICTED: Larimichthys crocea collagen alpha-1(XII) chain-like |
| TMEM151B | EVM10620 | 61 | K->T | KTTTTKKKKTTTTKKKK | PREDICTED: Notothenia coriiceps transmembrane protein 151B |
| MID1 | EVM15043 | 533 | T->S | TSSSSTTTTSSSSTTTT | PREDICTED: Stegastes partitus midline 1 (Opitz/BBB syndrome) |
| TAF2 | EVM19646 | 686 | V->I | VIIIIVVVVIIIIVVVV | PREDICTED: Larimichthys crocea TAF2 RNA polymerase II, TATA box binding protein (TBP)-associated factor |
| Ralgapa2 | EVM17330 | 1585 | P->S | PSTSSPPPPSSSSPPPP | PREDICTED: Stegastes partitus Ral GTPase activating protein, alpha subunit 2 |
| Golga3 | EVM00011 | 1256 | V->I | VIIIIVVVVIIIIVVVV | PREDICTED: Larimichthys crocea golgin A3 |
| UBR2 | EVM07653 | 1393 | L->F | LFFFFLLLLFFFFLLLL | PREDICTED: Larimichthys crocea ubiquitin protein ligase E3 component n-recognin 2 |
| SEC23B | EVM15787 | 441 | I->V | IVVVVIIIIVVVVIIII | PREDICTED: Stegastes partitus Sec23 homolog B (S. cerevisiae) |
| SEC24D | EVM10440 | 563 | E->D | EDEDDEEEENDDDEEEE | PREDICTED: Stegastes partitus SEC24 family member D |
| Prkaa2 | EVM01690 | 296 | R->K | RKKKKRRRRKKKKRRRR | PREDICTED: Larimichthys crocea protein kinase, AMP-activated, alpha 2 catalytic subunit |
| Anapc4 | EVM02665 | 104 | T->S | TSSSSTTTTSSSSTTTT | PREDICTED: Larimichthys crocea anaphase promoting complex subunit 4 |
| RTF1 | EVM08696 | 122 | E->D | EDEDDEEEEDDDDEEEE | PREDICTED: Stegastes partitus Rtf1, Paf1/RNA polymerase II complex component, homolog (S. cerevisiae) |
| MMP2 | EVM08290 | 56 | K->T | KTTTTKKKKTTTTKKKK | PREDICTED: Larimichthys crocea matrix metallopeptidase 2 (gelatinase A, 72kDa gelatinase, 72kDa type IV collagenase) |
| CILP | EVM13812 | 508 | S->T | STTTTSSSSTTTTSSSS | PREDICTED: Larimichthys crocea cartilage intermediate layer protein, nucleotide pyrophosphohydrolase |
| NIN | EVM17723 | 431 | I->V | IVVVVIIIIVVVVIIII | PREDICTED: Larimichthys crocea ninein (GSK3B interacting protein) |
| Ifih1 | EVM14176 | 661 | E->Q | EQQQQEEEEQQQQEEEE | PREDICTED: Stegastes partitus interferon induced with helicase C domain 1 |
| Tbc1d24 | EVM06689 | 560 | V->A | VAAAAVVVVAAAAVVVV | PREDICTED: Larimichthys crocea TBC1 domain family, member 24 |
| CDH2 | EVM03850 | 512 | S->T | STTTTSSSSTTTTSSSS | PREDICTED: Larimichthys crocea cadherin 2, type 1, N-cadherin |
| Kcnq3 | EVM02730 | 624 | E->D | EDDDDEEEEDDDDEEEE | PREDICTED: Stegastes partitus potassium voltage-gated channel, KQT-like subfamily, member 3 |
| Sept6 | EVM08630 | 425 | R->K | RKKKKRRRRKKKKRRRR | PREDICTED: Notothenia coriiceps septin 6 |
| CLUH | EVM07288 | 1318 | N->S | NSSSSNNNNSSSSNNNN | PREDICTED: Stegastes partitus clustered mitochondria (cluA/CLU1) homolog |
| NPC1 | EVM19185 | 289 | G->A | GAAAAGGGGAAAAGGGG | PREDICTED: Stegastes partitus Niemann-Pick disease, type C1 |
| cTAGE5 | EVM00731 | 1751 | S->A | SAAAASSSSAAAASSSS | PREDICTED: Larimichthys crocea CTAGE family, member 5 |
| Klli31 | EVM03458 | 416 | N->S | NSSSSNNNNSSSSNNNN | PREDICTED: Larimichthys crocea kelch-like family member 31 |
| V1aR | EVM14936 | 172 | S->T | STTTTSSSSTTTTSSSS | Halichoeres trimaculatus V1a mRNA for arginine vasotocin receptor |
| Hdlbp | EVM07012 | 631 | V->I | VIIIIVVVVIIIIVVVV | PREDICTED: Larimichthys crocea high density lipoprotein binding protein |
| IPO 13 | EVM07096 | 639 | D->E | DEEEEDDDDEEEEDDDD | PREDICTED: Stegastes partitus importin-13-like |
| SACS | EVM13117 | 1067 | E->D | EDDDDEEEEDDDDEEEE | PREDICTED: Larimichthys crocea sacsin molecular chaperone |
| TULP4 | EVM18394 | 1530 | D->E | DEEEEDDDDEEEEDDDD | PREDICTED: Larimichthys crocea tubby-related protein 4-like |
| CASQ2 | EVM10345 | 179 | H->Y | HYYYYHHHHYYHYHHHH | PREDICTED: Stegastes partitus calsequestrin-2-like |
| NRG2 | EVM01247 | 51 | K->R | KRRRRKKKKGRRRKKKK | PREDICTED: Pundamilia nyererei pro-neuregulin-2, membrane-bound isoform-like |
| DEF6 | EVM15288 | 380 | Q->E | QEEEEQQQQEEEEQQQQ | PREDICTED: Stegastes partitus differentially expressed in FDCP 6 homolog (mouse) |
| TAS1R1 | EVM15656 | 324 | V->I | VIIIIVVVVIIIIVVVV | PREDICTED: Stegastes partitus taste receptor type 1 member 1-like |
| SLC25A15 | EVM04781 | 196 | T->S | TSSSSTTTTSSSSTTTT | PREDICTED: Stegastes partitus mitochondrial ornithine transporter 1-like |
| DUOX1 | EVM00989 | 1129 | Y->F | YFYFFYYYYFFFFYYYY | PREDICTED: Larimichthys crocea dual oxidase 1-like |
| UGT8 | EVM05580 | 38 | L->M | LMLMMLLLLMMMMLLLL | PREDICTED: Larimichthys crocea UDP glycosyltransferase 8 |
| Ush1g | EVM11430 | 428 | K->R | KRRRRKKKKRRRRKKKK | PREDICTED: Stegastes partitus Usher syndrome type-1G protein homolog |
| sucB | EVM04372 | 400 | A->T | ATTTTAAAATTTTAAAA | PREDICTED: Stegastes partitus dihydrolipoyllysine-residue succinyltransferase component of 2-oxoglutarate dehydrogenase complex, mitochondrial-like |
| Tbc1d32 | EVM00286 | 11 | S->A | SAAAASSSSAAASSSSS | PREDICTED: Stegastes partitus TBC1 domain family, member 32 |
| IPO5 | EVM17593 | 675 | A->S | ASSSSAAAASSSSAAAA | PREDICTED: Larimichthys crocea importin 5 |
| Sub1 | EVM13148 | 91 | Q->P | QPSPPQQQQSPPPQQQQ | PREDICTED: Larimichthys crocea SUB1 homolog (S. cerevisiae) |
| Vps13d | EVM03207 | 2497 | I->V | IVVVVIIIIVVVVIIII | PREDICTED: Stegastes partitus vacuolar protein sorting 13 homolog D (S. cerevisiae) |
| Scarb1 | EVM17159 | 214 | N->D | NDNDDNNNNDNDDNNNN | PREDICTED: Stegastes partitus scavenger receptor class B, member 1 |
| GDE1 | EVM13929 | 240 | F->Y | FYYYYFFFFYYYYFFFF | PREDICTED: Stegastes partitus glycerophosphodiester phosphodiesterase 1 |
| SCP2 | EVM01853 | 311 | A->G | AGGGGAAAAGGGGAAAA | PREDICTED: Larimichthys crocea sterol carrier protein 2 |
| Hsdl2 | EVM17053 | 330 | G->A | GAAAAGGGGAAAAGGGG | PREDICTED: Xiphophorus maculatus hydroxysteroid dehydrogenase-like protein 2-like |
| Adck1 | EVM08699 | 149 | E->D | EDDDDEEEEDDDDEEEE | PREDICTED: Larimichthys crocea aarF domain containing kinase 1 |
| Kiaa1432 | EVM03701 | 1224 | T->S | TSSSSTTTTSSSSTTTT | PREDICTED: Stegastes partitus KIAA1432 ortholog |
| TMEM175 | EVM14026 | 294 | I->V | IVVVVIIIIVVVVIIII | PREDICTED: Oryzias latipes transmembrane protein 175-like |
| Vgll3 | EVM10905 | 127 | F->L | FLLLLFFFFLLLLFFFF | PREDICTED: Larimichthys crocea vestigial-like family member 3 |
| APC2 | EVM07189 | 767 | K->R | KRRRRKKKKRRRRKKKK | PREDICTED: Stegastes partitus adenomatosis polyposis coli 2 |
| Eif4h | EVM16917 | 94 | E->D | EDDDDEEEEDEDDEEEE | PREDICTED: Neolamprologus brichardi eukaryotic translation initiation factor 4H-like |
| Srpr | EVM09374 | 351 | K->R | KRRRRKKKKRRRRKKKK | PREDICTED: Stegastes partitus signal recognition particle receptor (docking protein) |
| SLC46A1 | EVM07297 | 220 | L->I | LIIIILLLLIIIILLLL | PREDICTED: Notothenia coriiceps solute carrier family 46 (folate transporter), member 1 |
| Txndc16 | EVM19493 | 261 | V->L | VLLLLVVVVLLLLVVVV | PREDICTED: Stegastes partitus thioredoxin domain containing 16 |
| PEPD | EVM04541 | 140 | P->S | PSSSSPPPPSSSSPPPP | PREDICTED: Stegastes partitus peptidase D |
| Rgs9bp | EVM09921 | 87 | E->D | EDDDDEEEEDDDDEEEE | PREDICTED: Stegastes partitus regulator of G protein signaling 9 binding protein |
| KDSR | EVM16041 | 263 | K->R | KRRRRKKKKRRRYKKKK | PREDICTED: Haplochromis burtoni 3-ketodihydrosphingosine reductase-like |
| Lclat1 | EVM02165 | 73 | G->A | GAAAAGGGGAAAAGGGG | PREDICTED: Larimichthys crocea lysocardiolipin acyltransferase 1 |
| Pdzk1 | EVM10365 | 147 | L->V | LVVVVLLLLIVVVLLLL | PREDICTED: Notothenia coriiceps PDZ domain containing 1 |
| Ambra1 | EVM09689 | 518 | P->S | PSSSSPPPPSSSSPPPP | PREDICTED: Stegastes partitus autophagy/beclin-1 regulator 1 |
| Syt1 | EVM12822 | 90 | K->R | KRKRRKKKKRRRRKKKK | PREDICTED: Stegastes partitus synaptotagmin-1-like |
| PLK4 | EVM04487 | 623 | C->S | CSSSSCCCCSSSSCCCC | PREDICTED: Larimichthys crocea polo-like kinase 4 |
| EMC1 | EVM15599 | 493 | N->T | NTTTTNNNNTTTTNNNN | PREDICTED: Larimichthys crocea ER membrane protein complex subunit 1 |
| Fam83c | EVM18122 | 245 | K->R | KRRRRKKKKRRRRKKKK | PREDICTED: Larimichthys crocea family with sequence similarity 83, member C |
| DCN | EVM00416 | 218 | K->Q | KQQQQKKKKQKQQKKKK | Paralichthys olivaceus decorin |
| Trap1 | EVM17811 | 286 | E->D | EDDDDEEEEGDDDEEEE | PREDICTED: Stegastes partitus TNF receptor-associated protein 1 |
| SLC39A1 | EVM08821 | 388 | D->N | DNNNNDDDDNNNNDDDD | PREDICTED: Larimichthys crocea zinc transporter 1-like |
| SLC30A7 | EVM07177 | 360 | I->V | IVVVVIIIIVVVVIIII | PREDICTED: Poecilia formosa solute carrier family 30 (zinc transporter), member 7 |
| TBCD | EVM12384 | 655 | I->V | ILVVVIIIIVVVVIIII | PREDICTED: Larimichthys crocea tubulin folding cofactor D |
| RAB3B | EVM07200 | 206 | T->S | TSSSSTTTTSCSSTTTT | PREDICTED: Oreochromis niloticus ras-related protein Rab-3B-like |
| CEP120 | EVM07433 | 676 | A->P | APPPPAAAAPAPPAAAA | PREDICTED: Stegastes partitus centrosomal protein 120kDa |
| MLX | EVM19013 | 152 | M->T | MTTTTMMMMTTTTMMMM | PREDICTED: Larimichthys crocea MLX, MAX dimerization protein |
| Scpep1 | EVM17841 | 190 | L->V | LVVVVLLLLAVVVLLLL | PREDICTED: Larimichthys crocea serine carboxypeptidase 1 |
| TOR4A | EVM19539 | 228 | K->R | KRHRRKKKKRRRRKKKK | PREDICTED: Poecilia reticulata torsin family 4, member A |
| Ncapg | EVM13827 | 482 | E->A | EAAAAEEEEAAAAEEEE | PREDICTED: Stegastes partitus non-SMC condensin I complex, subunit G |
| RNF13 | EVM04241 | 81 | V->I | VIIIIVVVVILIIVVVV | PREDICTED: Larimichthys crocea ring finger protein 13 |
| Guca1b | EVM03119 | 54 | A->S | ATSSSAAAASSSSAAAA | PREDICTED: Stegastes partitus guanylate cyclase activator 1B |
| Zgpat | EVM09198 | 242 | S->N | SNNNNSSSSNNNNSSSS | PREDICTED: Larimichthys crocea zinc finger, CCCH-type with G patch domain |
| Gucd1 | EVM06393 | 179 | C->R | CRRRRCCCCRRRRCCCC | PREDICTED: Larimichthys crocea guanylyl cyclase domain containing 1 |
| EP400 | EVM01365 | 975 | S->I | SIIIISSSSIIIISSSS | PREDICTED: Stegastes partitus E1A binding protein p400 |
| WDR66 | EVM02324 | 354 | I->V | IVVVVIIIIVLVVIIII | PREDICTED: Notothenia coriiceps WD repeat domain 66 |
| Cpsf3l | EVM15736 | 339 | Q->H | QHQHHQQQQRHHHQQQQ | PREDICTED: Larimichthys crocea cleavage and polyadenylation specific factor 3-like |
| Plek2 | EVM14679 | 301 | C->S | CSSSSCCCCSSSSCCCC | PREDICTED: Larimichthys crocea pleckstrin 2 |
| SIX4 | EVM04508 | 639 | E->D | EDDDDEEEEDDDDEEEE | PREDICTED: Larimichthys crocea SIX homeobox 4 |
| AP5M1 | EVM04334 | 172 | L->F | LFLFFLLLLFFFFLLLL | PREDICTED: Larimichthys crocea adaptor-related protein complex 5, mu 1 subunit |
| RPAP2P1 | EVM14618 | 1158 | L->V | LVVVVLLLLVVVVLLLL | PREDICTED: Pundamilia nyererei RNA polymerase II-associated protein 1-like |
| CIRH1A | EVM05956 | 140 | L->F | LFFFFLLLLFFFFLLLL | Gasterosteus aculeatus clone CNB255-G04 |
| Dopey2 | EVM10348 | 1726 | E->D | EDDDDEEEEDDDDEEEE | PREDICTED: Stegastes partitus dopey family member 2 |
| MED14 | EVM08787 | 360 | S->F | SFFFFSSSSFSFFSSSS | PREDICTED: Larimichthys crocea mediator complex subunit 14 |
| CPN1 | EVM16155 | 200 | L->R | LQRRRLLLLRRRRLLLL | PREDICTED: Stegastes partitus carboxypeptidase N, polypeptide 1 |
| Ankrd1 | EVM00906 | 167 | V->I | VIIIIVVVVIIIIVVVV | PREDICTED: Larimichthys crocea ankyrin repeat domain 1 (cardiac muscle) |
| A1cf | EVM14488 | 244 | T->S | TSSSSTTTTSSSSTTTT | PREDICTED: Larimichthys crocea APOBEC1 complementation factor |
| OAT | EVM10285 | 261 | K->R | KQRRRKKKKRQRRKKKK | PREDICTED: Larimichthys crocea ornithine aminotransferase |
| Lipt1 | EVM18653 | 141 | V->I | VVIIIVVVVIIIIVVVV | PREDICTED: Larimichthys crocea lipoyltransferase 1 |
| Iqub | EVM12812 | 465 | A->S | ASSSSAAAASSSSAAAA | PREDICTED: Larimichthys crocea IQ motif and ubiquitin domain containing |
| Klhl32 | EVM08337 | 181 | D->E | DEEEEDDDDEEEEDDDD | PREDICTED: Stegastes partitus kelch-like family member 32 |
| Dopey1 | EVM08350 | 609 | V->I | VIIIIVVVVIIIIVVVV | PREDICTED: Stegastes partitus dopey family member 1 |
| Ibtk | EVM08354 | 287 | V->I | VIIIIVVVVIIIIVVVV | PREDICTED: Larimichthys crocea inhibitor of Bruton agammaglobulinemia tyrosine kinase |
| Alkbh1 | EVM00374 | 165 | T->S | TSSSSTTTTSSSSTTTT | PREDICTED: Larimichthys crocea alkB, alkylation repair homolog 1 (E. coli) |
| RGR | EVM00922 | 71 | I->V | IVVVVIIIIVVVVIIII | PREDICTED: Stegastes partitus retinal G protein coupled receptor |
| Asnsd1 | EVM06148 | 502 | L->I | LIIIILLLLIIIILLLL | PREDICTED: Stegastes partitus asparagine synthetase domain containing 1 |
| Pms1 | EVM08504 | 167 | E->D | EDDDDEEEEDDEDEEEE | PREDICTED: Oreochromis niloticus PMS1 postmeiotic segregation increased 1 (S. cerevisiae) |
| ADPS | EVM10885 | 49 | L->V | LVVVVLLLLVVVVLLLL | PREDICTED: Haplochromis burtoni alkyldihydroxyacetonephosphate synthase, peroxisomal-like |

**Supplementary Table 8.4** GO enrichment test of convergent genes in the placental species (P-value<0.05, FDR<0.5).

| GO_ID | GO_Term | GO_Class | Gene number | P-value | FDR |
| --- | --- | --- | --- | --- | --- |
| GO:0008610 | Lipid biosynthetic process | BP | 2 | 0.00078 | 0.11277 |
| GO:0010468 | Regulation of gene expression | BP | 2 | 0.00273 | 0.39655 |
| GO:0030127 | COPII vesicle coat | CC | 2 | 0.00036 | 0.01239 |
| GO:0042995 | Cell projection | CC | 2 | 0.00590 | 0.20045 |
| GO:0045252 | Oxoglutarate dehydrogenase complex | CC | 1 | 0.00612 | 0.20820 |
| GO:0005785 | Signal recognition particle receptor complex | CC | 1 | 0.00612 | 0.20820 |
| GO:0032389 | Mutlalpha complex | CC | 1 | 0.01221 | 0.41515 |
| GO:0033270 | Paranode region of axon | CC | 1 | 0.01221 | 0.42515 |
| GO:0033268 | Node of Ranvier | CC | 1 | 0.01221 | 0.41515 |
| GO:0000796 | Condensin complex | CC | 1 | 0.01221 | 0.41515 |
| GO:0032934 | Sterol binding | MF | 2 | 0.00078 | 0.06330 |
| GO:0004181 | Metallocarboxypeptidase activity | MF | 2 | 0.00596 | 0.48272 |

BP: Biological process. CC: Cellular Component. MF: Molecular Function.

# References

1. Nelson JS, Grande TC and Wilson MVH. *Fishes of the World*: Wiley; 2016.

2. Hamilton H, Saarman N, Short G *et al.* Molecular phylogeny and patterns of diversification in syngnathid fishes. *Mol Phylogenet Evol*. 2017; **107**: 388-403.

3. Li RQ, Fan W, Tian G *et al.* The sequence and de novo assembly of the giant panda genome. *Nature*. 2010; **463**(7279): 311-7.

4. Gnerre S, MacCallum I, Przybylski D *et al.* High-quality draft assemblies of mammalian genomes from massively parallel sequence data. *Proc Natl Acad Sci U S A*. 2011; **108**(4): 1513-8.

5. Boetzer M, Henkel CV, Jansen HJ *et al.* Scaffolding pre-assembled contigs using SSPACE. *Bioinformatics*. 2011; **27**(4): 578-9.

6. Luo R, Liu B, Xie Y *et al.* SOAPdenovo2: an empirically improved memory-efficient short-read de novo assembler. *Gigascience*. 2012; **1**(1): 18.

7. Trapnell C, Pachter L and Salzberg SL. TopHat: discovering splice junctions with RNA-Seq. *Bioinformatics*. 2009; **25**(9): 1105-11.

8. Trapnell C, Williams BA, Pertea G *et al.* Transcript assembly and quantification by RNA-Seq reveals unannotated transcripts and isoform switching during cell differentiation. *Nat Biotechnol*. 2010; **28**(5): 511-U174.

9. Edgar RC and Myers EW. PILER: identification and classification of genomic repeats. *Bioinformatics*. 2005; **21**: I152-I8.

10. Price AL, Jones NC and Pevzner PA. De novo identification of repeat families in large genomes. *Bioinformatics*. 2005; **21**: I351-I8.

11. Xu Z and Wang H. LTR_FINDER: an efficient tool for the prediction of full-length LTR retrotransposons. *Nucleic Acids Res*. 2007; **35**: W265-W8.

12. Jurka J, Kapitonov VV, Pavlicek A *et al.* Repbase update, a database of eukaryotic repetitive elements. *Cytogenet Genome Res*. 2005; **110**(1-4): 462-7.

13. Bao ZR and Eddy SR. Automated de novo identification of repeat sequence families in sequenced genomes. *Genome Res*. 2002; **12**(8): 1269-76.

14. Kimura M. A Simple Method for Estimating Evolutionary Rates of Base Substitutions through Comparative Studies of Nucleotide-Sequences. *J Mol Evol.* 1980; **16**(2): 111-20.

15. Lampe DJ, Churchill ME and Robertson HM. A purified mariner transposase is sufficient to mediate transposition in vitro. *The EMBO J*. 1996; **15**(19): 5470-9.

16. Plasterk RH, Izsvak Z and Ivics Z. Resident aliens: the Tc1/mariner superfamily of transposable elements. *Trends in genetics : TIG*. 1999; **15**(8): 326-32.

17. Vos JC and Plasterk RH. Tc1 transposase of Caenorhabditis elegans is an endonuclease with a bipartite DNA binding domain. *The EMBO J*. 1994; **13**(24): 6125-32.

18. Wang D, Zhang Y, Zhang Z *et al.* KaKs_Calculator 2.0: a toolkit incorporating gamma-series methods and sliding window strategies. *Genom Proteom Bioin*. 2010; **8**(1): 77-80.

19. Hubbard T, Barker D, Birney E *et al.* The Ensembl genome database project. *Nucleic Acids Res*. 2002; **30**(1): 38-41.

20. Small CM, Bassham S, Catchen J *et al.* The genome of the Gulf pipefish enables understanding of evolutionary innovations. *Genome Biol*. 2016; **17**(1): 258.

21. Lin Q, Fan S, Zhang Y *et al.* The seahorse genome and the evolution of its specialized morphology. *Nature*. 2016; **540**(7633): 395-9.

22. Lin Q, Qiu Y, Gu R *et al.* Draft genome of the lined seahorse, Hippocampus erectus. *GigaScience*. 2017; **6**(6): 1-6.

23. You X, Bian C, Zan Q *et al.* Mudskipper genomes provide insights into the terrestrial adaptation of amphibious fishes. *Nat Commun*. 2014; **5**: 5594.

24. Altschul SF, Gish W, Miller W *et al.* Basic local alignment search tool. *J Mol Biol*. 1990; **215**(3): 403-10.

25. Bairoch A, . and Apweiler R, . The SWISS-PROT protein sequence database and its supplement TrEMBL in 2000. *Nucleic Acids Res*. 2000; **28**(1): 45.

26. Edgar RC. MUSCLE: a multiple sequence alignment method with reduced time and space complexity. *Bmc Bioinformatics*. 2004; **5**(5): 113.

27. Sievers F, Wilm A, Dineen D *et al.* Fast, scalable generation of high‐quality protein multiple sequence alignments using Clustal Omega. *Mol Syst Biol*. 2011; **7**(1): 539.

28. Price MN, Dehal PS and Arkin AP. FastTree 2 – Approximately Maximum-Likelihood Trees for Large Alignments. *Plos One*. 2010; **5**(5): e9490.

29. Wu Q and Maniatis T. A striking organization of a large family of human neural cadherin-like cell adhesion genes. *Cell*. 1999; **97**(6): 779-90.

30. Redies C, Vanhalst K and Fv R. delta-Protocadherins: unique structures and functions. *Cellul Mol Life Sci*. 2005; **62**(23): 2840-52.

31. Noonan JP, Grimwood J, Danke J *et al.* Coelacanth genome sequence reveals the evolutionary history of vertebrate genes. *Genome Res*. 2004; **14**(12): 2397.

32. Yu WP, Rajasegaran V, Yew K *et al.* Elephant shark sequence reveals unique insights into the evolutionary history of vertebrate genes: A comparative analysis of the protocadherin cluster. *Proc Natl Acad Sci U S A.* 2008; **105**(10): 3819-24.

33. Tada MN, Senzaki K, Tai Y *et al.* Genomic organization and transcripts of the zebrafish Protocadherin genes. *Gene*. 2004; **340**(2): 197-211.

34. Yu WP, Yew K, Rajasegaran V *et al.* Sequencing and comparative analysis of fugu protocadherin clusters reveal diversity of protocadherin genes among teleosts. *Bmc Evol Biol*. 2007; **7**(1): 49.

35. Solovyev V, Kosarev P, Seledsov I *et al.* Automatic annotation of eukaryotic genes, pseudogenes and promoters. *Genome Biol*. 2006; **7 Suppl 1**: S10 1-2.

36. Edgar RC. MUSCLE: multiple sequence alignment with high accuracy and high throughput. *Nucleic Acids Res*. 2004; **32**(5): 1792-7.

37. Stamatakis A. RAxML version 8: a tool for phylogenetic analysis and post-analysis of large phylogenies. *Bioinformatics*. 2014; **30**(9): 1312-3.

38. Xu S, He Z, Guo Z *et al.* Genome-Wide Convergence during Evolution of Mangroves from Woody Plants. *Mol Biol Evol*. 2017; **34**(4): 1008-15.
